# A *cis*-eQTL in *NSUN2* promotes esophageal squamous-cell carcinoma progression and radiochemotherapy resistance by mRNA-m^5^C methylation

**DOI:** 10.1038/s41392-022-01063-2

**Published:** 2022-08-08

**Authors:** Xiangjie Niu, Linna Peng, Weiling Liu, Chuanwang Miao, Xinjie Chen, Jiahui Chu, Xinyu Yang, Wen Tan, Chen Wu, Dongxin Lin

**Affiliations:** 1 Department of Etiology and Carcinogenesis, National Cancer Center/National Clinical Research Center/Cancer Hospital, Chinese Academy of Medical Sciences and Peking Union Medical College, Beijing, China; 2 CAMS Key Laboratory of Genetics and Genomic Biology, Chinese Academy of Medical Sciences and Peking Union Medical College, Beijing, China; 3 Jiangsu Collaborative Innovation Center for Cancer Personalized Medicine, Nanjing Medical University, Nanjing, China

**Keywords:** Cancer therapy, Molecular biology

**Dear Editor**,

Esophageal squamous cell carcinoma (ESCC) has poor prognosis because of the difficulty in early detection and low sensitivity of advanced disease to radiochemotherapy.^1,2^ ESCC presents a high proportion of primary resistance to radiochemotherapy,^2^ which may be due to certain individual genetic variations. Expression quantitative trait loci (eQTLs) as proximal and continuous cellular phenotypes have been shown to be helpful to determine how genetic variants may influence phenotype.^3^

We examined the ESCC tumor-specific eQTLs based on our previous whole-genome DNA and RNA sequencing data of 94 ESCC samples^4^ to discover eQTL-target genes involved in the malignant phenotypes of ESCC. By analyzing the mRNA levels of 376 genes targeted by ESCC-specific eQTLs, we found 10 genes that had significantly higher expression levels in tumor than in normal in all 94 samples, which could be verified in TCGA 90 ESCC samples and Oncomine 51 ESCC samples (Supplementary Fig. 1a and Supplementary Table 1). Among the 10 genes, silencing *NSUN2*, a gene encoding the 5-methylcytosine (m^5^C) RNA methyltransferase, had the most inhibitory effect on cell viability than others (Supplementary Fig. 1b). We found that NSUN2 levels were significantly higher in ESCC than adjacent normal tissues determined by immunohistochemical (IHC) staining and Western blotting (Fig. 1a; Supplementary Fig. 1c and Supplementary Table 2) and high level was correlated with shorter survival time in patients (Fig. 1b). The eQTL analysis indicated 7 candidate SNPs in the adjacent *SRD5A1* and *NSUN2* loci that might regulate *NSUN2* transcription (Supplementary Table 3) and these SNPs are all in high linkage disequilibrium (Supplementary Fig. 1d). Suggested by the functional annotation, 4 SNPs were selected for electrophoretic mobility shift assays (EMSA) and the results suggested that rs10076470 G > A mutation may create a functional *cis*-eQTL (Supplementary Fig. 1e). Reporter gene assays showed that the plasmid containing the rs10076470 A allele had significantly higher luciferase expression than the plasmid containing the rs10076470 G counterpart (Supplementary Fig. 1f). RNA-sequencing analysis revealed that ESCC tissues with the AA or AG genotype had significantly higher *NSUN2* RNA levels than that with the GG genotype (Fig. 1c). The rs10076470 A allele renders higher *NSUN2* expression was further found in ESCC cell lines (Supplementary Fig. 1g).

**Fig. 1:**
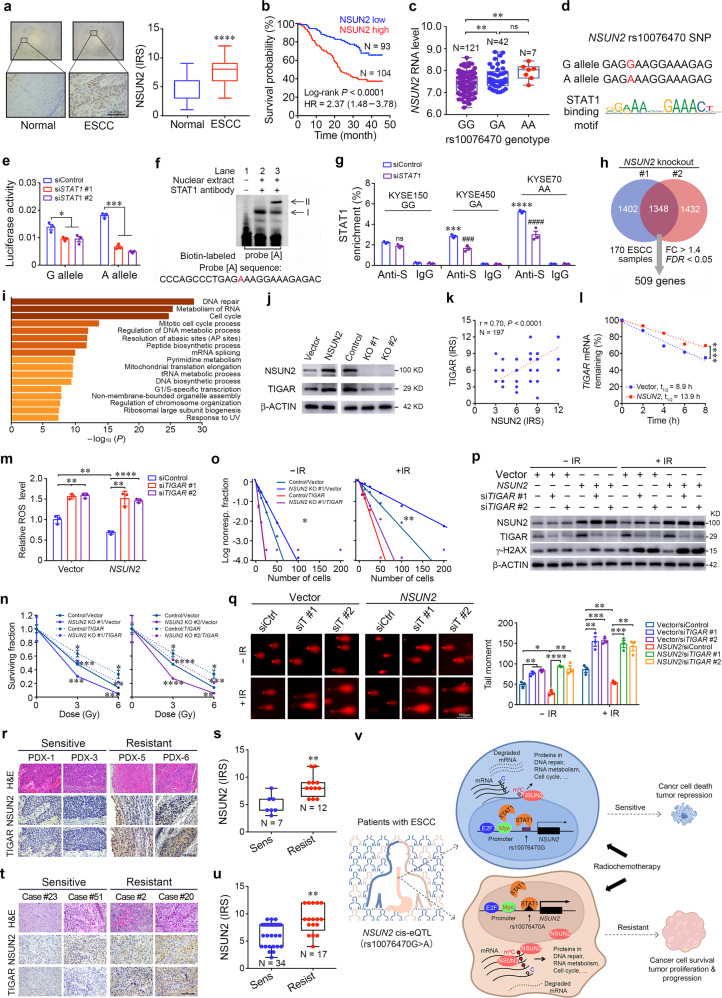
A *cis*-eQTL variant in *NSUN2* locus promotes esophageal squamous-cell carcinoma progression and radiochemotherapy resistance by mRNA-m^5^C modification of cancer-related genes. **a** Immunohistochemistry (IHC) analysis of NSUN2 protein levels in paired human esophageal squamous-cell carcinoma (ESCC) and adjacent normal tissues. *Left panel* shows representative IHC pictures of tissue arrays (scale bar, 100 μm) and *right panel* shows the statistics of IHC score (IRS) indicating that 85.3% (168/197) of ESCCs had significantly higher NSUN2 level compared with normal tissues. ****P < 0.0001 of Mann-Whitney test. IRS, immunoreactive score. **b** Kaplan-Meier survival curves for patients with high (IRS ≥ 6) or low (IRS < 6) NSUN2 level in ESCC. HR (95% CI) was computed with Cox hazard proportion model. **c** Comparison of *NSUN2* mRNA levels in ESCCs as function of the rs10076470 genotype, showing that the A allele (GA and AA genotypes) had significantly higher levels compared with the G allele. **P < 0.01 and ns, not significant of Mann-Whitney test. **d** The rs10076470 A allele resides within a potential STAT1 binding motif. **e** The effect of *STAT1* knockdown on luciferase activity of constructs containing the rs10076470 G or rs10076470 A allele in KYSE150 cells. Data are mean ± S.E.M. from 3 replicate experiments. *P < 0.05 and ***P < 0.001 of Student’s *t*-test. **f** Super-shift EMSA competition assay with STAT1 antibody and KYSE150 cell nuclear extract. I points the rs10076470A -specific band and II points a super-shifted band by STAT1. **g** Chromatin immunoprecipitation coupled qPCR analysis shows that STAT1 binds to *NSUN2* promoter in an allele-specific manner. Data are mean ± S.E.M. from 3 replicate experiments. Anti-S, anti STAT1 antibody. *** and ###*P* < 0.001; **** and ####*P* < 0.0001 and ns, not significant of Student’s *t*-test between si*STAT1* and siControl and among different genotypes. **h** Venn diagram displays 1348 overlapped genes among 2750 and 2780 apparently downregulated genes in cells with *NSUN2* knockout (KO) #1 and *NSUN2* KO #2, respectively. Among 1348 overlapped genes, 509 differentially expressed genes (DEGs) had higher mRNA levels in ESCCs than adjacent normal tissues (fold change >1.4, FDR < 0.05). **i** Metascape gene enrichment analysis of the 509 DEGs (http://metascape.org/gp/index.html#/main/step1). **j** Western blot analysis of NSUN2 and TIGAR protein levels in KYSE150 cells with *NSUN2* overexpression (OE) or KO. **k** Spearman correlation between NSUN2 and TIGAR protein levels expressed as IRS in ESCC. **l** The effect of *NSUN2* OE on the TIGAR mRNA stability in KYSE150 cells determined by RT-qPCR at indicated time points after treatment with 6 μM actinomycin D. Data are mean ± S.E.M. In most data points, the error bars are within the symbols. ****P < 0.0001 of Student’s *t*-test. **m** *NSUN2* OE significantly inhibited ROS production in KYSE150 cells, which could be rescued by silencing *TIGAR*. Data are mean ± S.E.M. from 3 experiments and each had 3 replications. **P < 0.01 and ****P < 0.0001 of Student’s *t*-test. **n** The effect of *NSUN2* KO on colony formation ability of KYSE150 cells with or without ionizing radiation (IR, 4 Gy) and *TIGAR* OE. Shown are mean ± S.E.M. from 3 experiments and each had 3 replications. In some data points, the error bars are within the symbols. *P < 0.05; **P < 0.01; ***P < 0.001 and *P* < 0.01; ****P < 0.0001 of Student’s *t*-test. **o** Extreme limiting dilution assays show survival fractions in KYSE150 cells with *NSUN2* KO caused by IR (4 Gy) and *TIGAR* OE. *P < 0.05 and **P < 0.01 of ELDA analysis program. **p** The effect of *NSUN2* OE on DNA double-strand breaks detected by γ-H2AX in KYSE150 cells with or without IR (4 Gy) and *TIGAR* knockdown. **q** The effect of *NSUN2* OE on DNA double-strand breaks detected by comet assays in KYSE150 cells with or without IR (4 Gy) and *TIGAR* knockdown. *Left panels* show fluorescence images of comet assays (scale bars, 100 μm) and *right panels* show the statistics. Data are mean ± S.E.M. from 3 replicates and 3 fields were randomly selected from each experiment. siCtrl, siControl; siT, siTIGAR. **r** Representative images showing NSUN2 and TIGAR immunohistochemical staining in ESCC establishing PDXs with differential radiosensitivity. Scale bars, 100 μm. **s** Box and bar plots comparing the NSUN2 protein levels in PDXs with differential radiosensitivity. Sens, sensitive and Resist, Resistance. **P < 0.01 of Mann-Whitney test. **t** Representative images showing NSUN2 and TIGAR immunohistochemical staining in ESCC biopsy specimens taken before adjuvant radiochemotherapy in patients with differential sensitivity. Scale bars, 100 μm. **u** Box and bar plots comparing the NSUN2 protein levels in ESCC biopsy specimens with differential sensitivity to adjuvant radiochemotherapy. **P < 0.01 of Mann-Whitney test. **v** The schematic illustration for the possible mechanisms of aberrant *NSUN2* expression and NSUN2-mediated radiochemo-resistance in ESCC. Part of the schematic illustration is generated from BioRender (https://biorender.com/).

We then sought for the transcriptional factors (TFs) that might interact with the *cis*-element formed by rs10076470 G/A SNP using JASPAR and HumanTFDB databases combined with the mRNA levels of these TFs in 170 paired tissue samples. The result suggested STAT1 as the potential candidate TF for the rs10076470 A allele (Fig. 1d and Supplementary Fig. 2a, b). Indeed, *STAT1* knockdown significantly suppressed *NSUN2* expression at both mRNA and protein levels in KYSE450 (GA genotype) and KYSE70 cells (AA genotype) but not in KYSE150 cells (GG genotype) (Supplementary Fig. 2c, d). Reporter gene assays showed that silencing *STAT1* significantly reduced the luciferase activity of A allele construct while G allele construct was much less affected (Fig. 1e and Supplementary Fig. 2e). The additional EMSA assays showed that nuclear protein bound to the DNA probe was rs10076470 A specific and the band could be super-shifted when STAT1 antibody was included in the incubation mixture (Fig. 1f and Supplementary Fig. 2f–h), indicating that the protein bound to the DNA is likely STAT1. ChIP-qPCR detection (Fig. 1g) showed significant STAT1 enrichment in KYSE450 and KYSE70 than in KYSE150. Furthermore, the *STAT1* and *NSUN2* RNA levels were positively correlated in 170 ESCC samples but this correlation was limited in those with the rs10076470 GA or AA genotype (Supplementary Fig. 2i).

Duplicate RNA sequencing on *NSUN2* knockout KYSE150 cells revealed downregulation of 2750 and 2780 genes compared with cells without the knockout (Supplementary Fig. 3a). Combined analysis showed overlap of 1348 genes, of which 509 genes had significant higher expression in 170 ESCC than in normal esophageal tissue samples (Fig. 1h and Supplementary Table 4). Pathway enrichment analysis of the 509 genes indicated that DNA repair, RNA metabolism and cell cycle pathways were the most significantly downregulated (Fig. 1i). We then selected the top 10 genes in these three pathways for verification by RT-qPCR in an independent knockout assay and the significant downregulation of almost all genes (27/30) were confirmed (Supplementary Fig. 3b–d). m^5^C-RNA immunoprecipitation coupled with qPCR analysis showed that *NSUN2* knockout significantly decreased m^5^C-mRNA levels of almost all these tested genes in cells compared with the levels in control cells (Supplementary Fig. 4a, b). The measurement of the m^5^C global levels in ESCC cells showed consistent results showing that the m^5^C levels were substantially increased with *NSUN2* overexpression and decreased with *NSUN2* knockout (Supplementary Fig. 4c, d).

Since *TIGAR*, among other genes, was the most significantly downregulated and its mRNA m^5^C level was significantly decreased upon *NSUN2* knockout, it was chosen as an example of NSUN2 affected transcript for further investigation. Disruption of *NSUN2* expression had significant impact on *TIGAR* expression at both mRNA and protein levels (Fig. 1j and Supplementary Fig. 5a–d). IHC staining of esophageal tissue samples from *Nsun2*^+/+^ mice receiving 4-NQO showed substantially higher Tigar levels in atypical hyperplasia lesions and ESCC than in normal esophageal epithelium and the Nsun2 levels were significantly correlated with the Tigar levels; in contrast, *Nsun2*^−/−^ mice showed very low levels of Tigar protein in ESCC samples (Supplementary Fig. 5e, f). IHC staining of NSUN2 and TIGAR in human ESCC tissue arrays showed significantly positive correlation (Fig. 1k and Supplementary Table 5). The mRNA stability assays demonstrated that *NSUN2* overexpression significantly increased but knockout significantly decreased the half-life of *TIGAR* mRNA (Fig. 1l and Supplementary Fig. 5g–i). TIGAR may activate the pentose phosphate pathway generating more reductants to protect cancer cells from killing by ROS.^5^ Consistently, we found that *NSUN2* overexpression significantly reduced but knockout significantly increased intracellular ROS levels; however, this effect can be partially rescued by the forced *TIGAR* expression change (Fig. 1m and Supplementary Fig. 5j–l).

In this context, we hypothesized that *NSUN2* overexpression in ESCC may be implicated in radioresistance. Indeed, cells overexpressing *NSUN2* were not or much less sensitive to irradiation killing and had higher abilities of colony formation compared with control cells; in contrast, cells with *NSUN2* knockout had the opposite response to irradiation (Fig. 1n, o and Supplementary Figs. 6a–7h). Furthermore, *NSUN2* overexpression significantly decreased but knockout significantly increased the levels of DNA damages indicated by γ-H2AX or the comet assays (Fig. 1p, q and Supplementary Figs. 8a–9c). All these effects of *NSUN2* expression changes on radiosensitivity of ESCC cells could be rescued by the forced *TIGAR* overexpression or knockdown (Fig. 1n–q and Supplementary Fig. 6a–9c).

Next, we examined the correlation between the levels of NSUN2 and TIGAR in ESCC samples and the radiosensitivity of these tumor-derived xenografts (PDXs) in mice (Supplementary Fig. 10a). PDXs resistant to irradiation had significantly higher NSUN2 and TIGAR levels compared with PDXs sensitive to irradiation (Fig. 1r, s and Supplementary Fig. 10b). In these 19 ESCC tumors for PDXs, the NSUN2 levels were significantly correlated with the TIGAR levels (Supplementary Fig. 10c). We then investigated the associations between NSUN2 and TIGAR levels in ESCC biopsy samples and the tumor sensitivity to adjuvant radiochemotherapy in a patient cohort (*N* = 51). IHC analysis showed that the levels of NSUN2 and TIGAR in ESCC of non-responders were significantly higher than those in ESCC of responders (Fig. 1t, u and Supplementary Fig. 10d). The levels of the two proteins in ESCC were significantly and positively correlated (Supplementary Fig. 10e).

Since the *NSUN2* expression is additionally regulated by the rs10076470 eQTL in ESCC, the effect of *NSUN2* rs10076470 genotype on individual radiochemotherapy vulnerability was further examined in 124 patients receiving adjuvant radiochemotherapy. In 90 patients with the GG genotype, 74.4% (*N* = 67) were responders and only 25.6% (*N* = 23) were non-responders; however, in 34 patients with the GA (*N* = 22) or AA (*N* = 12) genotype, only 47.1% (*N* = 16) were responders but 52.9% (*N* = 18) were non-responders. The odds ratio of radiochemo-resistance for the GA and AA genotypes was 3.28 (95% CI, 1.44−7.47) compared with the GG genotype (*P* = 0.004), suggesting that the *NSUN2* genotype may be a noninvasive marker for evaluating patients’ radiochemo-sensitivity.

In summary, we have characterized the rs10076470 G to A mutation in *NSUN2* that forms a *cis*-eQTL for STAT1, a master TF that is significantly overexpressed in ESCC. The increased *NSUN2* activity due to the genetic variation enhances the expression of many cancer-related genes via mRNA m^5^C methylation, which promotes ESCC progression and radiochemo-resistant phenotype (Fig. 1v). Our study has provided a new insight into the mechanism underlying the differential sensitivity to radiochemotherapy in individuals with ESCC. These results also suggest that the STAT1 inhibitors may be useful for enhancing the radiochemotherapy efficacy in patients with high NSUN2 expression.

## Supplementary information

Supplementary materials

Supplementary Figure 1

Supplementary Figure 2

Supplementary Figure 3

Supplementary Figure 4

Supplementary Figure 5

Supplementary Figure 6

Supplementary Figure 7

Supplementary Figure 8

Supplementary Figure 9

Supplementary Figure 10

## Data Availability

The raw sequencing data of this study have been deposited in the Genome Sequence Archive of Beijing Institute of Genomics, Chinese Academy of Sciences (http://gsa.big.as.cn/) with accession number PRJCA000354, HRA000195 and PRJCA009582. Other source data and reagents are available from the corresponding author upon reasonable request.
