# Supplementary material for: A cis-eQTL in NSUN2 promotes esophageal squamous-cell carcinoma progression and radiochemotherapy resistance by mRNA-m5C methylation
Source: Signal Transduct Target Ther. 2022 Aug 8;7:267. doi: 10.1038/s41392-022-01063-2 (PMC9357702; doi:10.1038/s41392-022-01063-2)
Supplement: Supplementary file 1 — Supplementary materials [file 41392_2022_1063_MOESM1_ESM.docx]

Supplementary Materials for

**A *cis*-eQTL in *NSUN2* promotes esophageal squamous-cell carcinoma progression and radiochemotherapy resistance by mRNA-m^5^C methylation**

Xiangjie Niu^1^, Linna Peng^1^, Weiling Liu^1^, Chuanwang Miao^1^, Xinjie Chen^1^, Jiahui Chu^1^, Xinyu Yang^1^, Wen Tan^1^, Chen Wu^1,2,3^, Dongxin Lin^1,3^

Correspondence to: Chen Wu (chenwu@cicams.ac.cn), Dongxin Lin ([lindx@cicams.ac.cn](mailto:lindx@cicams.ac.cn)) or Wen Tan (tanwen@cicams.ac.cn)

**This PDF file includes:**

Materials and Methods

Figures. S1 to S10

Tables. S1 to S8

**Materials and Methods**

**Analysis of eQTLs**

The identification of eQTLs has been described in our previous publication.^1^ Briefly, SNPs were called using Freebayes based on the whole-genome sequencing of blood DNA and gene expression levels were measured by RNA sequencing in esophageal tissues from 94 individuals with ESCC. The *cis*-eQTLs were analyzed using linear regression with additive model in the R package Matrix eQTL.^1^ The effect size was defined as the slope coefficient of linear regression. Only SNPs within a ± 100-kilobase window of the transcription start site of a given gene on the same chromosome were tested.

**Study subjects and biospecimens**

The present study recruited 3 groups of subjects and collected tissue or blood samples. A panel of 197 formaldehyde-fixed and paraffin-embedded tissues samples for making tissue arrays (obtained between 2015 and 2016) were enrolled from Linzhou Cancer Hospital, Henan Province, China, and the patients’ characteristics have been described previously.^2,3^ *NSUN2* mRNA levels in ESCC tumors were retrieved from two RNA-sequencing databases (N = 154) reported in our previous studies.^4,5^ Among these samples, only 143 had DNA samples available for *NSUN2* genotyping. To increase the statistical power, we collected additional 27 ESCC samples from Linzhou Cancer Hospital making the sample size to 170. We also recruited 140 ESCC patients received the same adjuvant radiochemotherapy at the Cancer Hospital, Chinese Academy of Medical Sciences (CAMS) between 2015 and 2017. Among them, 51 patients had their tumor biopsy samples, obtained before radiochemotherapy, available for IHC analysis. The relevant clinical characteristics of these patients were obtained from patient’s medical records (Supplementary Table 6). The response of ESCC to radiochemotherapy was evaluated according to the RECIST guidelines for solid tumors^6^: complete response (CR) and partial response (PR) were defined as sensitive while stable disease (SD) and progressive disease (PD) were defined as resistant. Written informed consents were obtained from all subjects and this study was approved by CAMS Cancer Hospital.

**Cell lines and cell culture**

Human ESCC cell lines KYSE150 and KYSE450 were kind gifts from Dr. Y. Shimada of Hyogo College of Medicine, Japan. All cell lines were authenticated by DNA finger printing analysis and tested to be free of mycoplasma infection. Cells were cultured in RPMI 1640 medium supplemented with 10% fetal bovine serum (FBS) at 37°C in a humidified incubator with 5% CO_2_.

**RNA sequencing and data analysis**

Total RNA in cell lines was extracted as described above and subjected to sequencing. RNA-sequencing data were mapped to the GRCh38 human genome by HISAT2 (version 2.1.0). The gene expression matrix of raw read counts after annotation by HTSeq (version 0.6.1p1) was processed using DESeq2 (version 1.22.2). The expression profiles were normalized by the Transcripts Per Kilobase of exon model per Million mapped reads (TPM) method and log2 transformed. Gene enrichment analysis was performed on normalized RNA-sequencing data using Metascape (http://metascape.org/gp/index.html#/main/step1).

***NSUN2* genotyping and sanger DNA sequencing**

DNA was extracted from cell lines, ESCC tissues or blood samples using HiPure Universal DNA Kit (3018-03, Magen). The *NSUN2* rs10076470 G/A genotypes were identified by PCR and restriction fragment length polymorphism with *Bsu*36I (R054S, New England Biolabs). The PCR primers were shown in Supplementary Table 7. For validation of *NSUN2* genotyping, PCR products for each genotype on agarose gel were isolated, purified, and subject to Sanger sequencing.

**Plasmids and small interfering RNA transfection**

*NSUN2* expression plasmid (#61117-1) was purchased from Genechem. Small interfering RNAs (siRNAs) targeting TIGAR (#1, gugaucucaugaggacaaa; #2, ccuacaggaucaucuaaau) and STAT1 (#1, guggcaaagagugaucaga; #2, gaccaugccuuuggaaagu) were from Genepharma. The control sequence was uucuccgaacgugucacgutt. The transfection of plasmid and siRNAs was performed using Lipofectamine 2000 (#11668019, Invitrogen).

**Establishment of cell lines with target gene expression disturbance**

Lentivirus for stable *NSUN2* overexpression (#29310-3) was purchased from Genechem as viral particles. KYSE150 and KYSE450 cells were infected with the virus and cultured in complete medium for 24 h followed by selection with puromycin (S7417, Selleck). The CRISPR/Cas9 system was used to generate genomic deletion of *NSUN2* in ESCC cell lines. Single-guide RNA (sgRNA) sequences designed to target the genomic sequence of *NSUN2* were cloned into plasmid pUC19-U6-sgRNA. The pCAG-Cas9-EGFP and pUC19-U6-sgRNA plasmids were co-transfected into HEK293T cells and the fluorescent cells were sorted via flow cytometry. DNA was extracted from harvested cells and the target fragment was amplified and PCR products were re-annealed to generate hetero-duplexed DNA. Then T7EI assay were carried out to confirm the editing efficiency.^7,8^ Two sgRNAs with high efficiency were selected and cloned into plasmid PB-U6-Bbs1-sgRNA-Neo (Supplementary Table 7). KYSE150 and KYSE450 cells were co-transfected with PB-U6-Bbs1-sgRNA-Neo and PBase and cultured with complete medium for 24 h followed by selection with G418 (S3028, Selleck).

**Cell viability and colony formation assays**

Cells were seeded in 96-well plates and the cell viability was measured at various time points using the Cell Counting Kit (CK04, Dojindo Labs). Each experiment was repeated 3 times and each time had 6 replicates. For colony formation assays, cells in 12-well cell-culture cluster with complete growth medium were fixed with methanol, stained with 0.5% crystal violet (C3886, Sigma-Aldrich) and imaged by G:BOX F3EE gel doc system (Syngene). Colony formation ability was determined by using GeneTools automatic image analysis software. Survival fraction of cells was the ratio of the plating efficiency of treated cells to that of control cells.

**Extreme limiting dilution assays**

Extreme limiting dilution assays were performed as described previously.^3^ KYSE150 and KYSE450 cells (200, 100, 50, 25, 12, and 6 per well) were seeded in 96-well plates and treated with IR (4 Gy) for 7−14 days. The colony number was counted and analyzed using an online software (http://bioinf.wehi.edu.au/software/elda/).

**Real-time quantitative PCR (RT-qPCR)**

Total RNA was extracted from cells using the RNA-Quick Purification Kit (RN001, ES Science). A PrimeScript RT reagent kit (RR037A, Takara) and SYBR Premix Ex Taq II kit (RR820A, Takara) were used for the detection of mRNA level with an ABI 7900HT Real-Time PCR system using the primers shown in Supplementary Table 7. The individual mRNA level was determined relative to *GAPDH* or *ACTB* mRNA level.

**Western blot analysis**

Tissues or cells were lysed with RIPA lysis buffer (R0020, Solarbio) containing PMSF (P0100, Solarbio), phosphatase inhibitor cocktail I (HY-K0021, MCE) and phosphatase inhibitor cocktail II (HY-K0022, MCE). Total protein (20 µg) was subjected to SDS-PAGE and transferred to PVDF membrane (IPVH00010, Millipore). Antibody against TIGAR (sc-166290, Santa Cruz), NSUN2 (20854-1-AP, Proteintech), β-ACTIN (66009-1-Ig, Proteintech), STAT1 (ab234400, abcam) or γ-H2AX (Ser139; ab81299, Abcam) was used. The PVDF membrane was blocked and incubated with the primary antibody overnight at 4°C and then with the secondary antibody at room temperature. The signal was detected with a SuperSignal^TM^ West Pico/Femto Chemiluminescent Substrate kit (34580, Thermo-Fisher) through an Amersham Imager. The protein bands were quantified by gray scanning using ImageJ software.

**Electrophoretic mobility-shift assays**

Nuclear proteins were extracted from KYSE150 cells using Nuclear Protein Extraction Kit (#78833, Thermo-Fisher). The 25-bp double-strand oligonucleotides containing rs10076470 G or rs10076470 A were synthesized and labeled with biotin in 3’ end (Supplementary Table 8). Biotin-labeled oligonucleotides and nuclear extract were incubated for 20 min using the Light Shift Chemiluminescent EMSA kit (#20148, Thermo-Fisher). After electrophoresis at 100 volts for 90 min, samples were transferred onto a positively charged nylon membrane (INYC00010, Millipore) at 380 mA for 30 min. Transferred DNAs were cross-linked to the membrane at 120 mJ/cm^2^ for 90 s and detected using the Chemiluminescent EMSA kit.

**Construction of reporter plasmids**

DNA fragment containing rs10076470 G (2,168 base pairs from the position −1,538 to +630) was PCR amplified from a DNA sample with rs10076470 GG genotype. The PCR product was cloned into the pGL4.10 firefly luciferase expression vector to generate the corresponding promotor reporter plasmid (p-Grs10076470). The plasmid was site-specifically mutated to create its rs10076470 A variant counterpart (p-Ars10076470) using Multi-direct kit (SDM-15, SBS Gnentech). The authenticity of all the constructs was verified by sequencing. All primers used in plasmid construction are shown in (Supplementary Table 7).

**Dual-luciferase reporter assays**

KYSE150 cells (6 x 10^4^) or KYSE450 cells (8 x 10^4^) were seeded in 48-well plates for dual luciferase reporter gene assays. To examine the effects of rs10076470 variants, cells were transfected with allele-different reporter constructs as described above. pRL-SV40 control vector encoding Renilla luciferase was co-transfected for normalizing the luciferase activity, determining with a Dual-Luciferase Reporter System (E1960, Promega).

**Dot blot assays for m^5^C**

Total RNA extracted from ESCC cells was denatured at 95^o^C for 3 min followed by chilling on ice. Two-fold serial dilutions were spotted on a positively charged nylon membrane (INYC00010, Millipore), which was UV crosslinked for 3 min at 120 mJ/cm^2^ and washed with buffer. The membrane was stained with 0.02% methylene blue (HY-14536, MCE) to indicate the total content of input RNA. After being blocked with 5% non-fat milk for 1.5 h, the membrane was incubated with specific m^5^C antibody (ab10805, abcam) overnight at 4°C followed by incubating with horseradish peroxidase (HRP)-conjugated anti-mouse IgG. The membrane was then visualized by a SuperSignal^TM^ West Pico/Femto Chemiluminescent Substrate kit (#34580, Thermo-Fisher) in an Amersham Imager.

**Chromatin immunoprecipitation coupled qPCR (ChIP-qPCR) analysis**

ChIP assays were performed by ChIP assay kit (Cat# 10086, Millipore) according to the manufacturer’s instructions. ESCC cells were treated with IFN-α for 6 h and genomic DNA was extracted from the fixed-chromatin cells and sheared by sonication. Antibody against STAT1 or rabbit IgG (Santa Cruz), as control, was respectively incubated with cross-linked protein/DNA overnight for immunoprecipitation using protein A/G magnetic beads. Purified DNA fragments using DNA Purification Kit (D0033, Beyotime) was analyzed by qPCR. The primers used in ChIP-qPCR are shown in Supplementary Table 7.

**m^5^C-mRNA immunoprecipitation coupled RT-qPCR**

The m^5^C-RNA immunoprecipitation was performed as described previously.^9^ Briefly, total RNA from cells was isolated and mRNA was purified using a Dynabeads mRNA purification kit (#61006, Ambion). Anti-m^5^C antibody (5 µg; ab10805, Abcam) was premixed with 50 µl of magnetic Dynabeads protein G (#10007D, Thermo Fisher) in Ab binding and washing buffer for 30 min. The purified mRNA was then added to the beads and antibody mixture and incubated at 4°C overnight. A part of mRNA (1/10) was used as the input. The bound mRNA was treated with 10 mg/ml proteinase K (P8107, New England Biolabs), followed by clean-up with RNA Clean & Concentrator-5 with DNase I Set (R1013, Zymo). The extracted mRNA was reverse transcribed, and RT-qPCR was performed using the primers specific for the target transcripts (Supplementary Table 7). The relative m^5^C levels in the target transcripts were evaluated with input normalization.

**ROS measurement**

Total intracellular ROS was measured with the non-fluorescent probe 2,7-dichlorofluoresce (DCF) in diacetate (DCFH-DA; S0033S, Beyotime).^10^ Briefly, cells were washed with PBS and incubated with 10 μM DCFH-DA at 37°C for 30 min. The intensity of DCF fluorescence was detected by Cytation5 at excitation 488 nm and emission 525 nm.

**RNA stability assay**

Cells with or without stable *NSUN2* overexpression or knockout were seeded in 6-well plates and treated with 6 μM actinomycin D for 0, 2, 4, 6 and 8 h. Total RNA was extracted from cells and subjected to analyzing the expression level of target genes by RT-qPCR. mRNA half-life was calculated as previously described.^9^

**Single-cell gel electrophoresis assays**

Single-cell gel electrophoresis (comet assay) was performed to examined DNA double-strand breaks as previously described.^3^ Briefly, cells treated with or without 4 Gy irradiation for 2 h were collected and suspended in ice-cold PBS. A total of 10^4^ cells was prepared for each assay using the Comet Assay DNA Damage Detection Kit (KGA240, KeyGEN). The sample was then analyzed by microscopy using a Cytation5 (BioTek) and tail moment was analyzed using the Gene 5 software.

**Animal experiments**

We developed *Nsun2* conditional-knockout mice in esophagus using Cre-LoxP system. *Nsun2*^+/+^, *Nsun2*^+/-^ or *Nsun2*^-/-^ C57BL/6J mice were generated by crossing *Nsun2*^Flox/Flox^ or *Nsun2*^Flox/Wt^ mice (Model Animal Research Center of Nanjing University, China) with EDL2-cre mice (B6.Cg-Tg(ED-L2-cre)267Jkat/Nci, Frederick National Laboratory). *Nsun2* genotypes were verified by PCR with the primers shown in Supplementary Table 7. 4-Nitroquilonine *N*-oxide was used to induce murine ESCC as described previously.^2,9^ At the end of experiments, the mouse esophagus was removed and grossly examined and then fixed in formaldehyde and embedded in paraffin. Mouse ESCC PDX models for examining the radiosensitivity were created as described in our previous study^3^ and in the present study, the number of PDXs increased to 19. For IR treatment, mice were X-irradiated (3.5 Gy/min) at PDX once every other day for 4 times at 6 Gy each time using a MultiRad225 (Faxitron). Tumor size was measured at least every 3 days and mice were sacrificed when tumor in control group reached 2000 mm^3^. The sensitivity of PDXs to radiotherapy was defined by relative tumor volume as defined in previously.^3^ Animal experiments were carried out in compliance with approved protocols and guidelines from the Institutional Animal Care and Use Committee of the Chinese Academy of Medical Sciences.

**Immunohistochemical analysis**

Paraffin-embedded sections and tissue arrays were incubated with antibody against NSUN2 (1:600) or TIGAR (1:200) at 4°C overnight and then detected with the DAB Kit (ZLI-9017, ZSGB-BIO). Immunohistochemical staining was analyzed as previously described.^3^ The labeling score of intensity was estimated as negative (0), weak (1), moderate (2) and strong (3). The extent of staining, defined as the percentage of positive stained cells, was scored as 1 (≤ 10%), 2 (11%−50%), 3 (51%−80%) and 4 (> 80%). Total immunoreactive score (IRS) was obtained by multiplying the staining intensity score and the staining extent score and ranked from 0 to 12.

**Statistical analysis**

We used Chi square test or Fisher’s exact test for any independence test between two categorical variables and Students’ *t*-test or Mann-Whitney test for any independence test between a continuous variable and a binary categorical variable, when there was no covariate to adjust for. Spearman’s rank correlation coefficient was used to measure the correlation between two continuous variables, and correlations were considered signiﬁcant and positive when *P* < 0.05 and r > 0.30. We used the log-rank test in univariate survival analyses. Overall survival time was estimated by the Kaplan-Meier method and the differences were examined by the log-rank test. Hazard ratios (HRs) and their 95% confidence intervals (CIs) were calculated with the Cox proportional hazards model. All statistical tests were two-tailed unless specifically indicated and *P* < 0.05 was considered significant. All the statistical analyses were performed using GraphPad Prism 7.0 software (GraphPad Software, La Jolla, CA, USA).

**Reference**

1. Shabalin, A.A. Matrix eQTL: ultra fast eQTL analysis via large matrix operations. *Bioinformatics* **28**, 1353–1358 (2012).
2. Chu, J. et al. Metabolic remodeling by TIGAR overexpression is a therapeutic target in esophageal squamous-cell carcinoma. *Theranostics* 10, 3488–3502 (2020).
3. Liu, W. et al. VAV2 is required for DNA repair and implicated in cancer radiotherapy resistance. *Signal. Transduct. Target. Ther.* **6**, 322 (2021).
4. Chang, J. et al. Genomic analysis of oesophageal squamous-cell carcinoma identifies alcohol drinking-related mutation signature and genomic alterations. *Nat. Commun.* **8**, 15290 (2017).
5. Zhang, X. et al. Dissecting esophageal squamous-cell carcinoma ecosystem by single-cell transcriptomic analysis. *Nat. Commun.* **12**, 5291 (2021).
6. Eisenhauer, E.A. et al. New response evaluation criteria in solid tumours: revised RECIST guideline (version 1.1). *Eur. J. Cancer* **45**, 228–247 (2009).
7. Koike-Yusa, H., Li, Y., Tan, E.P., Velasco-Herrera Mdel, C. & Yusa, K. Genome-wide recessive genetic screening in mammalian cells with a lentiviral CRISPR-guide RNA library. *Nat. Biotechnol.* 32, 267–273 (2014).
8. Zhou, Y. et al. High-throughput screening of a CRISPR/Cas9 library for functional genomics in human cells. *Nature* **509**, 487–491 (2014).
9. Su, J. et al. NSUN2-mediated RNA 5-methylcytosine promotes esophageal squamous cell carcinoma progression via LIN28B-dependent GRB2 mRNA stabilization. *Oncogene* **40**, 5814–5828 (2021).
10. Liu, M.X. et al. Metabolic reprogramming by PCK1 promotes TCA cataplerosis, oxidative stress and apoptosis in liver cancer cells and suppresses hepatocellular carcinoma. *Oncogene* **37**, 1637–1653 (2018).

**
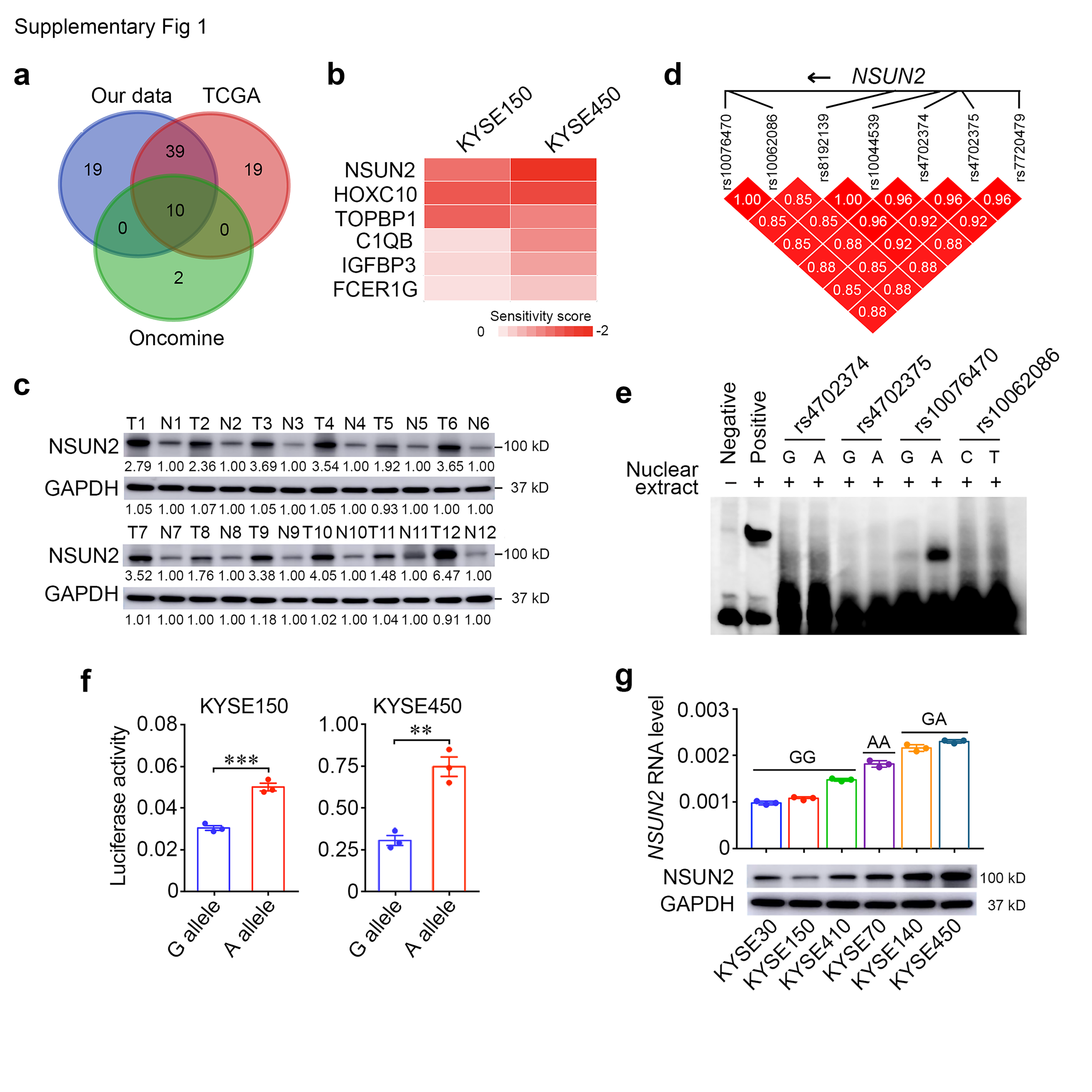
**

**Supplementary Figure 1. Identification of *NSUN2* as an eQTL-target gene in ESCC.** **a** Venn diagram displays 10 genes overlapped among our 94 ESCC dataset, TCGA dataset and oncomine dataset had significantly higher mRNA expression in ESCC tissues than normal tissues (Foldchange > 1.5, *P* < 0.05). **b** Heat map displaying the inhibitory effects of silencing 6 genes on the malignant phenotypes of two ESCC cell lines. Sensitivity scores are from Drive data portal. **c** Western blot analysis of NSUN2 protein levels in 12 paired ESCCs and adjacent normal fresh tissues, showing that most of ESCCs expressed higher NSUN2 than adjacent normal tissues. **d** Linkage disequilibrium (LD) plot by PLINK shows the 7 SNPs are LD (all r^2^ > 0.85). **e** Electrophoretic mobility-shift assays (EMSA) with biotin-labeled probes containing different SNP alleles using KYSE150 cell nuclear extract, showing that a DNA-protein complex was only seen when the DNA fragment containing the rs10076470 A not the rs10076470 G or the DNA fragments containing other SNPs was incubated with the nuclear protein extracted from KYSE150 cells. **f** Reporter gene assays show higher promoter activity of the construct containing the rs10076470 A allele than that containing the rs10076470 G allele in KYSE150 and KYSE450 cells. All constructs were co-transfected with pRL-SV40 plasmid to standardize transfection efficiency. Data are means ± S.E.M. from 3 replicate experiments. **, *P* < 0.01 and ***, *P* < 0.001 of Student’s t-test. **g** The *NSUN2* mRNA (*upper* *panel*) and protein (*lower panel*) levels in 6 ESCC cell lines.


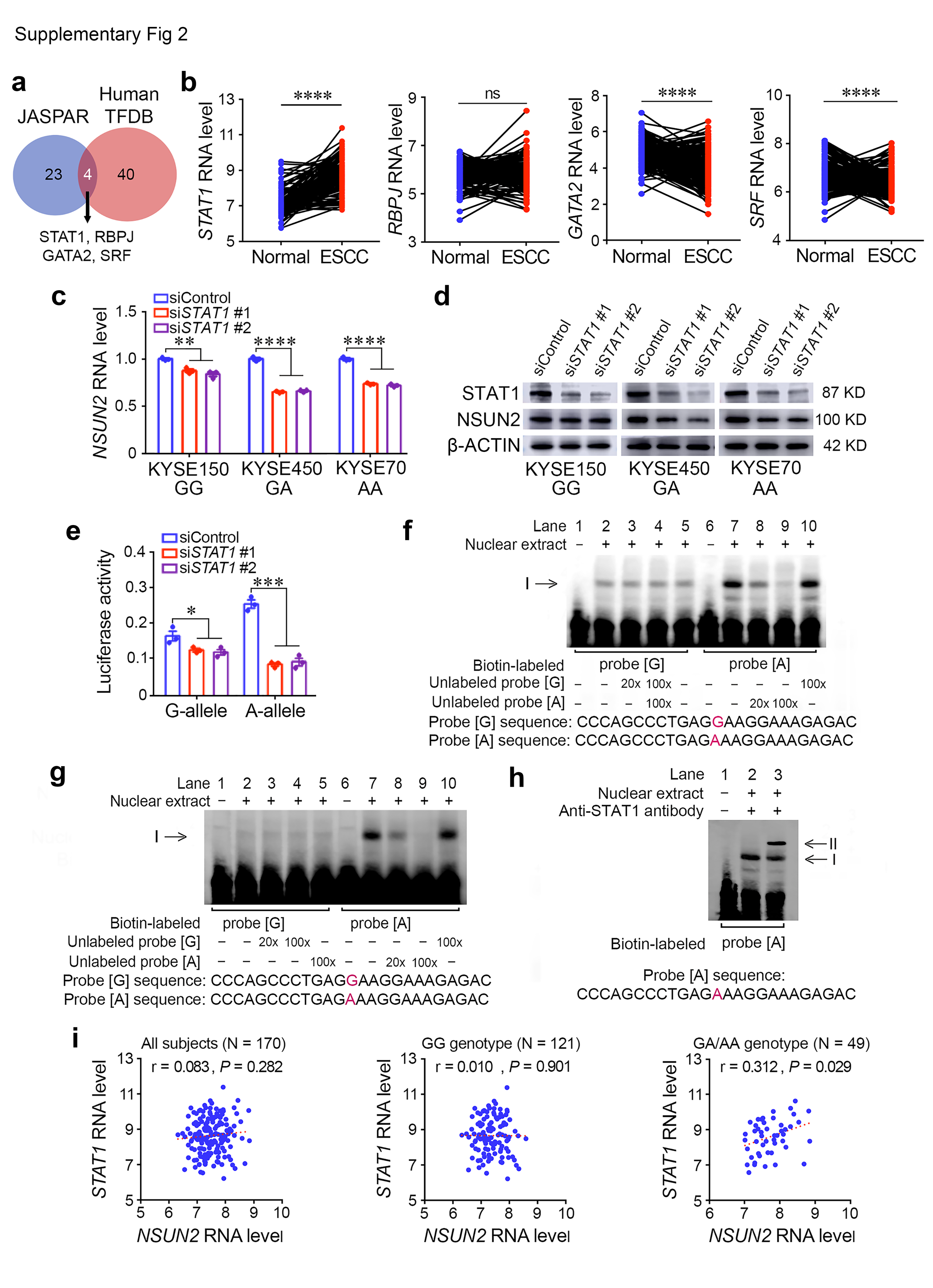


**Supplementary Figure 2. The eQTL *NSUN2* variant expression is regulated by STAT1 in ESCC.** **a** Venn diagram displays 4 transcription factors (TF) overlapped in conducting TF motif analysis of the *NSUN2* rs10076470 A allele using JASPAR and Human TFDB databases. **b** STAT1 mRNA levels in ESCCs are significantly higher than adjacent normal tissues, while *RBPJ* mRNA levels in ESCCs have no significant change between ESCCs and normal samples and *GATA2* and *SRF* mRNA levels in ESCCs are significantly lower than adjacent normal tissues (N = 170). ****, *P* < 0.0001 and ns, not significant of paired Student’s t-test. **c** and **d** The effects of *STAT1* knockdown on *NSUN2* mRNA (**c**) and protein (**d**) levels in ESCC cell lines with different rs10076470 genotype. Shown are fold change relative to siControl from 3 replicate experiments (**c**). **, *P* < 0.01 and ****, *P* < 0.0001 of Student’s *t*-test. **e** The effect of *STAT1* knockdown on luciferase activity of constructs containing the rs10076470 G or rs10076470 A allele in KYSE450 cells. Data are mean ± S.E.M. from 3 replicate experiments. *, *P* < 0.05 and ***, *P* < 0.001 of Student’s t-test. **f** and **g** Electrophoretic mobility-shift assay (EMSA) with biotin-labeled probes containing rs10076470 G or rs10076470 A allele using KYSE150 (**f**) and KYSE450 (**g**) cell nuclear extract. Arrow points a rs10076470 A-specific DNA-protein interaction band, which can be eliminated by the addition of 100-fold excessive unlabeled rs10076470 A probe but not rs10076470 G probe. **h** Super-shift EMSA competition assay with STAT1 antibody and KYSE450 cell nuclear extract. I points the rs10076470 A-specific band and II points a super-shifted band by STAT1. **i** Spearman correlations between *STAT1* RNA and *NSUN2* RNA levels in all samples (*left panel*), samples with the *NSUN2* GG (meddle panel) or samples with the *NSUN2* GA or AA genotype (*right panel*).


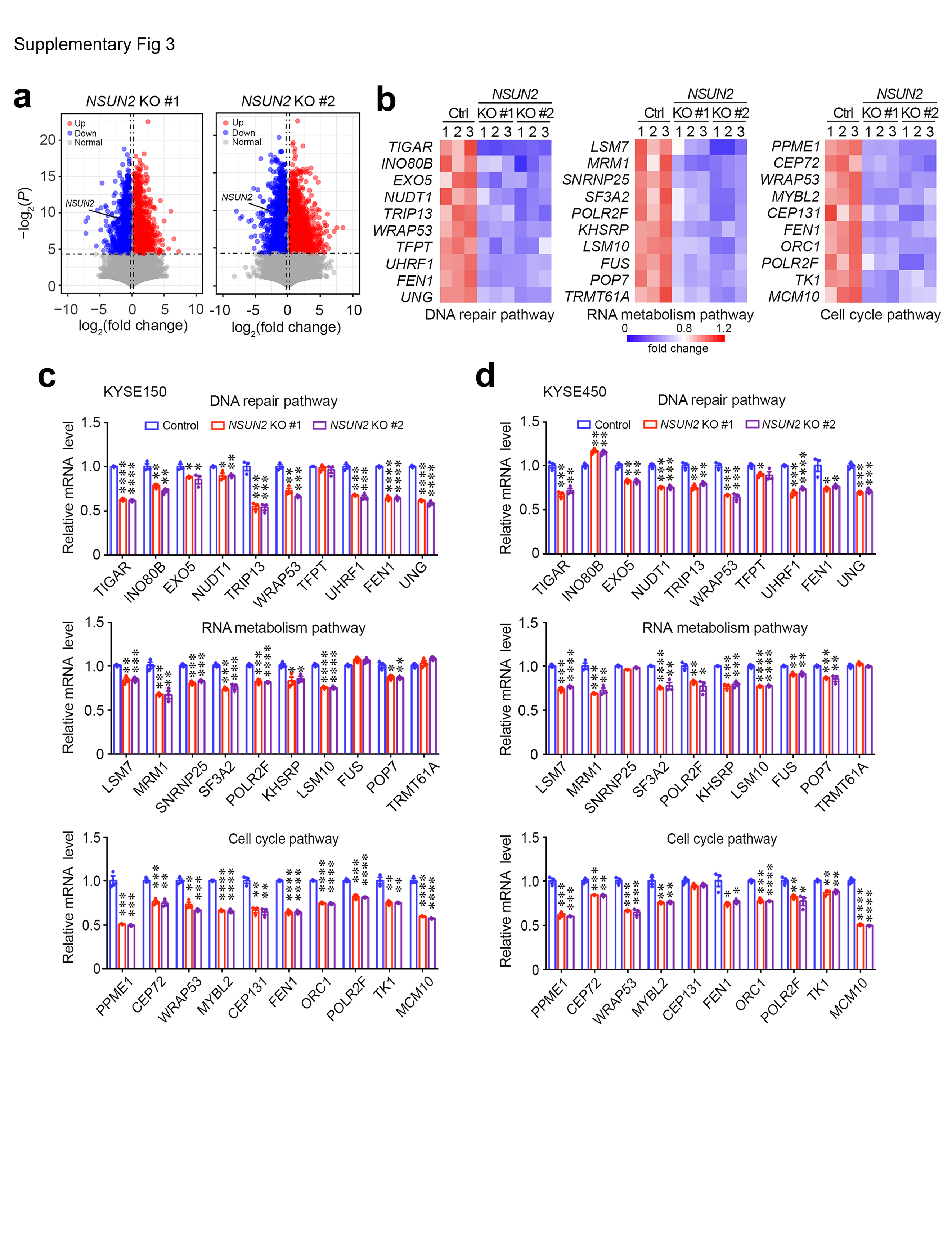


**Supplementary Fiure 3. NSUN2 upregulates transcripts of ESCC-associated genes.** **a** Volcano plot displays upregulated (fold change > 1.2; *P* < 0.05) or downregulated genes (fold change < 0.8; *P* < 0.05) in KYSE150 cells with *NSUN2* knockout (KO) #1 and KO #2. Data are from 3 replicate experiments. **b** Heat map shows the top 10 differentially expressed genes in DNA repair pathway, RNA metabolism pathway and cell cycle pathway in KYSE150 cells with *NSUN2* KO. **c** and **d** Levels of top 10 transcripts in DNA repair, RNA metabolism, and cell cycle pathway in KYSE150 (**c**) and KYSE450 cells (**d**). Data are mean ± S.E.M. from 3 replicate experiments. *, *P* < 0.05; **, *P* < 0.01; ***, *P* < 0.001 and ****, *P* < 0.0001 of Student’s *t*-test.


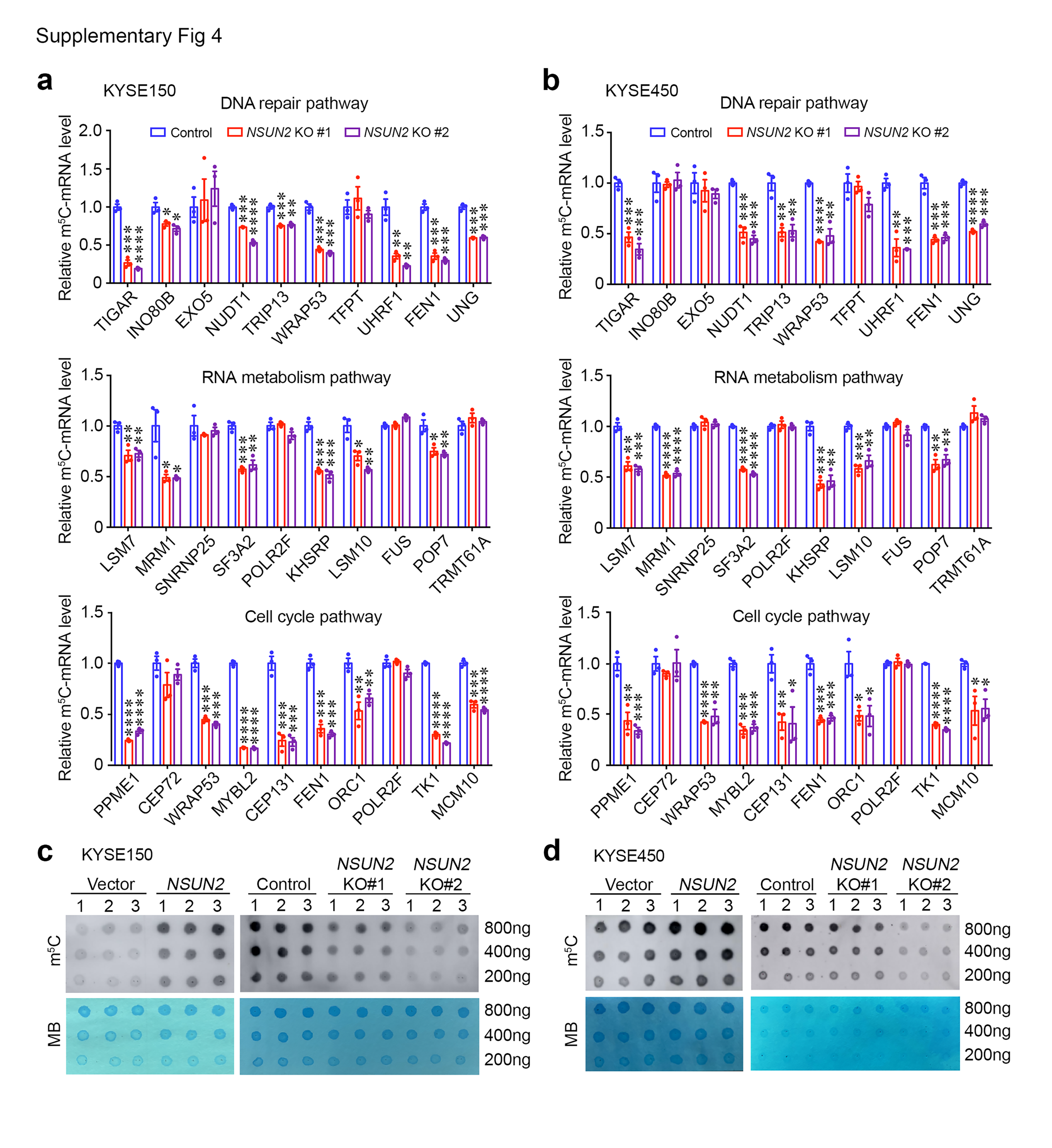


**Supplementary Figure 4. NSUN2 upregulates transcripts of ESCC-associated genes by mRNA-m^5^C modification. a** and **b** The levels of m^5^C-mRNA of top 10 transcripts in DNA repair, RNA metabolism, and cell cycle pathway in KYSE150 (**a**) annd KYSE450 (**b**) cells. Data are mean ± S.E.M. from 3 replicate experiments. *, *P* < 0.05; **, *P* < 0.01; ***, *P* < 0.001 and ****, *P* < 0.0001 of Student’s *t*-test. **c** and **d** The m^5^C level of total RNA in KYSE150 cells (**c**) or KYSE450 cells (**d**) with NSUN2 overexpression or NSUN2 knockout was indicated by m^5^C dot blot. Corresponding RNAs were loaded equally by a 2-fold serial dilution with 800 ng, 400 ng and 200 ng.


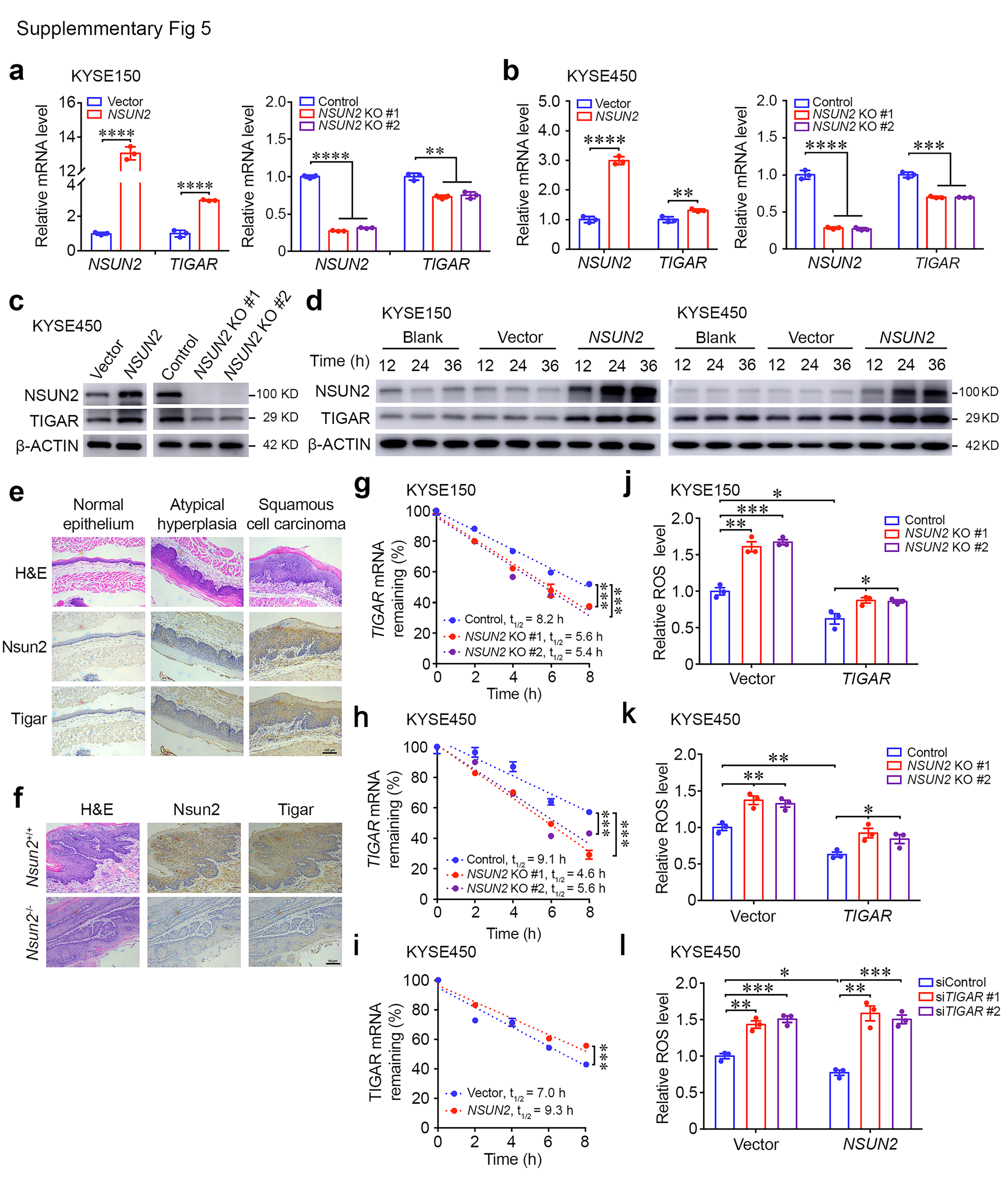


**Supplementary Figure 5. NSUN2 upregulates *TIGAR* expression via enhancing *TIGAR* mRNA stability by m^5^C modification. a** and **b** The *NSUN2* mRNA and *TIGAR* mRNA levels in KYSE150 (**a**) and (**b**) cells with *NSUN2* overexpression (OE) or knockout (KO). The mRNA levels determined by RT-qPCR are mean ± S.E.M. from 3 independent experiments and each had 3 replications. **, *P* < 0.01 and ****, *P* < 0.0001 of Student’s *t*-test. **c** Western blot analysis of NSUN2 and TIGAR protein levels in KYSE450 cells with *NSUN2* OE or KO. **d** Western blot analysis of TIGAR protein level in KYSE150 and KYSE450 cells with transient *NSUN2* OE, indicating that TIGAR levels were elevated in KYSE150 and KYSE450 cells with *NSUN2* OE in a time-dependent manner. **e** Immunohistochemical (IHC) staining of Nsun2 and Tigar in esophageal tissue samples from *Nsun2*^+/+^ mice treated with 4-NQO, showing that higher Tigar levels in atypical hyperplasia lesions and ESCCs than in normal esophageal epithelium and the Nsun2 levels were positively and significantly correlated with the Tigar levels. **f** IHC staining of Nsun2 and Tigar in 4-NQO induced mice ESCC, showing that Tigar protein was present in *Nsun2*^+/+^ mice but not *Nsun2*^-/-^ mice. **g**–**i** The effect of *NSUN2* KO (**g** and **h**) or OE (**i**) on the TIGAR mRNA stability in KYSE150 (**g**) and KYSE450 (**h** and **i**) cells determined by RT-qPCR at indicated time points after treatment with 6 μM actinomycin D. Data are mean ± S.E.M. In most data points, the error bars are within the symbols. ***, *P* < 0.001 of Student’s *t*-test. **j** and **k** *NSUN2* KO significantly increased ROS formation in KYSE150 (**j**) and KYSE450 (**k**) cells, which could be rescued by overexpressing *TIGAR*. **l** *NSUN2* OE significantly inhibited ROS production in KYSE450 cells, which could be rescued by silencing *TIGAR*. Data in **j**–**l** are mean ± S.E.M. from 3 experiments and each had 3 replications. *, *P* < 0.05; **, *P* < 0.01 and ***, *P* < 0.001 of Student’s *t*-test.


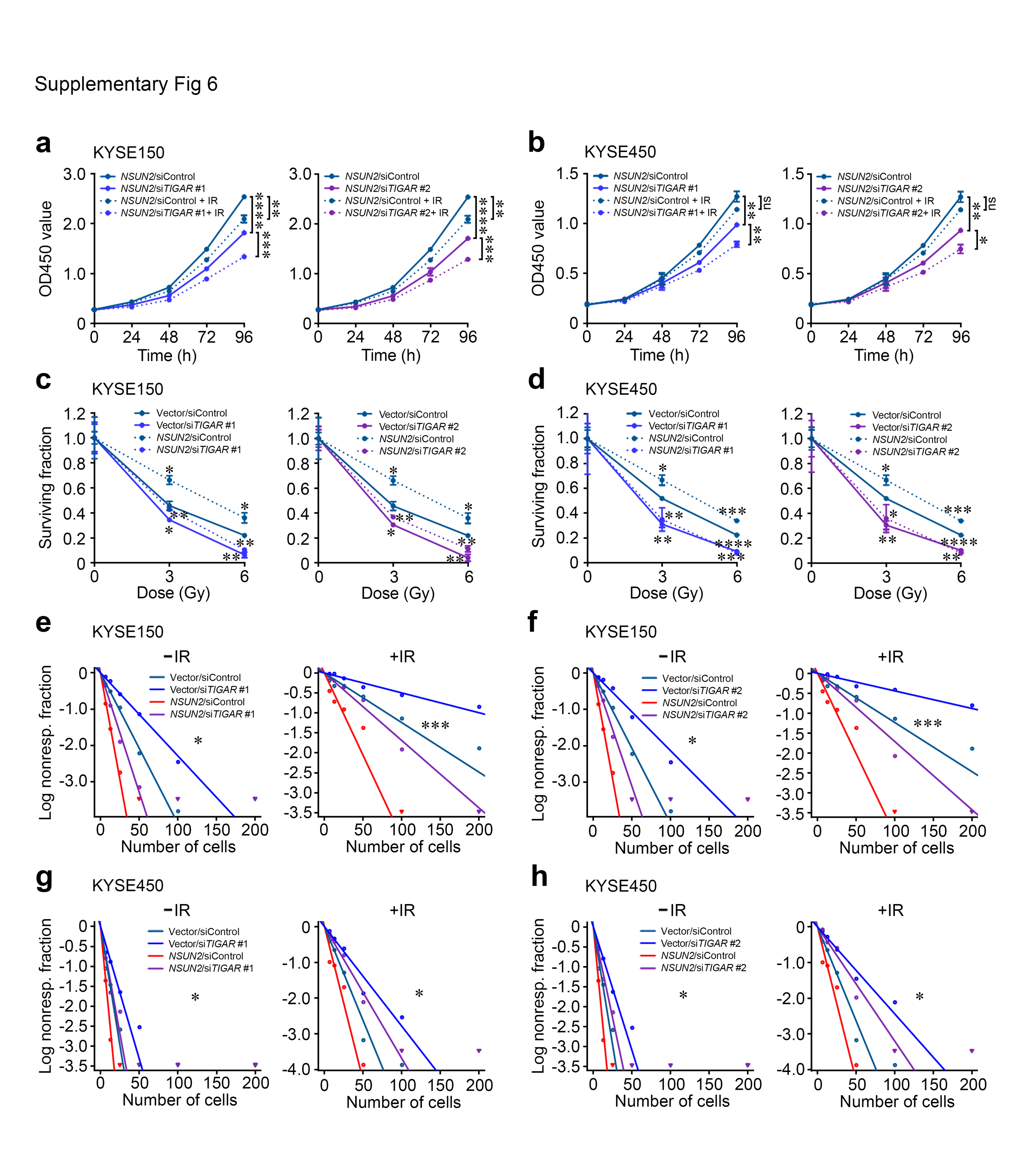


**Supplementary Figure 6. *NSUN2* overexpression promotes ESCC cellls radioresistance, which could be rescued by forced *TIGAR* knockdown. a** and **b** The effect of *NSUN2* overexpression (OE) on proliferation of KYSE150 (**a**) and KYSE450 (**b**) cells with or without ionizing radiation (IR, 4 Gy) and *TIGAR* knockdown. **c** and **d** The effect of *NSUN2* OE on colony formation ability of KYSE150 (**c**) and KYSE450 (**d**) cells with or without ionizing radiation (IR, 4 Gy) and *TIGAR* knockdown. **e**–**h** Extreme limiting dilution assays (ELDA) show survival fractions in KYSE150 (**e** and **f**) and KYSEE450 (**g** and **h**) cells with *NSUN2* OE caused by IR (4 Gy) and *TIGAR* knockdown. Shown in **a**–**d** are mean ± S.E.M. from 3 experiments and each had 3 replications. In some data points, the error bars are within the symbols. *, *P* < 0.05; **, *P* < 0.01; ***, *P* < 0.001; ****, *P* < 0.0001 and ns, not significant of Student’s *t*-test (**a**–**d**) and ELDA analysis program (**e**–**h**).


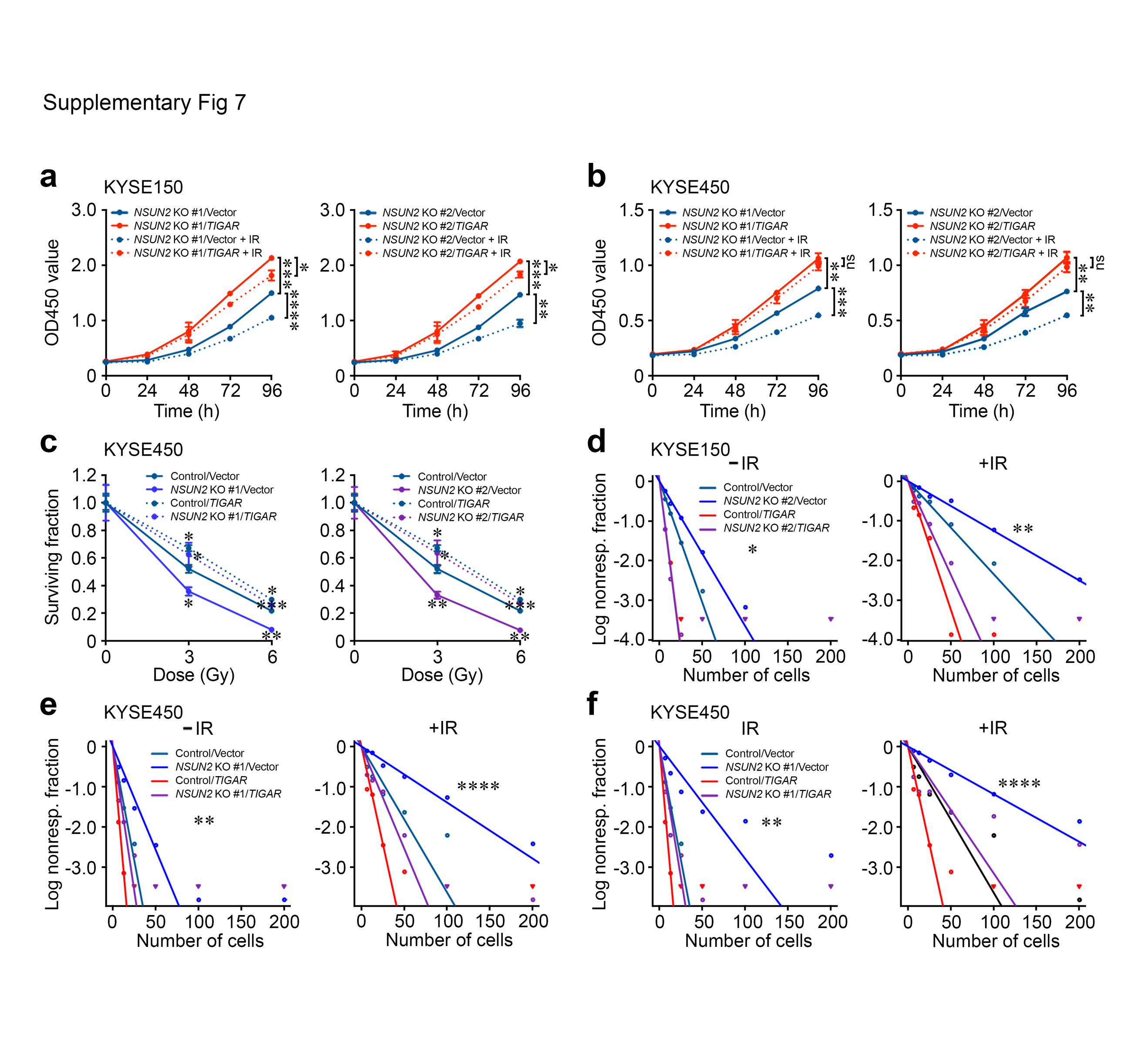


**Supplementary Figure 7. *NSUN2* knockout promotes ESCC cellls radiosensitive, which could be rescued by forced *TIGAR* overexpression. a** and **b** The effect of *NSUN2* knockout (KO) on proliferation of KYSE150 (**a**) and KYSE450 (**b**) cells with or without ionizing radiation (IR, 4 Gy) and *TIGAR* overexpression (OE). **c** The effect of *NSUN2* KO on colony formation ability of KYSE450 cells with or without ionizing radiation (IR, 4 Gy) and *TIGAR* OE. **d**–**f** Extreme limiting dilution assays (ELDA) show survival fractions in KYSE150 (**d**) or KYSE450 (**e** and **f**) cells with *NSUN2* KO caused by IR (4 Gy) and *TIGAR* OE. Shown in **a**–**c** are mean ± S.E.M. from 3 experiments and each had 3 replications. In some data points, the error bars are within the symbols. *, *P* < 0.05; **, *P* < 0.01; ***, *P* < 0.001; ****, *P* < 0.0001 and ns, not significant of Student’s *t*-test (**a**–**c**) and ELDA analysis program (**d**–**f**).


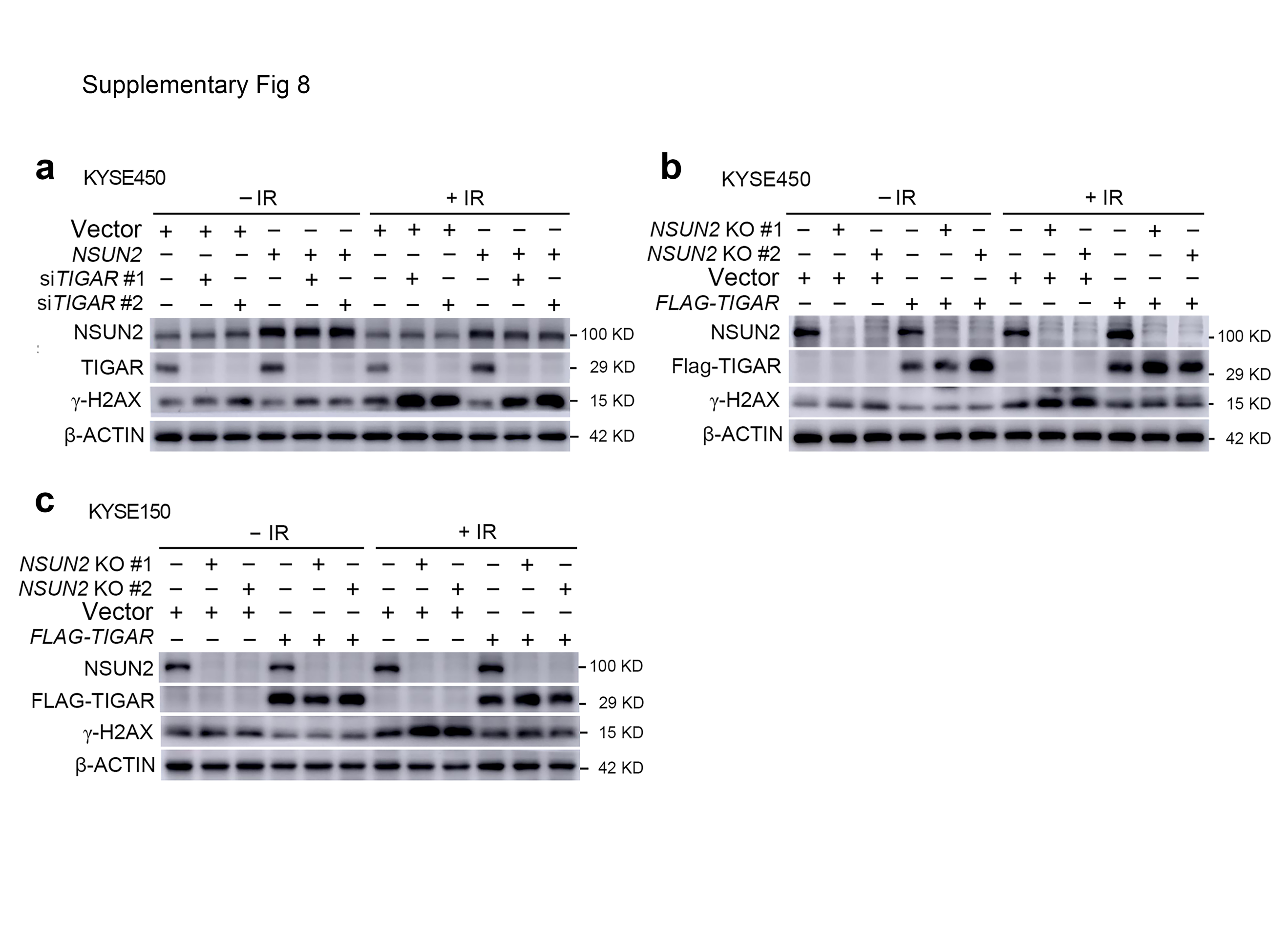


**Supplementary Figure 8. *NSUN2* overexpression significantly decreased but *NSUN2* knockout significantly increased the γ-H2AX levels, which could be rescued by forced *TIGAR* expression alteration.** **a** The effect of *NSUN2* overexpression (OE) on DNA double-strand breaks detected by γ-H2AX in KYSE450 cells with or without IR (4 Gy) and *TIGAR* knockdown. **b** and **c** The effect of *NSUN2* knockout on DNA double-strand breaks detected by γ-H2AX in KYSE450 (**b**) and KYSE150 (**c**) cells with or without IR (4 Gy) and *TIGAR* OE.


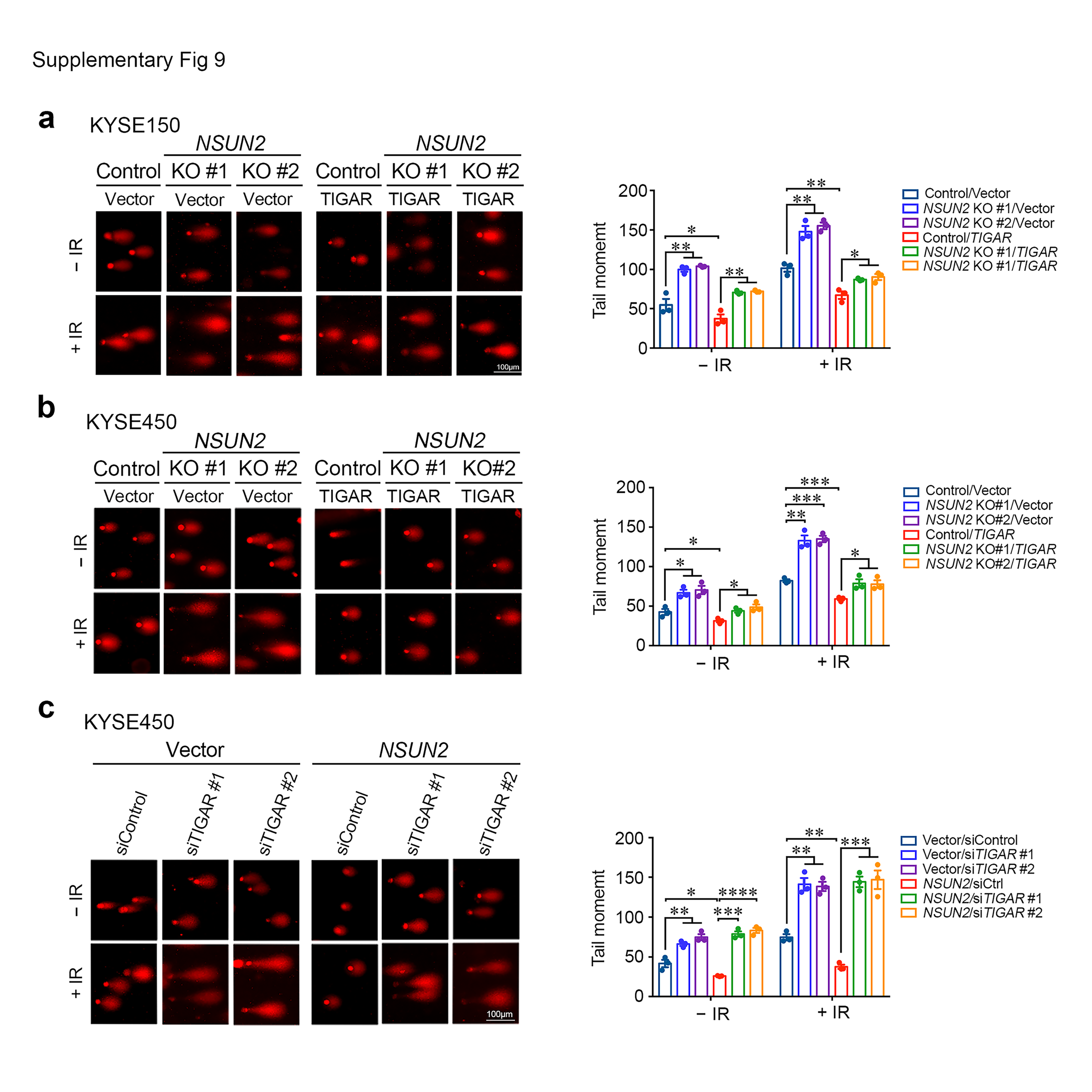


**Supplementary Figure 9. *NSUN2* overexpression significantly decreased but *NSUN2* knockout significantly increased spontaneous and IR-caused DNA damages, which could be rescued by forced *TIGAR* expression alteration. a** and **b** The effect of *NSUN2* knockout (KO) on DNA double-strand breaks detected by comet assays in KYSE150 (**a**) and KYSE450 (**b**) cells with or without IR (4 Gy) and *TIGAR* overexpression (OE). **c** The effect of *NSUN2* OE on DNA double-strand breaks detected by comet assays in KYSE450 cells with or without IR (4 Gy) and *TIGAR* knockdown. *Left* *panels* show fluorescence images of comet assays (scale bars, 100 μm) and *right panels* show the statistics. Data in **a**–**c** are mean ± S.E.M. from 3 replicates and 3 fields were randomly selected from each experiment. *, *P* < 0.05; **, *P* < 0.01; ***, *P* < 0.001 and ****, *P* < 0.0001 of Student’s *t*-test.


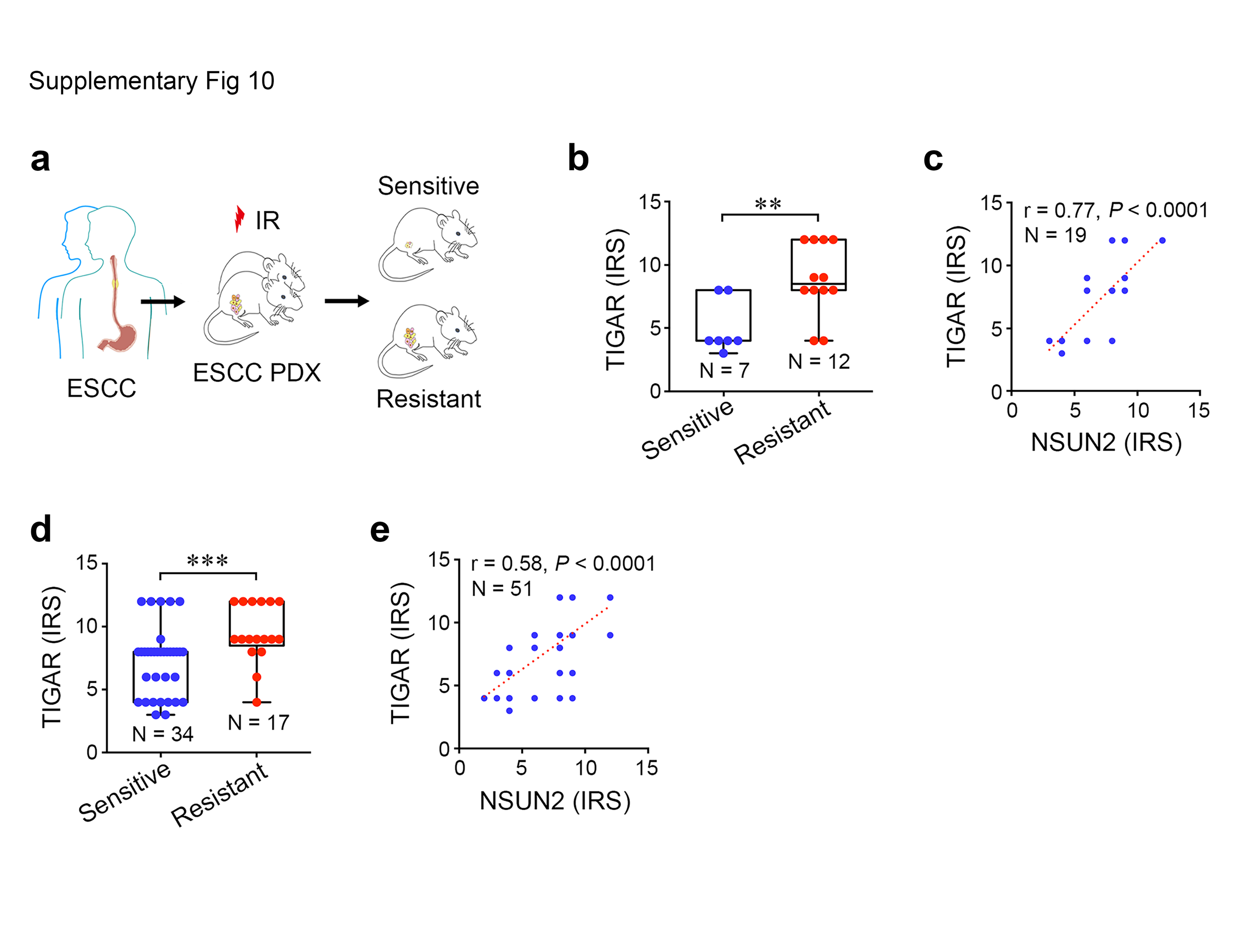


**Supplementary Figure 10. Relevance of aberrant NSUN2 and TIGAR expression to clinical ESCC radiochemotherapy. a** Schematic diagram for the construction of patient-derived xenograft (PDX) of ESCC and irradiation (IR) treatment. **b** Box and bar plots comparing the TIGAR protein levels in PDXs with differential radiosensitivity. **, *P* < 0.01 of Mann-Whitney test. **c** Spearman correlation between the NSUN2 levels and TIGAR levels in PDXs. **d** Box and bar plots comparing the TIGAR protein levels in ESCC biopsy specimens with differential sensitivity to adjuvant radiochemotherapy. ***, *P* < 0.001 of Mann-Whitney test. **e** Spearman correlation between the NSUN2 levels and TIGAR levels in ESCC biopsy specimens. IRS, immunoreactive score.

**Supplementary Table 1. 10 differentially expressed genes in ESCC from 3 datasets**

| Gene | Our data (N = 94) | | TCGA (N = 90) | | Oncomine (N = 51) | |
| --- | --- | --- | --- | --- | --- | --- |
|  | Fold change | *P* value | Fold change | *P* value | Fold change | *P* value |
| NSUN2 | 1.88 | 3.17E-30 | 1.87 | 5.07E-08 | 1.61 | 7.37E-13 |
| IGFBP3 | 4.67 | 1.39E-24 | 2.69 | 3.33E-03 | 3.32 | 2.37E-13 |
| FIGNL1 | 1.94 | 1.75E-19 | 2.88 | 1.30E-05 | 2.06 | 1.23E-13 |
| HOXC10 | 45.39 | 5.17E-39 | 41.76 | 3.47E-07 | 1.66 | 1.91E-13 |
| C16orf75 | 3.55 | 1.89E-25 | 6.67 | 1.28E-04 | 1.67 | 4.62E-14 |
| TYROBP | 1.73 | 4.94E-08 | 2.70 | 1.08E-03 | 1.67 | 1.29E-07 |
| TOPBP1 | 2.13 | 7.20E-36 | 2.52 | 3.46E-09 | 2.10 | 4.76E-18 |
| DSC3 | 3.54 | 5.71E-06 | 119.13 | 7.96E-04 | 1.52 | 2.00E-03 |
| C1QB | 1.74 | 2.62E-06 | 2.56 | 1.66E-02 | 1.82 | 1.29E-07 |
| FCER1G | 2.19 | 4.38E-13 | 3.52 | 2.74E-04 | 1.60 | 1.14E-09 |

**Supplementary Table 2. Clinical characteristics and NSUN2 immunoreactive score of 197 patients with ESCC in this study**

| Patient ID | Age (year) | Sex^a^ | Smoker^b^ | Drinker^c^ | TNM stage^d^ | T stage | N stage | M stage | Survival status | Survival time (month) | NSUN2 in normal tissue | | | NSUN2 in ESCC | | |
| --- | --- | --- | --- | --- | --- | --- | --- | --- | --- | --- | --- | --- | --- | --- | --- | --- |
|  |  |  |  |  |  |  |  |  |  |  | Positive | Intensity | IRS^e^ | Positive | Intensity | IRS^e^ |
| 150115 | 51 | M | Yes | No | II | 3 | 0 | 0 | Deceased | 33.6 | 1 | 3 | 3 | 2 | 2 | 4 |
| 150146 | 52 | M | Yes | Yes | II | 3 | 0 | 0 | Deceased | 25.1 | 2 | 2 | 4 | 2 | 2 | 4 |
| 150159 | 63 | M | Yes | No | II | 2 | 0 | 0 | Alive | 45.8 | 1 | 2 | 2 | 3 | 3 | 9 |
| 150201 | 63 | M | No | No | II | 3 | 0 | 0 | Alive | 45.7 | 1 | 2 | 2 | 1 | 3 | 3 |
| 150203 | 61 | M | Yes | No | I | 3 | 0 | 0 | Deceased | 38.6 | 1 | 3 | 3 | 2 | 3 | 6 |
| 150208 | 60 | M | Yes | Yes | III | 2 | 2 | 0 | Deceased | 17.6 | 2 | 2 | 4 | 2 | 3 | 6 |
| 150219 | 68 | M | No | No | III | 3 | 3 | 0 | Deceased | 21.6 | 2 | 3 | 6 | 3 | 3 | 9 |
| 150228 | 61 | M | Yes | Yes | I | 1 | 0 | 0 | Alive | 45.4 | 1 | 3 | 3 | 2 | 3 | 6 |
| 150229 | 67 | F | No | No | II | 2 | 0 | 0 | Alive | 45.3 | 1 | 3 | 3 | 1 | 4 | 4 |
| 150234 | 61 | M | Yes | No | II | 2 | 0 | 0 | Alive | 44.8 | 2 | 2 | 4 | 2 | 3 | 6 |
| 150236 | 57 | M | Yes | Yes | I | 1 | 0 | 0 | Alive | 44.8 | 2 | 2 | 4 | 2 | 3 | 6 |
| 150241 | 62 | M | Yes | Yes | III | 3 | 2 | 0 | Deceased | 10.0 | 2 | 3 | 6 | 2 | 3 | 6 |
| 150302 | 62 | M | No | No | III | 3 | 1 | 0 | Deceased | 20.8 | 2 | 3 | 6 | 2 | 4 | 8 |
| 150304 | 58 | M | Yes | No | II | 2 | 1 | 0 | Deceased | 23.9 | 2 | 3 | 6 | 3 | 3 | 9 |
| 150312 | 62 | M | Yes | Yes | II | 3 | 0 | 0 | Alive | 44.9 | 2 | 3 | 6 | 3 | 3 | 9 |
| 150313 | 69 | M | Yes | No | II | 3 | 0 | 0 | Alive | 44.9 | 2 | 2 | 4 | 3 | 2 | 6 |
| 150319 | 77 | M | Yes | No | II | 2 | 1 | 0 | Deceased | 3.4 | 2 | 3 | 6 | 3 | 3 | 9 |
| 150323 | 66 | M | No | No | III | 3 | 1 | 0 | Deceased | 31.9 | 2 | 2 | 4 | 2 | 3 | 6 |
| 150324 | 51 | M | Yes | Yes | II | 2 | 0 | 0 | Alive | 44.8 | 2 | 2 | 4 | 3 | 2 | 6 |
| 150331 | 69 | M | Yes | Yes | II | 2 | 1 | 0 | Deceased | 19.6 | 2 | 3 | 6 | 2 | 4 | 8 |
| 150333 | 69 | M | Yes | No | II | 3 | 0 | 0 | Deceased | 27.6 | 2 | 2 | 4 | 2 | 4 | 8 |
| 150334 | 56 | M | Yes | No | IV | 3 | 2 | 1 | Deceased | 30.8 | 2 | 2 | 4 | 2 | 3 | 6 |
| 150348 | 74 | M | Yes | Yes | I | 1 | 0 | 0 | Deceased | 33.7 | 1 | 1 | 1 | 1 | 4 | 4 |
| 150355 | 58 | M | Yes | Yes | III | 3 | 1 | 0 | Deceased | 0.5 | 1 | 2 | 2 | 2 | 3 | 6 |
| 150374 | 57 | F | No | No | II | 2 | 1 | 0 | Alive | 44.4 | 1 | 3 | 3 | 2 | 3 | 6 |
| 150378 | 60 | M | No | No | I | 1 | 0 | 0 | Alive | 44.4 | 1 | 3 | 3 | 2 | 2 | 4 |
| 150385 | 71 | F | No | No | III | 3 | 2 | 0 | Alive | 44.3 | 2 | 2 | 4 | 2 | 3 | 6 |
| 150388 | 65 | M | Yes | Yes | II | 3 | 0 | 0 | Deceased | 7.9 | 2 | 3 | 6 | 3 | 3 | 9 |
| 150395 | 62 | F | No | No | III | 3 | 1 | 0 | Deceased | 11.9 | 1 | 1 | 1 | 2 | 3 | 6 |
| 150411 | 50 | M | Yes | Yes | II | 3 | 0 | 0 | Alive | 43.8 | 3 | 2 | 6 | 3 | 3 | 9 |
| 150414 | 65 | M | Yes | Yes | II | 2 | 0 | 0 | Alive | 43.8 | 2 | 3 | 6 | 1 | 3 | 3 |
| 150418 | 65 | M | Yes | Yes | III | 3 | 2 | 0 | Deceased | 6.6 | 2 | 3 | 6 | 2 | 4 | 8 |
| 150423 | 62 | M | Yes | Yes | II | 3 | 0 | 0 | Alive | 43.7 | 2 | 3 | 6 | 3 | 3 | 9 |
| 150429 | 52 | M | Yes | Yes | III | 3 | 3 | 0 | Deceased | 7.3 | 3 | 3 | 9 | 2 | 4 | 8 |
| 150431 | 59 | M | Yes | Yes | II | 2 | 0 | 0 | Alive | 43.6 | 2 | 3 | 6 | 3 | 2 | 6 |
| 150432 | 57 | F | No | No | II | 3 | 0 | 0 | Alive | 43.5 | 2 | 2 | 4 | 2 | 3 | 6 |
| 150454 | 62 | M | No | No | III | 3 | 2 | 0 | Alive | 42.3 | 2 | 3 | 6 | 3 | 3 | 9 |
| 150455 | 65 | M | No | Yes | III | 4 | 1 | 0 | Deceased | 4.9 | 1 | 2 | 2 | 1 | 4 | 4 |
| 150458 | 68 | F | No | No | II | 3 | 0 | 0 | Deceased | 34.3 | 1 | 3 | 3 | 1 | 4 | 4 |
| 150463 | 54 | M | Yes | Yes | III | 2 | 2 | 0 | Alive | 42.2 | 2 | 1 | 2 | 2 | 3 | 6 |
| 150468 | 64 | M | Yes | Yes | IV | 3 | 2 | 1 | Deceased | 32.1 | 2 | 3 | 6 | 2 | 4 | 8 |
| 150471 | 61 | M | Yes | No | I | 1 | 0 | 0 | Deceased | 26.0 | 1 | 3 | 3 | 2 | 3 | 6 |
| 150472 | 74 | M | No | No | III | 3 | 1 | 0 | Deceased | 27.0 | 2 | 3 | 6 | 1 | 4 | 4 |
| 150476 | 60 | M | No | No | II | 2 | 0 | 0 | Alive | 42.0 | 1 | 3 | 3 | 2 | 3 | 6 |
| 150481 | 65 | F | No | No | IV | 3 | 2 | 1 | Alive | 41.8 | 1 | 3 | 3 | 2 | 3 | 6 |
| 150502 | 52 | M | Yes | Yes | III | 3 | 1 | 0 | Deceased | 7.5 | 2 | 3 | 6 | 3 | 3 | 9 |
| 150503 | 70 | M | Yes | Yes | III | 4 | 1 | 0 | Deceased | 6.5 | 2 | 3 | 6 | 3 | 3 | 9 |
| 150506 | 73 | M | Yes | No | IV | 3 | 2 | 1 | Deceased | 4.4 | 2 | 3 | 6 | 3 | 3 | 9 |
| 150510 | 55 | M | Yes | Yes | II | 3 | 0 | 0 | Alive | 41.8 | 1 | 2 | 2 | 1 | 4 | 4 |
| 150513 | 61 | M | Yes | No | II | 3 | 0 | 0 | Deceased | 3.5 | 2 | 3 | 6 | 3 | 4 | 12 |
| 150526 | 60 | M | Yes | Yes | II | 2 | 0 | 0 | Alive | 41.6 | 2 | 3 | 6 | 3 | 4 | 12 |
| 150527 | 67 | F | No | No | II | 2 | 1 | 0 | Deceased | 7.2 | 2 | 3 | 6 | 2 | 4 | 8 |
| 150528 | 68 | M | Yes | Yes | II | 3 | 0 | 0 | Alive | 41.6 | 2 | 3 | 6 | 3 | 3 | 9 |
| 150531 | 57 | M | Yes | Yes | III | 2 | 2 | 0 | Alive | 41.8 | 1 | 3 | 3 | 1 | 4 | 4 |
| 150543 | 74 | M | Yes | Yes | III | 3 | 1 | 0 | Deceased | 14.1 | 2 | 2 | 4 | 3 | 3 | 9 |
| 150544 | 65 | M | Yes | Yes | I | 2 | 0 | 0 | Deceased | 29.8 | 1 | 1 | 1 | 3 | 3 | 9 |
| 150554 | 58 | M | Yes | Yes | I | 1 | 0 | 0 | Alive | 41.5 | 1 | 2 | 2 | 3 | 3 | 9 |
| 150555 | 66 | M | Yes | No | II | 3 | 0 | 0 | Deceased | 23.7 | 1 | 1 | 1 | 2 | 3 | 6 |
| 150557 | 64 | F | No | No | II | 2 | 0 | 0 | Alive | 41.5 | 1 | 2 | 2 | 2 | 3 | 6 |
| 150560 | 70 | M | Yes | Yes | II | 3 | 0 | 0 | Deceased | 32.9 | 2 | 3 | 6 | 2 | 4 | 8 |
| 150561 | 58 | M | Yes | No | III | 3 | 1 | 0 | Deceased | 9.0 | 2 | 3 | 6 | 2 | 4 | 8 |
| 150573 | 68 | M | Yes | Yes | II | 2 | 0 | 0 | Alive | 41.4 | 2 | 2 | 4 | 3 | 2 | 6 |
| 150582 | 48 | F | No | No | I | 1 | 0 | 0 | Alive | 41.2 | 1 | 3 | 3 | 2 | 4 | 8 |
| 150601 | 64 | M | Yes | No | III | 4 | 0 | 0 | Alive | 41.1 | 2 | 2 | 4 | 2 | 4 | 8 |
| 150603 | 78 | M | Yes | Yes | III | 4 | 1 | 0 | Alive | 41.1 | 1 | 2 | 2 | 3 | 3 | 9 |
| 150606 | 58 | F | No | No | III | 3 | 1 | 0 | Deceased | 10.6 | 2 | 2 | 4 | 2 | 3 | 6 |
| 150609 | 49 | M | Yes | Yes | III | 3 | 1 | 0 | Deceased | 12.6 | 2 | 2 | 4 | 2 | 3 | 6 |
| 150613 | 53 | F | No | No | II | 1 | 1 | 0 | Alive | 41.0 | 2 | 3 | 6 | 2 | 3 | 6 |
| 150614 | 67 | M | Yes | Yes | III | 3 | 1 | 0 | Deceased | 1.9 | 2 | 3 | 6 | 3 | 3 | 9 |
| 150617 | 74 | F | No | No | II | 2 | 0 | 0 | Deceased | 28.8 | 2 | 3 | 6 | 2 | 4 | 8 |
| 150618 | 52 | M | Yes | Yes | II | 3 | 0 | 0 | Alive | 40.9 | 2 | 3 | 6 | 2 | 2 | 4 |
| 150620 | 64 | M | No | No | II | 2 | 1 | 0 | Deceased | 28.6 | 2 | 3 | 6 | 3 | 4 | 12 |
| 150622 | 75 | M | No | No | II | 2 | 0 | 0 | Deceased | 21.5 | 2 | 3 | 6 | 3 | 3 | 9 |
| 150623 | 62 | M | No | Yes | II | 3 | 0 | 0 | Deceased | 6.3 | 2 | 2 | 4 | 3 | 3 | 9 |
| 150627 | 58 | M | Yes | No | III | 3 | 3 | 0 | Deceased | 26.6 | 2 | 2 | 4 | 2 | 4 | 8 |
| 150631 | 61 | M | Yes | Yes | II | 3 | 0 | 0 | Alive | 40.8 | 2 | 1 | 2 | 2 | 3 | 6 |
| 150641 | 52 | M | Yes | Yes | III | 3 | 1 | 0 | Deceased | 6.0 | 2 | 3 | 6 | 3 | 3 | 9 |
| 150645 | 71 | F | No | No | II | 2 | 0 | 0 | Deceased | 12.6 | 1 | 3 | 3 | 2 | 3 | 6 |
| 150654 | 61 | M | Yes | Yes | III | 3 | 1 | 0 | Alive | 40.5 | 1 | 3 | 3 | 2 | 2 | 4 |
| 150660 | 71 | F | No | No | III | 2 | 2 | 0 | Deceased | 15.7 | 2 | 3 | 6 | 2 | 3 | 6 |
| 150662 | 57 | M | No | No | II | 1 | 1 | 0 | Alive | 40.4 | 2 | 2 | 4 | 2 | 3 | 6 |
| 150664 | 58 | M | Yes | Yes | III | 2 | 2 | 0 | Deceased | 12.2 | 2 | 3 | 6 | 2 | 3 | 6 |
| 150666 | 48 | M | Yes | Yes | I | 1 | 0 | 0 | Deceased | 6.2 | 1 | 3 | 3 | 3 | 2 | 6 |
| 150667 | 56 | M | Yes | Yes | II | 1 | 1 | 0 | Deceased | 22.8 | 2 | 3 | 6 | 3 | 3 | 9 |
| 150671 | 65 | F | No | No | II | 2 | 0 | 0 | Alive | 40.2 | 2 | 3 | 6 | 2 | 4 | 8 |
| 150703 | 44 | M | No | No | II | 2 | 1 | 0 | Alive | 40.1 | 2 | 3 | 6 | 2 | 3 | 6 |
| 150707 | 59 | M | Yes | Yes | II | 3 | 0 | 0 | Alive | 40.1 | 2 | 3 | 6 | 2 | 4 | 8 |
| 150710 | 67 | F | No | No | II | 2 | 0 | 0 | Alive | 40.0 | 2 | 3 | 6 | 2 | 3 | 6 |
| 150724 | 71 | M | Yes | No | II | 3 | 0 | 0 | Alive | 39.9 | 2 | 3 | 6 | 2 | 4 | 8 |
| 150726 | 69 | M | Yes | Yes | II | 2 | 0 | 0 | Deceased | 9.3 | 1 | 4 | 4 | 2 | 4 | 8 |
| 150735 | 59 | M | Yes | No | III | 2 | 2 | 0 | Deceased | 22.8 | 2 | 3 | 6 | 2 | 4 | 8 |
| 150736 | 60 | M | Yes | No | III | 3 | 1 | 0 | Alive | 39.7 | 2 | 3 | 6 | 3 | 3 | 9 |
| 150739 | 70 | M | Yes | Yes | II | 2 | 0 | 0 | Deceased | 7.9 | 1 | 2 | 2 | 3 | 3 | 9 |
| 150741 | 73 | M | Yes | No | III | 4 | 3 | 0 | Deceased | 21.4 | 2 | 3 | 6 | 2 | 4 | 8 |
| 150745 | 64 | F | No | No | III | 2 | 3 | 0 | Alive | 39.5 | 1 | 3 | 3 | 2 | 3 | 6 |
| 150747 | 64 | M | Yes | Yes | II | 3 | 0 | 0 | Alive | 39.5 | 1 | 3 | 3 | 1 | 4 | 4 |
| 150749 | 71 | M | Yes | No | III | 3 | 2 | 0 | Alive | 39.5 | 1 | 3 | 3 | 2 | 2 | 4 |
| 150762 | 59 | M | Yes | Yes | III | 3 | 1 | 0 | Deceased | 37.6 | 2 | 3 | 6 | 2 | 4 | 8 |
| 150770 | 73 | F | No | No | II | 3 | 0 | 0 | Deceased | 10.5 | 2 | 3 | 6 | 2 | 4 | 8 |
| 150805 | 51 | F | No | No | II | 3 | 0 | 0 | Alive | 39.0 | 2 | 1 | 2 | 2 | 4 | 8 |
| 150812 | 52 | M | Yes | Yes | III | 3 | 1 | 0 | Deceased | 5.4 | 2 | 3 | 6 | 2 | 4 | 8 |
| 150813 | 50 | M | No | No | I | 2 | 0 | 0 | Deceased | 1.6 | 2 | 3 | 6 | 2 | 4 | 8 |
| 150843 | 58 | M | Yes | Yes | III | 3 | 1 | 0 | Deceased | 23.0 | 2 | 3 | 6 | 2 | 4 | 8 |
| 150845 | 66 | F | No | No | I | 1 | 0 | 0 | Alive | 38.3 | 2 | 3 | 6 | 2 | 3 | 6 |
| 150846 | 74 | M | No | No | III | 3 | 1 | 0 | Alive | 38.3 | 2 | 3 | 6 | 2 | 4 | 8 |
| 150848 | 53 | F | No | No | III | 3 | 1 | 0 | Alive | 38.3 | 2 | 3 | 6 | 2 | 4 | 8 |
| 150854 | 60 | F | No | No | II | 3 | 0 | 0 | Deceased | 28.0 | 2 | 2 | 4 | 2 | 3 | 6 |
| 150855 | 67 | M | No | No | III | 3 | 1 | 0 | Deceased | 12.7 | 3 | 3 | 9 | 3 | 4 | 12 |
| 150901 | 57 | F | No | No | I | 1 | 0 | 0 | Alive | 38.0 | 2 | 2 | 4 | 2 | 4 | 8 |
| 150903 | 51 | M | Yes | Yes | III | 3 | 1 | 0 | Alive | 38.1 | 2 | 2 | 4 | 2 | 3 | 6 |
| 150906 | 55 | M | Yes | No | III | 3 | 2 | 0 | Alive | 37.9 | 2 | 2 | 4 | 3 | 4 | 12 |
| 150909 | 72 | M | Yes | No | II | 2 | 0 | 0 | Alive | 37.9 | 2 | 3 | 6 | 2 | 4 | 8 |
| 150914 | 63 | M | Yes | Yes | III | 3 | 1 | 0 | Deceased | 5.3 | 2 | 3 | 6 | 3 | 4 | 12 |
| 150918 | 63 | F | No | No | III | 2 | 3 | 0 | Alive | 37.6 | 2 | 3 | 6 | 2 | 3 | 6 |
| 150928 | 65 | M | Yes | No | II | 3 | 0 | 0 | Deceased | 18.3 | 2 | 3 | 6 | 3 | 4 | 12 |
| 150933 | 51 | M | Yes | Yes | II | 3 | 0 | 0 | Alive | 37.4 | 2 | 3 | 6 | 3 | 3 | 9 |
| 150934 | 61 | M | Yes | Yes | I | 3 | 0 | 0 | Alive | 37.4 | 2 | 3 | 6 | 2 | 3 | 6 |
| 150936 | 65 | M | Yes | Yes | II | 3 | 0 | 0 | Alive | 37.4 | 2 | 3 | 6 | 2 | 4 | 8 |
| 150947 | 61 | M | No | Yes | II | 3 | 0 | 0 | Alive | 37.5 | 2 | 3 | 6 | 2 | 3 | 6 |
| 150953 | 68 | M | Yes | Yes | III | 3 | 1 | 0 | Deceased | 5.8 | 2 | 3 | 6 | 2 | 3 | 6 |
| 150954 | 65 | F | No | No | II | 3 | 0 | 0 | Alive | 37.5 | 2 | 3 | 6 | 3 | 3 | 9 |
| 151002 | 58 | F | No | No | II | 3 | 0 | 0 | Alive | 37.2 | 2 | 3 | 6 | 3 | 4 | 12 |
| 151011 | 67 | M | Yes | No | III | 3 | 1 | 0 | Deceased | 4.3 | 1 | 3 | 3 | 3 | 4 | 12 |
| 151012 | 69 | F | No | No | II | 3 | 0 | 0 | Deceased | 32.7 | 1 | 3 | 3 | 2 | 3 | 6 |
| 151016 | 63 | M | Yes | Yes | II | 2 | 0 | 0 | Deceased | 34.3 | 1 | 3 | 3 | 1 | 4 | 4 |
| 151017 | 56 | M | Yes | Yes | III | 3 | 1 | 0 | Alive | 37.1 | 1 | 2 | 2 | 2 | 2 | 4 |
| 151020 | 68 | F | No | No | II | 2 | 0 | 0 | Alive | 37.0 | 2 | 3 | 6 | 2 | 3 | 6 |
| 151021 | 62 | M | Yes | Yes | II | 3 | 0 | 0 | Deceased | 15.8 | 2 | 3 | 6 | 3 | 3 | 9 |
| 151024 | 55 | F | No | No | II | 3 | 0 | 0 | Alive | 36.9 | 2 | 3 | 6 | 2 | 4 | 8 |
| 151033 | 50 | M | Yes | Yes | II | 3 | 0 | 0 | Alive | 36.7 | 2 | 3 | 6 | 3 | 3 | 9 |
| 151040 | 67 | M | No | No | II | 2 | 1 | 0 | Alive | 36.7 | 2 | 3 | 6 | 2 | 4 | 8 |
| 151044 | 52 | M | Yes | Yes | III | 3 | 1 | 0 | Deceased | 25.2 | 2 | 3 | 6 | 3 | 3 | 9 |
| 151047 | 65 | M | Yes | Yes | II | 3 | 0 | 0 | Alive | 36.5 | 2 | 3 | 6 | 2 | 3 | 6 |
| 151049 | 52 | M | Yes | Yes | III | 3 | 1 | 0 | Alive | 36.5 | 2 | 2 | 4 | 2 | 3 | 6 |
| 151050 | 46 | M | Yes | Yes | II | 3 | 0 | 0 | Alive | 36.5 | 2 | 2 | 4 | 2 | 3 | 6 |
| 151052 | 61 | M | Yes | Yes | II | 2 | 1 | 0 | Deceased | 19.9 | 2 | 2 | 4 | 2 | 4 | 8 |
| 151053 | 56 | F | No | No | I | 1 | 0 | 0 | Alive | 36.5 | 2 | 3 | 6 | 2 | 3 | 6 |
| 151107 | 68 | M | No | No | III | 3 | 3 | 0 | Alive | 36.5 | 1 | 3 | 3 | 2 | 3 | 6 |
| 160105 | 66 | F | No | No | II | 2 | 0 | 0 | Alive | 33.7 | 2 | 2 | 4 | 2 | 4 | 8 |
| 160106 | 56 | F | No | No | III | 3 | 1 | 0 | Alive | 33.7 | 2 | 2 | 4 | 2 | 3 | 6 |
| 160110 | 62 | F | No | No | I | 3 | 0 | 0 | Alive | 33.8 | 1 | 2 | 2 | 2 | 3 | 6 |
| 160111 | 66 | F | No | Yes | II | 3 | 0 | 0 | Deceased | 20.6 | 2 | 1 | 2 | 2 | 4 | 8 |
| 160114 | 72 | F | No | No | II | 3 | 0 | 0 | Alive | 33.8 | 1 | 2 | 2 | 2 | 3 | 6 |
| 160117 | 75 | M | Yes | Yes | III | 3 | 1 | 0 | Deceased | 14.4 | 1 | 1 | 1 | 2 | 3 | 6 |
| 160119 | 67 | M | No | No | I | 1 | 0 | 0 | Alive | 33.8 | 3 | 2 | 6 | 2 | 3 | 6 |
| 160139 | 65 | M | Yes | Yes | I | 1 | 0 | 0 | Alive | 33.5 | 2 | 2 | 4 | 2 | 3 | 6 |
| 160140 | 56 | M | Yes | Yes | III | 3 | 2 | 0 | Deceased | 23.4 | 2 | 2 | 4 | 3 | 3 | 9 |
| 160143 | 62 | M | Yes | Yes | II | 2 | 0 | 0 | Alive | 33.4 | 2 | 2 | 4 | 3 | 2 | 6 |
| 160161 | 59 | M | Yes | No | IV | 3 | 2 | 1 | Deceased | 17.8 | 2 | 3 | 6 | 3 | 3 | 9 |
| 160204 | 64 | M | Yes | No | II | 2 | 1 | 0 | Alive | 32.6 | 2 | 3 | 6 | 2 | 3 | 6 |
| 160209 | 57 | M | Yes | No | II | 2 | 1 | 0 | Alive | 32.6 | 2 | 2 | 4 | 1 | 3 | 3 |
| 160211 | 72 | M | Yes | Yes | II | 3 | 0 | 0 | Alive | 32.6 | 2 | 3 | 6 | 3 | 3 | 9 |
| 160218 | 64 | M | Yes | No | I | 1 | 0 | 0 | Alive | 32.5 | 2 | 3 | 6 | 2 | 2 | 4 |
| 160219 | 66 | M | Yes | No | III | 3 | 3 | 0 | Deceased | 20.1 | 2 | 2 | 4 | 2 | 3 | 6 |
| 160222 | 52 | M | Yes | Yes | II | 3 | 0 | 0 | Alive | 32.5 | 2 | 3 | 6 | 3 | 3 | 9 |
| 160224 | 54 | M | No | Yes | III | 3 | 1 | 0 | Alive | 32.5 | 1 | 3 | 3 | 2 | 2 | 4 |
| 160227 | 72 | F | No | No | I | 1 | 0 | 0 | Alive | 32.5 | 2 | 3 | 6 | 3 | 2 | 6 |
| 160228 | 71 | M | Yes | No | III | 3 | 1 | 0 | Alive | 32.5 | 2 | 2 | 4 | 3 | 3 | 9 |
| 160229 | 75 | F | No | No | II | 2 | 0 | 0 | Alive | 32.5 | 1 | 2 | 2 | 3 | 3 | 9 |
| 160236 | 62 | M | Yes | Yes | III | 3 | 3 | 0 | Deceased | 18.9 | 2 | 3 | 6 | 3 | 3 | 9 |
| 160238 | 63 | M | No | Yes | III | 3 | 2 | 0 | Deceased | 12.8 | 3 | 3 | 6 | 3 | 3 | 9 |
| 160246 | 70 | F | No | No | II | 3 | 0 | 0 | Alive | 32.3 | 1 | 2 | 2 | 1 | 4 | 4 |
| 160248 | 49 | M | Yes | Yes | II | 3 | 0 | 0 | Alive | 32.3 | 1 | 2 | 2 | 2 | 2 | 4 |
| 160301 | 67 | F | No | No | III | 3 | 1 | 0 | Deceased | 7.6 | 3 | 2 | 6 | 3 | 3 | 9 |
| 160303 | 55 | M | Yes | Yes | III | 3 | 1 | 0 | Alive | 32.3 | 2 | 3 | 6 | 3 | 3 | 9 |
| 160309 | 63 | M | Yes | No | II | 3 | 0 | 0 | Alive | 32.2 | 2 | 2 | 4 | 3 | 3 | 9 |
| 160310 | 69 | M | Yes | Yes | II | 3 | 0 | 0 | Deceased | 9.5 | 2 | 3 | 6 | 3 | 3 | 9 |
| 160311 | 52 | M | Yes | Yes | III | 3 | 1 | 0 | Deceased | 18.8 | 2 | 3 | 6 | 2 | 4 | 8 |
| 160319 | 57 | M | Yes | Yes | III | 3 | 2 | 0 | Deceased | 21.6 | 2 | 3 | 6 | 3 | 3 | 9 |
| 160320 | 64 | M | Yes | Yes | II | 3 | 0 | 0 | Alive | 32.2 | 2 | 3 | 6 | 2 | 4 | 8 |
| 160324 | 53 | M | Yes | Yes | II | 3 | 0 | 0 | Deceased | 16.4 | 2 | 3 | 6 | 3 | 4 | 12 |
| 160326 | 65 | M | Yes | Yes | II | 3 | 0 | 0 | Alive | 32.0 | 2 | 3 | 6 | 3 | 3 | 9 |
| 160333 | 61 | M | Yes | Yes | III | 2 | 3 | 0 | Deceased | 13.3 | 2 | 3 | 6 | 3 | 4 | 12 |
| 160334 | 64 | M | Yes | No | II | 3 | 0 | 0 | Alive | 31.9 | 2 | 3 | 6 | 3 | 4 | 12 |
| 160342 | 71 | M | Yes | No | II | 3 | 0 | 0 | Deceased | 15.7 | 2 | 4 | 8 | 3 | 4 | 12 |
| 160343 | 66 | M | Yes | Yes | II | 3 | 0 | 0 | Alive | 31.8 | 2 | 3 | 6 | 3 | 2 | 6 |
| 160351 | 66 | F | No | No | II | 3 | 0 | 0 | Deceased | 12.2 | 2 | 3 | 6 | 2 | 4 | 8 |
| 160362 | 76 | M | No | No | III | 3 | 1 | 0 | Deceased | 11.8 | 2 | 3 | 6 | 3 | 3 | 9 |
| 160365 | 59 | F | No | No | II | 2 | 1 | 0 | Deceased | 2.6 | 2 | 4 | 8 | 3 | 4 | 12 |
| 160372 | 53 | F | No | No | I | 1 | 0 | 0 | Alive | 31.4 | 2 | 2 | 4 | 2 | 3 | 6 |
| 160375 | 60 | F | No | No | III | 3 | 1 | 0 | Alive | 31.3 | 2 | 3 | 6 | 2 | 3 | 6 |
| 160411 | 61 | M | Yes | Yes | II | 3 | 0 | 0 | Deceased | 19.6 | 1 | 3 | 3 | 2 | 4 | 8 |
| 160413 | 57 | M | No | Yes | III | 3 | 1 | 0 | Deceased | 19.6 | 2 | 1 | 2 | 2 | 4 | 8 |
| 160414 | 62 | M | No | No | I | 1 | 0 | 0 | Alive | 31.2 | 1 | 1 | 1 | 2 | 3 | 6 |
| 160432 | 73 | M | Yes | No | III | 3 | 3 | 0 | Deceased | 5.6 | 2 | 3 | 6 | 2 | 3 | 6 |
| 160439 | 66 | M | Yes | Yes | II | 3 | 0 | 0 | Deceased | 10.1 | 2 | 3 | 6 | 2 | 4 | 8 |
| 160441 | 75 | M | No | No | II | 3 | 0 | 0 | Deceased | 19.6 | 1 | 1 | 1 | 1 | 3 | 3 |
| 160443 | 61 | F | No | No | III | 3 | 3 | 0 | Deceased | 19.3 | 2 | 3 | 6 | 2 | 4 | 8 |
| 160458 | 66 | M | Yes | Yes | II | 3 | 0 | 0 | Deceased | 22.4 | 2 | 3 | 6 | 2 | 4 | 8 |
| 160460 | 59 | M | Yes | Yes | II | 3 | 0 | 0 | Alive | 30.6 | 2 | 3 | 6 | 2 | 3 | 6 |
| 160461 | 63 | M | Yes | Yes | II | 2 | 1 | 0 | Deceased | 9.8 | 2 | 3 | 6 | 2 | 4 | 8 |
| 160466 | 66 | M | Yes | Yes | III | 3 | 1 | 0 | Alive | 30.5 | 1 | 1 | 1 | 2 | 3 | 6 |
| 160467 | 73 | M | No | Yes | I | 1 | 0 | 0 | Alive | 30.3 | 2 | 3 | 6 | 2 | 4 | 8 |
| 160473 | 48 | M | Yes | Yes | II | 3 | 0 | 0 | Alive | 30.6 | 1 | 1 | 1 | 1 | 3 | 3 |
| 160474 | 51 | M | Yes | Yes | III | 3 | 1 | 0 | Alive | 30.6 | 1 | 1 | 1 | 2 | 3 | 6 |
| 1503100 | 51 | M | Yes | Yes | II | 3 | 0 | 0 | Alive | 43.8 | 2 | 2 | 4 | 3 | 3 | 9 |
| 1503101 | 63 | F | No | No | II | 2 | 1 | 0 | Alive | 43.8 | 2 | 2 | 4 | 2 | 3 | 6 |

^a^M, male; F, female.

^b^Individuals who smoked an average of <1 cigarette/d and for <1 year in their lifetime were defined as nonsmokers; otherwise, they were defined as smokers. Individuals were classified as drinkers if they drank at least twice a week and continuously for at least 1 year during their lifetime; otherwise, they were defined as nondrinkers.

^c^Tumor TNM staging components, including tumor (T), lymph node (N) and metastasis (M), were reviewed by at least 3 pathologists and defined according to the American Joint Committee on Cancer (AJCC) 7th edition.

^d^IRS, immune reactive score. IRS was obtained by multiplying the score of positive and that of intensity. The labeling score of positive was defined as 1 (≤10%), 2 (11%⎯50%), 3 (51%⎯80%) and 4 (>80%); the labeling score of intensity was estimated as negative (0), weak (1), moderate (2) and strong (3).

**Supplementary Table 3. Annotation information of identified SNPs**

| SNP | Position (hg38) | Gene | location |
| --- | --- | --- | --- |
| rs7720479 | 6639936 | SRD5A1 | intron |
| rs4702374 | 6638316 | SRD5A1 | intron |
| rs4702375 | 6638479 | SRD5A1 | intron |
| rs8192139 | 6636860 | SRD5A1 | intron |
| rs10044539 | 6637936 | SRD5A1 | intron |
| rs10076470 | 6632762 | NSUN2 | intron |
| rs10062086 | 6632929 | NSUN2 | exon |

**Supplementary Table 4. Overlap between 509 genes identified by RNA-sequencing in KYSE150 cells with NSUN2 knockout and upregulated genes identified in 170 ESCC**

| Gene symbol | *NSUN2* KO #1 | | *NSUN2* KO #2 | | 170 ESCC | |
| --- | --- | --- | --- | --- | --- | --- |
|  | Fold change | *P* value | Fold change | *P* value | Fold change | *FDR* value |
| ABCA12 | 0.45 | 4.17E-02 | 0.35 | 1.93E-02 | 4.60 | 2.27E-19 |
| ACD | 0.66 | 6.47E-04 | 0.65 | 2.65E-03 | 1.72 | 1.35E-35 |
| ACOT7 | 0.78 | 4.17E-05 | 0.72 | 4.94E-03 | 2.21 | 8.17E-49 |
| ACTR5 | 0.75 | 5.47E-03 | 0.79 | 4.04E-03 | 1.48 | 1.70E-30 |
| ACYP1 | 0.78 | 3.43E-02 | 0.59 | 3.19E-03 | 1.71 | 8.95E-35 |
| ADM2 | 0.04 | 5.85E-05 | 0.22 | 1.28E-04 | 1.56 | 1.11E-05 |
| ADRM1 | 0.64 | 7.78E-03 | 0.57 | 1.50E-03 | 1.77 | 1.23E-47 |
| AGAP2-AS1 | 0.75 | 1.15E-02 | 0.52 | 4.05E-03 | 2.54 | 6.54E-44 |
| AIMP2 | 0.50 | 8.90E-04 | 0.69 | 6.83E-03 | 1.73 | 8.61E-38 |
| ALDH16A1 | 0.59 | 7.04E-03 | 0.68 | 3.54E-03 | 1.59 | 2.49E-30 |
| ALG1 | 0.49 | 1.38E-02 | 0.69 | 3.76E-02 | 1.76 | 1.19E-47 |
| ALPP | 0.16 | 4.82E-05 | 0.07 | 4.31E-04 | 2.14 | 2.87E-12 |
| ALYREF | 0.72 | 1.73E-02 | 0.59 | 1.71E-02 | 1.92 | 2.63E-43 |
| AMH | 0.28 | 1.34E-02 | 0.55 | 4.08E-02 | 1.87 | 2.54E-11 |
| ANKRD39 | 0.67 | 3.96E-03 | 0.58 | 1.31E-03 | 1.61 | 3.07E-31 |
| ANKRD52 | 0.77 | 7.95E-03 | 0.54 | 1.44E-02 | 1.53 | 1.20E-36 |
| AP5S1 | 0.66 | 6.48E-03 | 0.63 | 4.11E-03 | 1.53 | 3.03E-31 |
| APEX1 | 0.59 | 2.46E-03 | 0.56 | 4.95E-04 | 1.60 | 1.55E-38 |
| APEX2 | 0.72 | 1.58E-02 | 0.59 | 1.49E-03 | 1.62 | 5.33E-27 |
| APLN | 0.48 | 8.51E-04 | 0.20 | 6.61E-04 | 2.75 | 1.02E-27 |
| ARHGAP39 | 0.58 | 8.19E-03 | 0.47 | 6.61E-03 | 1.53 | 8.19E-14 |
| ARMC6 | 0.58 | 3.16E-04 | 0.71 | 1.28E-03 | 1.50 | 8.32E-32 |
| ARRB2 | 0.65 | 1.65E-05 | 0.64 | 1.92E-02 | 1.65 | 1.21E-22 |
| ASF1B | 0.43 | 2.65E-04 | 0.59 | 5.38E-04 | 4.07 | 8.84E-40 |
| ATAD3A | 0.59 | 1.23E-03 | 0.47 | 4.16E-03 | 2.05 | 7.54E-51 |
| ATAD3B | 0.65 | 3.85E-02 | 0.44 | 6.69E-03 | 1.70 | 1.82E-36 |
| ATG101 | 0.68 | 4.71E-03 | 0.47 | 4.48E-04 | 1.59 | 1.59E-42 |
| ATP6V0B | 0.74 | 2.40E-03 | 0.62 | 5.25E-03 | 1.82 | 1.27E-45 |
| AUNIP | 0.77 | 1.07E-02 | 0.75 | 2.51E-02 | 3.36 | 3.03E-50 |
| AURKB | 0.71 | 2.17E-02 | 0.45 | 9.63E-04 | 6.15 | 1.65E-43 |
| B3GNT9 | 0.25 | 4.39E-04 | 0.77 | 7.85E-03 | 1.76 | 4.76E-30 |
| BAK1 | 0.76 | 1.99E-02 | 0.59 | 4.07E-03 | 2.04 | 2.29E-39 |
| BANF1 | 0.72 | 2.60E-03 | 0.55 | 1.02E-03 | 1.95 | 2.31E-50 |
| BAX | 0.55 | 1.75E-03 | 0.55 | 1.98E-03 | 1.96 | 4.67E-58 |
| BCL2L12 | 0.65 | 6.79E-04 | 0.66 | 3.12E-03 | 2.63 | 4.37E-57 |
| BEX3 | 0.67 | 5.82E-03 | 0.30 | 2.38E-03 | 1.65 | 3.19E-25 |
| BOLA2 | 0.60 | 2.36E-02 | 0.54 | 1.39E-02 | 2.15 | 2.87E-50 |
| BOP1 | 0.73 | 5.02E-03 | 0.48 | 4.99E-04 | 2.91 | 1.45E-52 |
| BRAT1 | 0.49 | 1.38E-04 | 0.79 | 2.01E-02 | 1.98 | 5.29E-48 |
| BRD9 | 0.65 | 1.77E-03 | 0.37 | 7.97E-04 | 1.65 | 3.79E-38 |
| BRF1 | 0.74 | 1.87E-02 | 0.62 | 1.11E-02 | 1.42 | 1.02E-23 |
| BRI3BP | 0.58 | 3.48E-03 | 0.43 | 4.45E-03 | 1.83 | 1.48E-23 |
| BRMS1 | 0.58 | 3.39E-04 | 0.44 | 1.64E-04 | 2.28 | 1.24E-49 |
| BYSL | 0.68 | 1.13E-02 | 0.64 | 1.08E-02 | 2.03 | 6.10E-44 |
| C17orf107 | 0.53 | 2.56E-03 | 0.33 | 1.63E-03 | 1.41 | 1.06E-09 |
| C19orf48 | 0.60 | 5.32E-03 | 0.72 | 1.92E-02 | 2.30 | 8.01E-42 |
| C19orf53 | 0.68 | 1.73E-03 | 0.40 | 1.46E-02 | 1.49 | 3.38E-34 |
| C1orf216 | 0.32 | 6.37E-04 | 0.78 | 3.61E-02 | 1.53 | 8.80E-15 |
| C1orf35 | 0.58 | 9.83E-03 | 0.44 | 4.00E-03 | 1.51 | 8.05E-31 |
| C1QBP | 0.61 | 1.96E-04 | 0.39 | 8.83E-04 | 1.57 | 6.94E-27 |
| C21orf58 | 0.55 | 1.21E-02 | 0.70 | 3.65E-02 | 1.74 | 1.11E-23 |
| C2CD3 | 0.59 | 3.72E-04 | 0.62 | 2.87E-02 | 1.53 | 2.62E-21 |
| C2orf81 | 0.58 | 2.80E-02 | 0.59 | 1.80E-02 | 1.42 | 8.71E-11 |
| C4orf48 | 0.78 | 3.00E-02 | 0.58 | 4.61E-03 | 4.90 | 9.98E-61 |
| C5orf38 | 0.54 | 1.33E-02 | 0.13 | 8.53E-05 | 2.73 | 4.61E-20 |
| CA9 | 0.26 | 1.84E-03 | 0.21 | 4.15E-03 | 14.07 | 8.08E-37 |
| CAD | 0.77 | 4.61E-03 | 0.71 | 4.33E-02 | 2.44 | 2.34E-62 |
| CALML5 | 0.00 | 4.79E-02 | 0.00 | 4.79E-02 | 2.18 | 2.12E-04 |
| CALR | 0.75 | 4.50E-02 | 0.79 | 4.49E-02 | 1.82 | 3.56E-45 |
| CAMK2N2 | 0.62 | 3.14E-03 | 0.58 | 4.74E-02 | 3.08 | 4.20E-44 |
| CAMKK1 | 0.77 | 1.30E-02 | 0.51 | 3.69E-04 | 1.43 | 5.77E-16 |
| CAPN15 | 0.70 | 1.20E-02 | 0.65 | 8.24E-03 | 1.45 | 4.31E-20 |
| CARD10 | 0.74 | 1.96E-03 | 0.65 | 5.49E-03 | 1.77 | 5.45E-18 |
| CARD19 | 0.73 | 3.57E-02 | 0.65 | 1.99E-02 | 1.54 | 2.17E-25 |
| CBX2 | 0.51 | 2.18E-04 | 0.77 | 3.86E-03 | 3.02 | 1.66E-32 |
| CCDC137 | 0.65 | 6.83E-03 | 0.47 | 6.50E-04 | 1.55 | 3.05E-22 |
| CCDC189 | 0.39 | 4.14E-02 | 0.36 | 2.31E-02 | 1.80 | 2.46E-34 |
| CCDC34 | 0.57 | 4.15E-03 | 0.65 | 7.53E-03 | 2.19 | 8.63E-40 |
| CCDC86 | 0.52 | 8.65E-03 | 0.51 | 4.23E-03 | 2.26 | 1.17E-49 |
| CCHCR1 | 0.70 | 1.53E-02 | 0.64 | 7.12E-04 | 1.60 | 1.39E-30 |
| CCNF | 0.65 | 1.74E-03 | 0.63 | 6.65E-03 | 3.66 | 9.85E-52 |
| CCNO | 0.54 | 5.83E-04 | 0.47 | 9.27E-03 | 2.30 | 7.47E-19 |
| CCT2 | 0.77 | 2.54E-03 | 0.57 | 1.41E-03 | 1.81 | 2.10E-37 |
| CD320 | 0.71 | 3.33E-03 | 0.55 | 1.30E-02 | 1.58 | 6.92E-20 |
| CD40 | 0.20 | 7.72E-03 | 0.08 | 8.84E-03 | 1.57 | 8.77E-13 |
| CDC20 | 0.68 | 9.97E-03 | 0.39 | 2.45E-03 | 6.88 | 4.92E-47 |
| CDC25A | 0.67 | 1.08E-02 | 0.53 | 1.01E-02 | 2.71 | 4.46E-34 |
| CDC45 | 0.62 | 4.77E-03 | 0.75 | 2.48E-02 | 5.91 | 6.65E-44 |
| CDCA4 | 0.71 | 2.01E-04 | 0.57 | 1.07E-03 | 2.60 | 2.16E-41 |
| CDCA5 | 0.68 | 4.33E-03 | 0.78 | 1.67E-02 | 5.83 | 1.27E-56 |
| CDK2AP1 | 0.62 | 4.13E-03 | 0.61 | 2.05E-03 | 1.63 | 1.20E-35 |
| CDK4 | 0.77 | 3.18E-03 | 0.66 | 2.78E-03 | 2.40 | 4.77E-59 |
| CDK5 | 0.77 | 3.17E-02 | 0.67 | 1.66E-02 | 1.80 | 5.53E-41 |
| CDK5RAP1 | 0.78 | 3.00E-02 | 0.72 | 1.29E-02 | 1.70 | 6.96E-49 |
| CDT1 | 0.57 | 7.13E-04 | 0.74 | 1.65E-02 | 4.50 | 6.35E-45 |
| CEBPA | 0.75 | 3.22E-02 | 0.50 | 2.96E-03 | 1.42 | 6.85E-08 |
| CENPA | 0.77 | 2.71E-02 | 0.70 | 2.40E-02 | 5.66 | 1.76E-48 |
| CEP131 | 0.58 | 1.82E-02 | 0.39 | 4.33E-03 | 1.97 | 1.60E-48 |
| CEP72 | 0.53 | 2.14E-03 | 0.37 | 4.15E-04 | 2.80 | 8.01E-51 |
| CHAC2 | 0.52 | 7.31E-03 | 0.74 | 4.76E-02 | 1.47 | 8.54E-11 |
| CHAF1A | 0.53 | 1.58E-04 | 0.65 | 4.41E-02 | 2.33 | 1.35E-52 |
| CHAF1B | 0.56 | 4.81E-04 | 0.63 | 3.59E-03 | 2.04 | 2.26E-40 |
| CHST14 | 0.57 | 2.88E-03 | 0.66 | 2.91E-02 | 1.74 | 1.00E-26 |
| CLPTM1L | 0.68 | 1.52E-02 | 0.49 | 6.14E-03 | 2.11 | 1.88E-50 |
| CLSPN | 0.57 | 1.37E-03 | 0.67 | 3.51E-02 | 4.72 | 2.01E-42 |
| COA7 | 0.63 | 2.77E-02 | 0.52 | 1.30E-02 | 1.71 | 8.33E-33 |
| COASY | 0.73 | 9.09E-03 | 0.68 | 1.46E-02 | 1.51 | 1.21E-25 |
| COL4A2 | 0.55 | 2.55E-03 | 0.55 | 3.51E-03 | 2.07 | 4.71E-14 |
| COL9A2 | 0.31 | 1.65E-02 | 0.36 | 2.14E-02 | 2.10 | 1.94E-14 |
| COMMD4 | 0.55 | 1.17E-03 | 0.57 | 1.70E-03 | 1.64 | 3.43E-47 |
| COPRS | 0.66 | 5.82E-03 | 0.73 | 1.20E-02 | 1.45 | 2.20E-18 |
| CPSF4 | 0.74 | 2.86E-02 | 0.79 | 4.83E-02 | 1.65 | 1.96E-32 |
| CTDNEP1 | 0.74 | 1.32E-03 | 0.68 | 6.71E-03 | 1.48 | 3.06E-37 |
| CTNS | 0.42 | 2.55E-02 | 0.56 | 3.39E-02 | 1.65 | 2.19E-42 |
| CTPS1 | 0.72 | 1.81E-02 | 0.47 | 1.98E-03 | 1.97 | 1.10E-38 |
| CTSA | 0.76 | 3.35E-03 | 0.73 | 1.68E-02 | 1.81 | 5.18E-45 |
| CYP24A1 | 0.54 | 4.25E-03 | 0.55 | 1.37E-02 | 5.72 | 3.80E-18 |
| CYP27C1 | 0.64 | 2.34E-02 | 0.44 | 2.79E-03 | 3.32 | 1.67E-27 |
| DAZAP1 | 0.74 | 1.18E-02 | 0.61 | 3.16E-03 | 1.41 | 1.08E-32 |
| DCTPP1 | 0.59 | 1.46E-04 | 0.70 | 3.53E-02 | 2.26 | 8.81E-57 |
| DDX28 | 0.65 | 1.53E-02 | 0.73 | 3.04E-03 | 1.48 | 5.92E-30 |
| DDX39A | 0.60 | 2.52E-02 | 0.56 | 1.94E-02 | 2.49 | 9.30E-60 |
| DDX49 | 0.74 | 7.54E-03 | 0.78 | 1.02E-02 | 1.60 | 4.70E-38 |
| DDX54 | 0.75 | 3.43E-02 | 0.30 | 1.09E-03 | 1.51 | 2.40E-35 |
| DGKZ | 0.76 | 1.10E-02 | 0.78 | 1.71E-02 | 1.45 | 1.27E-24 |
| DHRS13 | 0.61 | 2.00E-03 | 0.56 | 2.85E-03 | 1.87 | 4.51E-29 |
| DHX16 | 0.74 | 1.10E-02 | 0.67 | 2.16E-03 | 1.42 | 6.15E-35 |
| DHX33 | 0.61 | 7.57E-03 | 0.73 | 1.03E-02 | 1.54 | 1.14E-32 |
| DHX37 | 0.63 | 1.00E-03 | 0.44 | 3.51E-04 | 1.64 | 3.75E-40 |
| DHX9 | 0.65 | 3.36E-03 | 0.73 | 7.03E-03 | 1.58 | 2.70E-52 |
| DLK2 | 0.55 | 4.80E-03 | 0.30 | 6.35E-04 | 1.56 | 1.14E-04 |
| DNAAF3 | 0.61 | 1.16E-02 | 0.71 | 2.49E-02 | 1.70 | 2.94E-08 |
| DNAAF5 | 0.65 | 2.41E-03 | 0.72 | 4.03E-03 | 1.59 | 1.14E-30 |
| DNAJA3 | 0.77 | 2.83E-03 | 0.80 | 3.38E-02 | 1.45 | 2.07E-25 |
| DNMT1 | 0.74 | 1.29E-02 | 0.62 | 2.58E-03 | 2.30 | 3.66E-58 |
| DOHH | 0.59 | 1.77E-03 | 0.42 | 5.82E-04 | 1.45 | 9.44E-29 |
| DOLK | 0.60 | 1.19E-02 | 0.66 | 1.15E-02 | 1.51 | 2.08E-32 |
| DOLPP1 | 0.74 | 7.60E-03 | 0.72 | 6.81E-03 | 1.45 | 2.03E-12 |
| DPH2 | 0.68 | 1.07E-02 | 0.47 | 1.04E-03 | 1.77 | 1.56E-34 |
| DTYMK | 0.61 | 2.66E-04 | 0.65 | 1.01E-02 | 2.33 | 1.88E-56 |
| DUSP9 | 0.00 | 1.91E-02 | 0.00 | 1.91E-02 | 9.84 | 1.24E-43 |
| DVL2 | 0.59 | 5.03E-03 | 0.68 | 1.12E-02 | 1.41 | 4.03E-19 |
| E2F1 | 0.57 | 1.88E-03 | 0.78 | 2.24E-02 | 3.68 | 1.81E-67 |
| E2F2 | 0.37 | 2.08E-04 | 0.78 | 1.85E-02 | 1.47 | 7.21E-05 |
| EARS2 | 0.75 | 5.81E-03 | 0.78 | 2.60E-02 | 1.42 | 2.57E-25 |
| EBNA1BP2 | 0.73 | 1.28E-02 | 0.52 | 2.72E-03 | 1.86 | 7.12E-37 |
| EEF2KMT | 0.64 | 2.83E-02 | 0.73 | 1.53E-03 | 1.59 | 1.02E-34 |
| EEFSEC | 0.71 | 7.20E-03 | 0.67 | 2.70E-02 | 1.56 | 1.08E-36 |
| EFNA4 | 0.74 | 4.66E-02 | 0.63 | 1.78E-02 | 1.90 | 9.33E-23 |
| EHMT2 | 0.63 | 1.58E-02 | 0.72 | 2.47E-02 | 1.49 | 6.41E-29 |
| EIF4A3 | 0.71 | 1.94E-03 | 0.61 | 2.12E-03 | 1.46 | 1.11E-21 |
| EIF4EBP1 | 0.70 | 2.50E-02 | 0.49 | 1.53E-03 | 1.92 | 2.24E-24 |
| EIF4G1 | 0.77 | 5.54E-03 | 0.76 | 7.14E-03 | 1.53 | 2.50E-28 |
| EIF5A | 0.66 | 6.86E-05 | 0.54 | 1.85E-04 | 1.52 | 1.73E-21 |
| ELOF1 | 0.55 | 4.23E-04 | 0.44 | 2.28E-03 | 1.54 | 5.85E-43 |
| EMC6 | 0.52 | 5.73E-03 | 0.45 | 1.59E-03 | 1.41 | 5.96E-28 |
| EMC8 | 0.71 | 3.33E-03 | 0.76 | 5.55E-03 | 1.68 | 2.10E-47 |
| EMC9 | 0.75 | 8.58E-03 | 0.75 | 1.05E-02 | 1.54 | 2.11E-22 |
| EPPK1 | 0.60 | 5.86E-03 | 0.52 | 3.56E-03 | 2.32 | 1.30E-18 |
| ERAL1 | 0.71 | 5.41E-03 | 0.67 | 4.06E-03 | 1.56 | 1.08E-35 |
| ERF | 0.67 | 1.64E-02 | 0.63 | 7.71E-04 | 1.68 | 4.90E-23 |
| ETV4 | 0.79 | 1.92E-02 | 0.74 | 1.64E-02 | 3.51 | 3.96E-30 |
| EXO5 | 0.41 | 3.38E-03 | 0.45 | 4.53E-03 | 1.41 | 5.94E-23 |
| EXOSC3 | 0.78 | 3.63E-02 | 0.79 | 2.32E-02 | 1.41 | 4.72E-18 |
| EXOSC4 | 0.59 | 1.56E-02 | 0.60 | 2.02E-02 | 2.23 | 1.34E-50 |
| F12 | 0.67 | 3.94E-02 | 0.60 | 1.77E-03 | 1.82 | 1.73E-08 |
| FADD | 0.67 | 4.43E-03 | 0.55 | 1.44E-03 | 4.24 | 3.96E-50 |
| FAM136A | 0.61 | 1.01E-02 | 0.78 | 2.91E-02 | 1.49 | 5.33E-34 |
| FAM207A | 0.46 | 6.33E-03 | 0.28 | 1.33E-03 | 1.61 | 3.65E-34 |
| FAM222A | 0.40 | 9.29E-03 | 0.23 | 3.63E-03 | 1.67 | 1.05E-19 |
| FANCE | 0.76 | 2.48E-02 | 0.67 | 4.92E-03 | 2.30 | 2.00E-40 |
| FARSA | 0.64 | 1.83E-03 | 0.47 | 4.39E-04 | 1.73 | 3.93E-38 |
| FBRSL1 | 0.59 | 2.90E-03 | 0.34 | 4.06E-03 | 1.48 | 2.03E-14 |
| FBXL19 | 0.60 | 1.11E-02 | 0.68 | 1.50E-02 | 1.70 | 4.31E-34 |
| FBXL6 | 0.78 | 8.44E-03 | 0.73 | 1.10E-02 | 2.21 | 2.20E-50 |
| FDX2 | 0.56 | 2.12E-04 | 0.54 | 4.91E-03 | 1.64 | 5.11E-36 |
| FEN1 | 0.45 | 1.08E-03 | 0.53 | 8.08E-04 | 3.04 | 2.63E-59 |
| FKBP4 | 0.77 | 3.38E-03 | 0.58 | 9.43E-04 | 1.59 | 5.40E-18 |
| FLAD1 | 0.66 | 1.94E-03 | 0.68 | 1.57E-02 | 2.03 | 7.65E-53 |
| FOXP4 | 0.63 | 4.00E-03 | 0.69 | 5.09E-03 | 1.56 | 6.62E-21 |
| FRMD8 | 0.62 | 2.47E-02 | 0.71 | 4.22E-02 | 1.64 | 4.71E-21 |
| FSCN1 | 0.58 | 1.32E-03 | 0.51 | 5.72E-05 | 7.77 | 1.24E-67 |
| FTSJ1 | 0.77 | 3.84E-02 | 0.74 | 3.03E-02 | 1.70 | 1.90E-41 |
| FTSJ3 | 0.53 | 1.85E-02 | 0.56 | 1.61E-02 | 1.52 | 1.78E-32 |
| FUBP1 | 0.79 | 1.74E-02 | 0.53 | 1.05E-03 | 1.41 | 9.55E-26 |
| FUS | 0.56 | 7.91E-04 | 0.49 | 2.90E-03 | 1.84 | 1.82E-52 |
| GADD45GIP1 | 0.75 | 5.86E-06 | 0.73 | 2.19E-02 | 1.51 | 1.37E-29 |
| GATAD2A | 0.73 | 1.49E-02 | 0.67 | 1.14E-02 | 1.57 | 8.90E-27 |
| GDF11 | 0.43 | 1.07E-03 | 0.41 | 5.37E-03 | 1.52 | 4.75E-15 |
| GFER | 0.71 | 3.41E-02 | 0.60 | 1.77E-02 | 1.44 | 4.64E-25 |
| GGCX | 0.73 | 2.39E-02 | 0.61 | 5.76E-03 | 1.59 | 3.83E-43 |
| GINS2 | 0.57 | 3.91E-04 | 0.56 | 2.54E-04 | 3.91 | 4.52E-49 |
| GLMP | 0.58 | 1.48E-02 | 0.78 | 3.99E-02 | 1.79 | 4.17E-47 |
| GLRX5 | 0.59 | 1.37E-04 | 0.66 | 5.45E-04 | 1.51 | 8.19E-25 |
| GMNN | 0.59 | 1.50E-04 | 0.70 | 1.90E-02 | 2.80 | 4.45E-53 |
| GPI | 0.75 | 1.61E-03 | 0.63 | 2.04E-02 | 1.45 | 1.30E-16 |
| GPRIN1 | 0.53 | 1.79E-03 | 0.64 | 3.39E-03 | 5.20 | 9.12E-55 |
| GSDMD | 0.72 | 2.60E-02 | 0.66 | 1.74E-02 | 2.04 | 4.20E-40 |
| GTPBP4 | 0.80 | 2.99E-02 | 0.80 | 2.43E-02 | 1.77 | 9.68E-36 |
| HAUS5 | 0.64 | 2.73E-02 | 0.66 | 3.57E-02 | 1.48 | 7.45E-27 |
| HAUS8 | 0.66 | 1.57E-04 | 0.55 | 9.22E-03 | 2.79 | 1.71E-66 |
| HCFC1 | 0.63 | 1.39E-02 | 0.74 | 3.20E-02 | 1.44 | 4.46E-28 |
| HES6 | 0.48 | 3.98E-03 | 0.73 | 4.42E-02 | 2.05 | 7.88E-21 |
| HGH1 | 0.66 | 6.14E-03 | 0.39 | 3.19E-03 | 2.28 | 9.31E-50 |
| HMGA1 | 0.69 | 1.08E-02 | 0.45 | 7.10E-04 | 1.66 | 1.18E-09 |
| HNRNPA2B1 | 0.78 | 4.66E-03 | 0.74 | 2.21E-03 | 1.50 | 6.32E-45 |
| HNRNPM | 0.54 | 2.17E-03 | 0.60 | 5.61E-03 | 1.44 | 6.57E-39 |
| HOXC13 | 0.43 | 5.10E-04 | 0.57 | 6.74E-04 | 13.40 | 1.28E-42 |
| HR | 0.58 | 8.50E-03 | 0.36 | 1.85E-03 | 1.67 | 5.34E-08 |
| HS6ST1 | 0.74 | 4.30E-03 | 0.60 | 3.90E-05 | 1.65 | 1.89E-24 |
| HSPE1 | 0.77 | 1.44E-02 | 0.43 | 1.26E-04 | 2.26 | 4.78E-43 |
| IFT22 | 0.58 | 1.27E-02 | 0.73 | 3.63E-02 | 1.45 | 1.51E-15 |
| INF2 | 0.76 | 6.04E-03 | 0.48 | 1.88E-04 | 1.61 | 1.10E-23 |
| INO80B | 0.59 | 4.41E-03 | 0.27 | 8.05E-03 | 1.41 | 7.57E-22 |
| IPO4 | 0.53 | 3.12E-03 | 0.46 | 1.86E-03 | 1.75 | 4.32E-33 |
| IQCC | 0.60 | 6.03E-05 | 0.33 | 1.46E-03 | 1.42 | 2.90E-24 |
| IQCE | 0.37 | 1.35E-03 | 0.52 | 4.26E-03 | 1.44 | 4.71E-17 |
| IRX2 | 0.53 | 4.21E-04 | 0.18 | 1.12E-05 | 3.21 | 1.99E-20 |
| KCNG2 | 0.29 | 7.11E-03 | 0.25 | 3.17E-02 | 1.44 | 1.67E-10 |
| KHSRP | 0.50 | 7.25E-04 | 0.49 | 1.28E-03 | 1.65 | 7.19E-42 |
| KIF18B | 0.63 | 1.52E-03 | 0.47 | 3.60E-04 | 5.32 | 4.05E-44 |
| KRBA1 | 0.71 | 3.10E-02 | 0.77 | 1.98E-02 | 1.53 | 1.63E-14 |
| KREMEN2 | 0.62 | 1.59E-02 | 0.53 | 1.40E-02 | 7.04 | 3.96E-37 |
| KRT18 | 0.57 | 3.04E-03 | 0.28 | 9.20E-05 | 1.84 | 4.22E-13 |
| KRT8 | 0.56 | 8.90E-04 | 0.24 | 1.71E-04 | 1.90 | 1.56E-08 |
| KTI12 | 0.75 | 1.11E-02 | 0.56 | 3.77E-04 | 1.51 | 1.72E-39 |
| LAD1 | 0.72 | 4.72E-03 | 0.54 | 1.15E-02 | 1.45 | 3.05E-02 |
| LAGE3 | 0.73 | 4.38E-02 | 0.61 | 6.05E-04 | 2.27 | 5.11E-48 |
| LAMTOR4 | 0.71 | 1.80E-02 | 0.58 | 3.95E-03 | 1.65 | 3.31E-27 |
| LASP1 | 0.73 | 7.80E-03 | 0.57 | 4.71E-03 | 1.77 | 2.56E-48 |
| LIMK1 | 0.71 | 7.71E-04 | 0.65 | 1.21E-02 | 3.32 | 2.00E-76 |
| LMNB1 | 0.72 | 1.66E-02 | 0.73 | 6.94E-03 | 2.53 | 9.81E-34 |
| LMNB2 | 0.59 | 1.52E-03 | 0.52 | 4.07E-04 | 2.62 | 1.66E-49 |
| LPCAT1 | 0.65 | 2.37E-02 | 0.38 | 2.05E-03 | 2.97 | 6.29E-51 |
| LPCAT3 | 0.76 | 7.63E-03 | 0.66 | 3.12E-03 | 1.78 | 8.74E-32 |
| LRRC45 | 0.53 | 4.33E-03 | 0.59 | 1.16E-02 | 1.87 | 4.98E-36 |
| LRRC59 | 0.76 | 1.94E-02 | 0.69 | 3.93E-03 | 2.17 | 7.85E-49 |
| LRWD1 | 0.62 | 9.40E-04 | 0.69 | 1.69E-03 | 2.17 | 3.47E-55 |
| LSM10 | 0.60 | 2.41E-04 | 0.39 | 7.18E-03 | 1.44 | 2.89E-24 |
| LSM7 | 0.60 | 4.52E-02 | 0.19 | 4.51E-04 | 1.69 | 1.06E-43 |
| MAGEA4 | 0.79 | 2.43E-03 | 0.66 | 2.39E-04 | 16.96 | 1.72E-27 |
| MAGOH | 0.68 | 5.47E-04 | 0.41 | 3.95E-03 | 1.75 | 3.28E-46 |
| MAP6D1 | 0.26 | 3.35E-04 | 0.48 | 3.63E-03 | 1.84 | 1.45E-29 |
| MARCKSL1 | 0.30 | 7.96E-03 | 0.03 | 1.02E-02 | 4.66 | 1.23E-57 |
| MCM10 | 0.43 | 7.91E-03 | 0.62 | 2.15E-02 | 4.22 | 4.36E-38 |
| MCM2 | 0.69 | 3.64E-04 | 0.68 | 3.24E-04 | 4.41 | 8.29E-60 |
| MCM4 | 0.59 | 2.23E-03 | 0.75 | 4.20E-02 | 3.09 | 1.94E-42 |
| MCM5 | 0.62 | 5.57E-03 | 0.48 | 1.90E-03 | 2.69 | 1.73E-59 |
| MCM6 | 0.62 | 5.93E-03 | 0.71 | 9.15E-03 | 2.25 | 1.15E-54 |
| MCM7 | 0.71 | 1.62E-04 | 0.66 | 1.85E-03 | 2.61 | 2.87E-56 |
| MED10 | 0.58 | 2.99E-03 | 0.39 | 1.81E-03 | 1.74 | 5.20E-43 |
| MEF2B | 0.48 | 5.00E-02 | 0.19 | 1.73E-02 | 1.43 | 2.46E-09 |
| MEN1 | 0.61 | 6.49E-03 | 0.63 | 2.43E-03 | 1.87 | 8.70E-39 |
| METTL1 | 0.71 | 1.05E-02 | 0.53 | 6.08E-03 | 2.23 | 1.37E-46 |
| MLEC | 0.75 | 1.49E-02 | 0.63 | 3.61E-03 | 1.74 | 6.95E-37 |
| MLST8 | 0.79 | 4.43E-02 | 0.77 | 2.53E-02 | 1.51 | 1.42E-40 |
| MMACHC | 0.55 | 1.06E-02 | 0.36 | 3.53E-03 | 1.50 | 5.49E-20 |
| MMP7 | 0.00 | 3.47E-03 | 0.00 | 3.47E-03 | 4.69 | 1.64E-12 |
| MPDU1 | 0.61 | 1.23E-02 | 0.75 | 3.58E-02 | 1.45 | 3.82E-18 |
| MPG | 0.69 | 2.08E-02 | 0.55 | 7.70E-03 | 1.47 | 9.40E-33 |
| MPV17L2 | 0.67 | 1.38E-02 | 0.40 | 4.50E-03 | 1.73 | 3.68E-40 |
| MRM1 | 0.39 | 5.03E-03 | 0.41 | 1.07E-02 | 1.78 | 7.36E-43 |
| MRPL11 | 0.78 | 1.99E-02 | 0.74 | 4.73E-02 | 1.60 | 2.74E-27 |
| MRPL12 | 0.60 | 1.70E-04 | 0.46 | 4.56E-03 | 1.54 | 4.09E-23 |
| MRPL14 | 0.68 | 5.18E-03 | 0.73 | 3.13E-02 | 1.85 | 5.49E-41 |
| MRPL2 | 0.77 | 3.28E-02 | 0.49 | 8.36E-04 | 1.67 | 8.49E-38 |
| MRPL28 | 0.64 | 8.05E-03 | 0.45 | 8.29E-03 | 1.70 | 1.09E-45 |
| MRPL36 | 0.49 | 1.64E-03 | 0.19 | 6.56E-04 | 1.71 | 7.43E-36 |
| MRPL4 | 0.62 | 2.89E-03 | 0.48 | 4.68E-04 | 1.56 | 1.21E-35 |
| MRPS12 | 0.58 | 1.05E-04 | 0.58 | 1.46E-04 | 1.88 | 4.22E-39 |
| MRPS26 | 0.58 | 5.44E-03 | 0.40 | 3.33E-04 | 1.45 | 2.99E-23 |
| MRPS34 | 0.57 | 3.93E-03 | 0.31 | 1.12E-04 | 1.71 | 1.51E-38 |
| MRTO4 | 0.65 | 4.30E-03 | 0.70 | 9.25E-03 | 1.77 | 1.58E-37 |
| MSH6 | 0.66 | 4.93E-03 | 0.75 | 7.57E-03 | 1.97 | 5.67E-45 |
| MTFP1 | 0.49 | 2.91E-04 | 0.48 | 1.18E-03 | 2.13 | 2.16E-34 |
| MTG1 | 0.64 | 1.00E-03 | 0.70 | 3.33E-03 | 1.48 | 8.20E-27 |
| MTG2 | 0.67 | 5.06E-03 | 0.59 | 6.46E-03 | 1.65 | 3.28E-41 |
| MTHFD1 | 0.79 | 3.78E-03 | 0.74 | 1.08E-03 | 1.64 | 1.54E-33 |
| MTX1 | 0.75 | 1.92E-03 | 0.72 | 1.49E-02 | 1.65 | 7.88E-47 |
| MYBBP1A | 0.63 | 2.85E-05 | 0.51 | 3.89E-04 | 1.56 | 1.33E-32 |
| MYBL2 | 0.42 | 9.01E-04 | 0.55 | 1.46E-03 | 10.73 | 8.24E-55 |
| MYDGF | 0.62 | 1.44E-02 | 0.47 | 7.50E-03 | 2.03 | 3.67E-55 |
| NAB2 | 0.78 | 1.97E-02 | 0.54 | 1.21E-03 | 1.46 | 1.19E-16 |
| NABP2 | 0.75 | 3.00E-04 | 0.57 | 2.02E-04 | 1.71 | 6.54E-46 |
| NACC1 | 0.67 | 2.91E-03 | 0.45 | 8.23E-04 | 1.76 | 1.79E-46 |
| NAGPA | 0.46 | 3.14E-03 | 0.56 | 9.66E-03 | 1.41 | 9.63E-28 |
| NASP | 0.75 | 3.51E-05 | 0.60 | 2.39E-03 | 1.73 | 8.78E-37 |
| NAT10 | 0.79 | 1.42E-02 | 0.77 | 1.08E-02 | 1.46 | 2.32E-26 |
| NAXE | 0.70 | 8.80E-04 | 0.80 | 1.51E-02 | 1.80 | 1.20E-43 |
| NCDN | 0.75 | 4.12E-02 | 0.62 | 4.55E-03 | 1.51 | 1.08E-27 |
| NCKAP5L | 0.37 | 6.00E-05 | 0.46 | 1.29E-04 | 1.51 | 5.52E-21 |
| NCLN | 0.59 | 1.86E-02 | 0.66 | 2.80E-02 | 2.28 | 1.98E-65 |
| NDUFS6 | 0.47 | 8.18E-04 | 0.13 | 9.98E-05 | 1.49 | 1.20E-24 |
| NLE1 | 0.53 | 1.16E-03 | 0.47 | 7.15E-04 | 1.51 | 2.88E-27 |
| NME1 | 0.68 | 1.07E-02 | 0.61 | 1.64E-03 | 2.28 | 3.98E-42 |
| NMRAL1 | 0.72 | 2.29E-02 | 0.64 | 1.04E-02 | 1.63 | 8.41E-38 |
| NOC2L | 0.73 | 1.41E-02 | 0.61 | 1.53E-04 | 1.53 | 1.11E-25 |
| NOC4L | 0.69 | 3.40E-02 | 0.52 | 1.01E-02 | 1.70 | 6.76E-45 |
| NOL6 | 0.77 | 3.33E-02 | 0.74 | 2.48E-02 | 1.51 | 3.49E-24 |
| NOP2 | 0.74 | 3.68E-02 | 0.70 | 2.28E-02 | 2.24 | 1.50E-47 |
| NOP56 | 0.73 | 2.07E-02 | 0.68 | 7.27E-03 | 2.08 | 2.03E-50 |
| NPRL3 | 0.80 | 3.18E-02 | 0.68 | 8.03E-03 | 1.53 | 1.27E-36 |
| NR2C2AP | 0.69 | 1.81E-03 | 0.62 | 8.66E-04 | 1.84 | 3.87E-43 |
| NRM | 0.72 | 4.22E-04 | 0.75 | 6.75E-04 | 2.29 | 9.06E-59 |
| NSUN2 | 0.35 | 1.81E-03 | 0.18 | 1.04E-03 | 1.93 | 4.97E-50 |
| NTHL1 | 0.52 | 1.09E-03 | 0.58 | 9.10E-03 | 1.57 | 1.28E-29 |
| NUBP2 | 0.74 | 5.60E-03 | 0.77 | 1.04E-02 | 1.43 | 6.53E-30 |
| NUDC | 0.66 | 1.98E-03 | 0.44 | 4.15E-03 | 1.54 | 2.68E-31 |
| NUDT1 | 0.52 | 1.35E-02 | 0.37 | 6.00E-04 | 2.82 | 1.74E-60 |
| NUP210 | 0.60 | 5.34E-03 | 0.78 | 2.90E-02 | 1.69 | 4.26E-15 |
| NXPH4 | 0.58 | 4.61E-03 | 0.46 | 1.25E-03 | 10.95 | 1.18E-49 |
| ORAI1 | 0.67 | 1.54E-03 | 0.58 | 2.58E-04 | 1.80 | 4.52E-42 |
| ORC1 | 0.54 | 1.25E-03 | 0.45 | 4.00E-03 | 3.77 | 4.14E-34 |
| P2RX5 | 0.69 | 4.20E-03 | 0.77 | 1.78E-02 | 1.49 | 1.59E-06 |
| P3H4 | 0.78 | 9.97E-03 | 0.73 | 3.55E-02 | 4.31 | 9.76E-64 |
| PAQR4 | 0.42 | 9.52E-05 | 0.48 | 5.46E-04 | 3.60 | 6.38E-60 |
| PCNA | 0.60 | 5.88E-04 | 0.70 | 1.80E-03 | 2.51 | 5.10E-52 |
| PDAP1 | 0.75 | 6.28E-03 | 0.50 | 2.34E-02 | 1.61 | 2.93E-32 |
| PDPN | 0.59 | 1.82E-02 | 0.22 | 1.42E-03 | 5.22 | 2.59E-54 |
| PDXP | 0.50 | 9.96E-03 | 0.36 | 6.79E-03 | 1.98 | 7.08E-37 |
| PELP1 | 0.59 | 5.43E-03 | 0.50 | 1.18E-03 | 1.56 | 2.61E-35 |
| PFAS | 0.62 | 2.51E-04 | 0.42 | 2.63E-04 | 1.70 | 4.98E-36 |
| PFDN6 | 0.58 | 2.43E-02 | 0.26 | 1.97E-03 | 1.59 | 6.97E-33 |
| PFN1 | 0.69 | 2.77E-03 | 0.46 | 2.57E-03 | 1.46 | 3.63E-27 |
| PGAM5 | 0.63 | 2.86E-03 | 0.47 | 5.10E-04 | 1.97 | 9.51E-39 |
| PGP | 0.63 | 4.16E-03 | 0.52 | 6.56E-03 | 2.14 | 1.55E-53 |
| PHB2 | 0.70 | 2.56E-04 | 0.59 | 1.96E-03 | 1.48 | 6.04E-24 |
| PIP4K2C | 0.74 | 1.30E-02 | 0.75 | 1.30E-02 | 1.59 | 7.09E-29 |
| PKM | 0.80 | 1.05E-02 | 0.58 | 2.34E-04 | 1.70 | 5.72E-30 |
| PKMYT1 | 0.53 | 5.27E-03 | 0.63 | 1.61E-02 | 7.05 | 8.70E-52 |
| PLEC | 0.78 | 1.59E-02 | 0.57 | 2.79E-03 | 1.57 | 9.34E-18 |
| PLK1 | 0.76 | 5.52E-03 | 0.64 | 1.51E-03 | 5.41 | 5.10E-52 |
| PLPP2 | 0.69 | 2.69E-03 | 0.80 | 2.63E-02 | 2.82 | 4.76E-29 |
| PLXNA1 | 0.74 | 4.51E-03 | 0.62 | 1.97E-03 | 2.98 | 4.48E-65 |
| POC1A | 0.77 | 1.89E-02 | 0.68 | 5.62E-03 | 2.57 | 1.27E-44 |
| POFUT1 | 0.73 | 9.39E-03 | 0.76 | 1.60E-02 | 1.90 | 3.82E-46 |
| POLD1 | 0.68 | 5.60E-03 | 0.79 | 5.35E-03 | 2.49 | 1.23E-60 |
| POLD2 | 0.75 | 3.53E-04 | 0.72 | 4.22E-02 | 1.58 | 1.80E-30 |
| POLD3 | 0.79 | 3.88E-02 | 0.72 | 9.90E-03 | 1.57 | 3.50E-27 |
| POLR2F | 0.63 | 9.50E-03 | 0.36 | 1.36E-03 | 1.50 | 5.55E-32 |
| POLR3K | 0.73 | 2.44E-02 | 0.57 | 3.45E-03 | 1.99 | 2.32E-50 |
| POP1 | 0.60 | 5.17E-03 | 0.56 | 6.51E-03 | 1.73 | 4.41E-27 |
| POP5 | 0.69 | 1.32E-02 | 0.55 | 9.94E-03 | 1.62 | 2.25E-36 |
| POP7 | 0.56 | 1.23E-03 | 0.50 | 7.98E-04 | 1.85 | 6.49E-41 |
| PP7080 | 0.43 | 5.38E-04 | 0.17 | 1.12E-03 | 1.56 | 4.04E-20 |
| PPM1G | 0.62 | 1.48E-04 | 0.59 | 4.68E-03 | 1.65 | 7.46E-46 |
| PPME1 | 0.51 | 1.39E-03 | 0.38 | 1.34E-03 | 1.56 | 1.14E-21 |
| PPP1CA | 0.71 | 3.91E-04 | 0.74 | 1.80E-04 | 1.55 | 1.26E-18 |
| PPP4C | 0.72 | 1.60E-03 | 0.72 | 1.73E-03 | 1.51 | 1.04E-27 |
| PPT1 | 0.80 | 3.23E-03 | 0.63 | 3.43E-04 | 1.99 | 2.69E-44 |
| PRIM1 | 0.79 | 1.09E-02 | 0.73 | 7.16E-04 | 2.25 | 5.37E-40 |
| PRKAR1B | 0.32 | 3.39E-04 | 0.69 | 1.44E-02 | 1.45 | 6.85E-16 |
| PROSER2 | 0.65 | 3.00E-02 | 0.54 | 2.21E-02 | 1.79 | 1.84E-13 |
| PRPF19 | 0.74 | 1.38E-02 | 0.76 | 1.56E-02 | 1.56 | 1.30E-28 |
| PRR19 | 0.52 | 3.12E-02 | 0.42 | 6.76E-03 | 1.97 | 1.03E-28 |
| PRR5 | 0.78 | 1.20E-02 | 0.56 | 1.01E-03 | 2.02 | 6.07E-42 |
| PRR7 | 0.60 | 3.14E-05 | 0.48 | 1.28E-05 | 2.78 | 4.81E-37 |
| PSMB10 | 0.68 | 1.31E-02 | 0.71 | 4.71E-02 | 1.98 | 4.83E-35 |
| PSMB5 | 0.63 | 4.34E-03 | 0.57 | 1.36E-03 | 1.78 | 3.86E-44 |
| PSMD9 | 0.72 | 3.19E-02 | 0.34 | 5.79E-04 | 1.44 | 4.68E-37 |
| PSRC1 | 0.80 | 4.93E-02 | 0.48 | 1.29E-04 | 1.75 | 2.47E-26 |
| PUS1 | 0.62 | 1.44E-02 | 0.51 | 1.19E-02 | 2.09 | 3.73E-52 |
| PWP2 | 0.76 | 2.19E-02 | 0.64 | 3.45E-02 | 1.56 | 7.53E-27 |
| RAD54L | 0.76 | 1.62E-02 | 0.57 | 9.24E-03 | 4.71 | 3.04E-48 |
| RAI1 | 0.65 | 1.22E-02 | 0.63 | 2.45E-03 | 1.80 | 1.17E-40 |
| RANBP1 | 0.72 | 6.71E-03 | 0.65 | 8.49E-04 | 2.41 | 1.98E-57 |
| RAVER1 | 0.58 | 2.12E-03 | 0.48 | 2.07E-03 | 1.74 | 4.62E-43 |
| RBM19 | 0.57 | 1.22E-03 | 0.45 | 4.73E-04 | 1.43 | 2.65E-23 |
| RCC1 | 0.65 | 3.86E-03 | 0.75 | 1.83E-02 | 2.53 | 4.18E-31 |
| RCC1L | 0.64 | 1.97E-03 | 0.58 | 9.76E-04 | 1.46 | 6.16E-26 |
| RCE1 | 0.78 | 1.30E-02 | 0.71 | 5.04E-03 | 2.04 | 2.49E-42 |
| RECQL4 | 0.68 | 3.02E-03 | 0.51 | 1.95E-04 | 3.63 | 3.26E-41 |
| REEP4 | 0.78 | 2.37E-02 | 0.55 | 8.90E-04 | 1.42 | 2.43E-06 |
| RELT | 0.73 | 3.32E-02 | 0.60 | 1.52E-04 | 2.33 | 9.36E-40 |
| RFC5 | 0.75 | 1.63E-03 | 0.50 | 1.24E-04 | 2.05 | 4.72E-48 |
| RIN1 | 0.77 | 2.32E-02 | 0.42 | 2.15E-03 | 1.60 | 4.01E-10 |
| RNASEH2A | 0.74 | 1.25E-03 | 0.50 | 3.10E-05 | 3.43 | 1.14E-59 |
| RPA1 | 0.78 | 2.65E-02 | 0.65 | 3.18E-03 | 1.56 | 5.03E-33 |
| RPP40 | 0.56 | 6.31E-04 | 0.78 | 2.44E-02 | 1.60 | 4.27E-17 |
| RPS6KA1 | 0.75 | 2.06E-03 | 0.65 | 2.79E-02 | 2.50 | 3.68E-42 |
| RPS6KA4 | 0.56 | 4.74E-03 | 0.39 | 9.55E-04 | 1.95 | 1.29E-35 |
| RPUSD1 | 0.46 | 6.65E-03 | 0.61 | 2.32E-02 | 2.10 | 4.78E-57 |
| RRM2 | 0.57 | 1.57E-03 | 0.54 | 1.07E-03 | 3.91 | 6.63E-31 |
| RRP9 | 0.75 | 2.54E-02 | 0.70 | 1.81E-02 | 1.62 | 2.81E-33 |
| RTEL1 | 0.76 | 1.19E-02 | 0.54 | 2.27E-02 | 1.48 | 1.24E-21 |
| RTKN | 0.76 | 8.17E-03 | 0.72 | 1.70E-03 | 2.38 | 7.01E-51 |
| RUVBL1 | 0.79 | 7.46E-03 | 0.62 | 4.37E-03 | 2.64 | 2.04E-59 |
| RUVBL2 | 0.79 | 1.48E-02 | 0.68 | 7.66E-03 | 1.53 | 5.27E-30 |
| S100A2 | 0.59 | 8.77E-03 | 0.12 | 3.10E-03 | 1.90 | 4.56E-04 |
| S1PR5 | 0.74 | 2.00E-02 | 0.49 | 1.42E-02 | 2.67 | 1.70E-19 |
| SAC3D1 | 0.45 | 5.20E-03 | 0.39 | 2.79E-03 | 2.45 | 7.24E-56 |
| SAMD1 | 0.72 | 9.65E-03 | 0.74 | 3.52E-02 | 2.04 | 1.00E-51 |
| SAPCD2 | 0.59 | 1.69E-03 | 0.40 | 2.32E-03 | 3.54 | 6.68E-31 |
| SCAND1 | 0.76 | 6.34E-03 | 0.55 | 1.58E-02 | 1.45 | 1.61E-24 |
| SCLY | 0.64 | 1.98E-02 | 0.62 | 2.56E-02 | 1.46 | 7.87E-20 |
| SDC1 | 0.69 | 7.87E-04 | 0.56 | 4.19E-04 | 1.78 | 1.97E-05 |
| SDF2L1 | 0.59 | 3.01E-03 | 0.55 | 7.05E-03 | 2.02 | 3.94E-45 |
| SDF4 | 0.75 | 1.65E-02 | 0.75 | 2.40E-02 | 1.71 | 1.59E-42 |
| SERPINF2 | 0.27 | 2.85E-05 | 0.62 | 1.08E-02 | 1.47 | 1.32E-12 |
| SF3A2 | 0.62 | 1.39E-02 | 0.36 | 2.01E-03 | 1.54 | 2.34E-35 |
| SF3B4 | 0.71 | 3.42E-02 | 0.53 | 5.41E-03 | 1.72 | 8.65E-48 |
| SFPQ | 0.63 | 2.87E-02 | 0.70 | 4.80E-02 | 1.44 | 1.71E-36 |
| SH2B2 | 0.68 | 1.19E-02 | 0.59 | 4.47E-03 | 1.90 | 1.10E-21 |
| SH3BP1 | 0.72 | 2.61E-03 | 0.43 | 9.31E-03 | 1.65 | 4.65E-11 |
| SH3D21 | 0.67 | 1.65E-02 | 0.45 | 1.76E-04 | 1.74 | 3.74E-13 |
| SHMT2 | 0.75 | 4.67E-05 | 0.38 | 2.69E-04 | 2.27 | 8.53E-42 |
| SIGMAR1 | 0.63 | 6.53E-03 | 0.63 | 5.54E-03 | 1.48 | 3.90E-22 |
| SIVA1 | 0.57 | 2.02E-03 | 0.34 | 9.65E-06 | 1.49 | 7.21E-23 |
| SKP2 | 0.74 | 8.12E-04 | 0.41 | 5.79E-05 | 1.75 | 2.74E-19 |
| SLC10A3 | 0.53 | 2.46E-03 | 0.70 | 1.04E-02 | 1.50 | 3.20E-23 |
| SLC16A3 | 0.38 | 8.47E-03 | 0.58 | 1.35E-02 | 2.32 | 5.35E-33 |
| SLC19A1 | 0.50 | 7.59E-03 | 0.62 | 2.85E-02 | 3.02 | 1.87E-58 |
| SLC25A1 | 0.75 | 3.48E-02 | 0.50 | 3.58E-04 | 1.55 | 5.61E-31 |
| SLC25A10 | 0.51 | 1.42E-04 | 0.48 | 1.92E-03 | 1.40 | 7.90E-07 |
| SLC25A15 | 0.77 | 3.72E-02 | 0.79 | 3.25E-02 | 1.55 | 7.05E-22 |
| SLC25A19 | 0.69 | 7.22E-03 | 0.64 | 3.57E-02 | 1.84 | 1.06E-42 |
| SLC25A39 | 0.80 | 1.63E-02 | 0.60 | 3.78E-03 | 1.50 | 8.57E-22 |
| SLC27A3 | 0.54 | 2.35E-04 | 0.70 | 3.50E-03 | 1.44 | 1.06E-19 |
| SLC35B2 | 0.68 | 4.09E-03 | 0.69 | 8.80E-03 | 2.02 | 4.79E-47 |
| SLC38A7 | 0.49 | 4.60E-04 | 0.64 | 2.51E-02 | 1.98 | 1.03E-50 |
| SLC39A3 | 0.66 | 9.60E-03 | 0.64 | 2.39E-02 | 1.60 | 7.11E-40 |
| SLC7A5 | 0.76 | 3.66E-03 | 0.51 | 1.97E-03 | 2.61 | 3.57E-22 |
| SMARCB1 | 0.75 | 1.75E-03 | 0.73 | 6.09E-03 | 1.73 | 4.08E-41 |
| SMUG1 | 0.77 | 9.99E-03 | 0.64 | 2.33E-03 | 1.41 | 9.02E-30 |
| SNRNP25 | 0.54 | 5.79E-04 | 0.41 | 2.83E-04 | 1.49 | 1.46E-19 |
| SNRNP40 | 0.80 | 1.70E-02 | 0.47 | 2.28E-03 | 1.72 | 2.68E-49 |
| SNRPB | 0.71 | 2.21E-03 | 0.63 | 5.30E-03 | 2.14 | 6.49E-53 |
| SOX15 | 0.43 | 3.34E-04 | 0.21 | 7.64E-04 | 1.47 | 2.70E-05 |
| SPATA33 | 0.39 | 4.53E-03 | 0.51 | 6.40E-03 | 2.01 | 7.51E-51 |
| SPC24 | 0.56 | 8.89E-03 | 0.62 | 1.56E-02 | 3.71 | 9.19E-47 |
| SPIRE2 | 0.63 | 2.44E-02 | 0.63 | 2.96E-04 | 1.40 | 1.18E-07 |
| SPNS1 | 0.61 | 1.48E-02 | 0.62 | 6.29E-03 | 1.86 | 2.70E-55 |
| SPR | 0.62 | 9.99E-04 | 0.58 | 8.19E-04 | 1.85 | 2.05E-38 |
| SRC | 0.80 | 1.98E-02 | 0.78 | 3.06E-02 | 1.46 | 9.21E-22 |
| SREBF1 | 0.69 | 7.29E-03 | 0.47 | 1.88E-03 | 1.81 | 4.70E-29 |
| SRRT | 0.66 | 2.94E-02 | 0.66 | 2.06E-02 | 1.54 | 4.89E-39 |
| SRSF9 | 0.76 | 4.17E-02 | 0.46 | 5.20E-05 | 1.52 | 2.20E-29 |
| SSBP4 | 0.75 | 5.41E-03 | 0.59 | 2.63E-03 | 1.44 | 1.84E-18 |
| SSRP1 | 0.79 | 4.74E-03 | 0.72 | 9.41E-03 | 1.88 | 5.83E-50 |
| STC2 | 0.74 | 4.87E-02 | 0.70 | 3.76E-02 | 5.17 | 2.27E-50 |
| STK32C | 0.69 | 3.38E-02 | 0.60 | 4.37E-02 | 1.60 | 7.19E-23 |
| STMN3 | 0.49 | 7.96E-03 | 0.63 | 4.58E-03 | 1.57 | 6.92E-10 |
| SUV39H1 | 0.73 | 1.16E-02 | 0.67 | 2.70E-04 | 1.75 | 3.66E-33 |
| TACO1 | 0.64 | 4.55E-04 | 0.55 | 1.88E-02 | 1.57 | 3.12E-34 |
| TAF6 | 0.79 | 3.35E-03 | 0.79 | 8.32E-03 | 1.64 | 6.09E-28 |
| TARBP2 | 0.59 | 3.42E-03 | 0.35 | 5.26E-04 | 1.84 | 9.98E-50 |
| TBC1D10B | 0.56 | 4.73E-04 | 0.73 | 1.16E-02 | 1.69 | 9.82E-52 |
| TCF19 | 0.57 | 5.88E-04 | 0.78 | 7.42E-03 | 1.92 | 4.38E-32 |
| TCOF1 | 0.75 | 8.69E-04 | 0.56 | 4.06E-03 | 2.07 | 1.15E-51 |
| TCTN2 | 0.60 | 8.96E-03 | 0.69 | 6.13E-03 | 1.43 | 3.95E-19 |
| TDP1 | 0.79 | 1.86E-02 | 0.73 | 8.76E-03 | 1.78 | 2.60E-35 |
| TEAD4 | 0.70 | 3.06E-03 | 0.67 | 1.75E-02 | 3.11 | 2.24E-50 |
| TELO2 | 0.67 | 2.59E-03 | 0.69 | 3.17E-03 | 1.64 | 4.32E-40 |
| TFPT | 0.44 | 1.01E-02 | 0.49 | 4.49E-02 | 1.43 | 6.80E-22 |
| THOP1 | 0.62 | 1.13E-02 | 0.55 | 3.94E-05 | 1.49 | 5.68E-28 |
| TICRR | 0.78 | 2.45E-02 | 0.79 | 2.57E-02 | 2.25 | 1.28E-37 |
| TIGAR | 0.22 | 3.96E-03 | 0.27 | 5.51E-03 | 1.58 | 2.30E-20 |
| TIGD5 | 0.52 | 3.69E-03 | 0.62 | 9.17E-03 | 2.04 | 2.06E-43 |
| TIMELESS | 0.71 | 1.91E-03 | 0.51 | 4.25E-06 | 3.17 | 2.09E-56 |
| TIMM10 | 0.66 | 4.24E-03 | 0.54 | 2.52E-03 | 1.63 | 1.28E-35 |
| TJAP1 | 0.71 | 1.15E-02 | 0.68 | 3.78E-02 | 1.41 | 6.88E-18 |
| TK1 | 0.44 | 2.61E-04 | 0.60 | 4.08E-03 | 3.79 | 3.70E-65 |
| TLCD1 | 0.71 | 3.80E-03 | 0.66 | 6.50E-03 | 2.08 | 2.75E-20 |
| TMEM102 | 0.54 | 2.13E-03 | 0.68 | 5.53E-03 | 1.71 | 5.64E-15 |
| TMEM160 | 0.57 | 7.96E-03 | 0.52 | 7.61E-03 | 1.44 | 1.66E-29 |
| TMEM161A | 0.71 | 3.37E-02 | 0.64 | 1.93E-02 | 1.75 | 6.94E-45 |
| TMEM199 | 0.47 | 1.26E-02 | 0.57 | 2.91E-02 | 1.45 | 7.49E-27 |
| TMEM208 | 0.68 | 2.72E-02 | 0.67 | 1.76E-02 | 1.93 | 4.19E-56 |
| TMEM223 | 0.64 | 6.02E-05 | 0.77 | 8.03E-04 | 1.82 | 1.70E-41 |
| TMEM39B | 0.77 | 3.23E-02 | 0.70 | 2.37E-02 | 1.58 | 3.95E-47 |
| TMEM70 | 0.70 | 2.87E-02 | 0.67 | 1.33E-02 | 1.42 | 1.71E-16 |
| TNK1 | 0.49 | 3.36E-04 | 0.53 | 4.35E-04 | 1.87 | 2.35E-27 |
| TOMM40 | 0.46 | 1.25E-03 | 0.45 | 1.42E-03 | 1.94 | 1.28E-44 |
| TONSL | 0.60 | 3.09E-03 | 0.44 | 5.97E-03 | 3.97 | 5.79E-58 |
| TOR2A | 0.67 | 9.47E-03 | 0.70 | 1.33E-02 | 1.63 | 1.33E-37 |
| TOR3A | 0.56 | 1.82E-02 | 0.47 | 1.41E-02 | 1.93 | 5.70E-48 |
| TRIP13 | 0.56 | 3.31E-03 | 0.33 | 6.75E-04 | 5.17 | 1.51E-50 |
| TRMT112 | 0.76 | 2.38E-02 | 0.51 | 1.01E-03 | 1.85 | 1.64E-46 |
| TRMT61A | 0.62 | 9.50E-03 | 0.44 | 2.66E-03 | 1.46 | 3.11E-19 |
| TROAP | 0.62 | 1.38E-03 | 0.33 | 2.66E-04 | 5.64 | 4.62E-50 |
| TRPM2 | 0.58 | 1.58E-02 | 0.68 | 3.91E-02 | 2.61 | 1.38E-39 |
| TSFM | 0.69 | 7.68E-03 | 0.54 | 4.19E-03 | 1.65 | 8.99E-38 |
| TSPAN1 | 0.44 | 7.85E-03 | 0.17 | 1.30E-03 | 2.06 | 8.09E-07 |
| TSPAN17 | 0.69 | 6.18E-03 | 0.68 | 5.29E-03 | 1.55 | 8.44E-31 |
| TSR3 | 0.59 | 8.93E-03 | 0.67 | 1.89E-02 | 1.45 | 9.47E-30 |
| TUBB3 | 0.63 | 9.03E-04 | 0.72 | 2.89E-03 | 6.78 | 7.24E-55 |
| TUBG1 | 0.75 | 9.58E-03 | 0.52 | 7.47E-03 | 1.85 | 1.50E-44 |
| TUFM | 0.78 | 1.30E-03 | 0.65 | 5.77E-03 | 1.56 | 3.34E-35 |
| TXNDC5 | 0.74 | 1.61E-02 | 0.71 | 7.87E-03 | 1.54 | 6.35E-21 |
| TYMS | 0.63 | 1.77E-03 | 0.77 | 9.53E-03 | 2.90 | 5.64E-48 |
| U2AF2 | 0.68 | 1.13E-02 | 0.55 | 4.29E-03 | 1.48 | 7.08E-33 |
| UBL4A | 0.62 | 2.18E-03 | 0.79 | 1.44E-02 | 1.53 | 3.72E-28 |
| UBTD1 | 0.74 | 3.61E-02 | 0.62 | 1.91E-02 | 1.98 | 2.36E-39 |
| UHRF1 | 0.46 | 6.18E-05 | 0.51 | 1.82E-04 | 4.86 | 3.97E-45 |
| UMPS | 0.59 | 9.39E-03 | 0.74 | 3.04E-02 | 1.87 | 6.74E-37 |
| UNC93B1 | 0.77 | 3.07E-02 | 0.59 | 1.02E-02 | 1.83 | 3.89E-33 |
| UNG | 0.58 | 9.59E-05 | 0.44 | 4.48E-06 | 1.57 | 2.79E-23 |
| USP5 | 0.77 | 3.58E-03 | 0.78 | 4.72E-03 | 1.41 | 2.06E-19 |
| WDR18 | 0.63 | 1.62E-02 | 0.44 | 2.82E-03 | 1.76 | 1.54E-39 |
| WDR4 | 0.66 | 1.96E-03 | 0.46 | 2.40E-03 | 1.81 | 1.80E-34 |
| WDR46 | 0.69 | 1.47E-03 | 0.70 | 1.71E-03 | 1.56 | 2.54E-37 |
| WDR74 | 0.65 | 8.55E-03 | 0.63 | 9.34E-03 | 1.91 | 1.63E-43 |
| WDR81 | 0.52 | 3.05E-02 | 0.60 | 4.96E-02 | 1.68 | 4.68E-35 |
| WDR90 | 0.62 | 7.80E-03 | 0.60 | 1.07E-02 | 1.91 | 1.99E-38 |
| WRAP53 | 0.48 | 2.21E-03 | 0.44 | 7.91E-04 | 2.28 | 4.51E-54 |
| XXYLT1 | 0.46 | 4.05E-04 | 0.66 | 1.65E-02 | 1.86 | 9.08E-40 |
| XYLT2 | 0.78 | 2.29E-02 | 0.56 | 1.02E-03 | 1.59 | 3.77E-32 |
| YDJC | 0.76 | 1.30E-02 | 0.66 | 5.46E-03 | 2.58 | 9.56E-54 |
| ZC3H18 | 0.64 | 1.54E-04 | 0.72 | 4.07E-03 | 1.45 | 1.04E-29 |
| ZDHHC16 | 0.68 | 9.18E-03 | 0.78 | 2.71E-02 | 1.58 | 1.82E-46 |
| ZDHHC8 | 0.73 | 2.10E-02 | 0.72 | 2.27E-02 | 1.72 | 2.06E-35 |
| ZFP69 | 0.79 | 1.43E-02 | 0.67 | 4.48E-03 | 1.46 | 8.96E-26 |
| ZNF205 | 0.61 | 2.19E-02 | 0.65 | 2.86E-02 | 1.54 | 5.56E-35 |
| ZNF232 | 0.47 | 5.40E-04 | 0.64 | 4.72E-03 | 1.42 | 8.22E-20 |
| ZNF488 | 0.52 | 4.36E-04 | 0.25 | 1.14E-04 | 1.89 | 6.44E-14 |
| ZNF622 | 0.78 | 7.23E-03 | 0.26 | 6.11E-05 | 1.44 | 1.82E-32 |
| ZNF707 | 0.77 | 9.58E-04 | 0.64 | 1.40E-02 | 2.07 | 2.69E-52 |
| ZNHIT2 | 0.58 | 1.00E-02 | 0.55 | 9.92E-03 | 1.63 | 7.68E-31 |
| ZWINT | 0.58 | 1.84E-02 | 0.53 | 1.58E-02 | 3.08 | 9.26E-34 |

**Supplementary Table 5. Results of immunohistochemical staining of TIGAR in ESCC**

| Sample ID | Normal | | | ESCC | | |
| --- | --- | --- | --- | --- | --- | --- |
|  | Positive | Intensity | IRS^*^ | Positive | Intensity | IRS^*^ |
| 150115 | 2 | 2 | 4 | 2 | 2 | 4 |
| 150146 | 2 | 2 | 4 | 1 | 4 | 4 |
| 150159 | 1 | 2 | 2 | 2 | 4 | 8 |
| 150201 | 2 | 2 | 4 | 2 | 2 | 4 |
| 150203 | 3 | 2 | 6 | 2 | 3 | 6 |
| 150208 | 2 | 3 | 6 | 2 | 4 | 8 |
| 150219 | 1 | 3 | 3 | 3 | 3 | 9 |
| 150228 | 1 | 3 | 3 | 2 | 3 | 6 |
| 150229 | 2 | 3 | 6 | 1 | 4 | 4 |
| 150234 | 1 | 1 | 1 | 1 | 3 | 3 |
| 150236 | 2 | 2 | 4 | 2 | 3 | 6 |
| 150241 | 2 | 3 | 6 | 2 | 3 | 6 |
| 150302 | 2 | 3 | 6 | 2 | 4 | 8 |
| 150304 | 2 | 3 | 6 | 3 | 3 | 9 |
| 150312 | 1 | 1 | 1 | 2 | 4 | 8 |
| 150313 | 2 | 2 | 4 | 2 | 3 | 6 |
| 150319 | 2 | 3 | 6 | 3 | 3 | 9 |
| 150323 | 2 | 2 | 4 | 2 | 3 | 6 |
| 150324 | 3 | 3 | 9 | 3 | 2 | 6 |
| 150331 | 2 | 3 | 6 | 2 | 4 | 8 |
| 150333 | 2 | 3 | 6 | 2 | 4 | 8 |
| 150334 | 1 | 3 | 3 | 2 | 3 | 6 |
| 150348 | 1 | 2 | 2 | 1 | 4 | 4 |
| 150355 | 1 | 2 | 2 | 2 | 4 | 8 |
| 150374 | 1 | 3 | 3 | 2 | 3 | 6 |
| 150378 | 2 | 3 | 6 | 2 | 3 | 6 |
| 150385 | 1 | 1 | 1 | 2 | 3 | 6 |
| 150388 | 1 | 1 | 1 | 3 | 3 | 9 |
| 150395 | 1 | 2 | 2 | 2 | 3 | 6 |
| 150411 | 3 | 2 | 6 | 1 | 4 | 4 |
| 150414 | 3 | 2 | 6 | 3 | 3 | 9 |
| 150418 | 1 | 3 | 3 | 2 | 4 | 8 |
| 150423 | 2 | 3 | 6 | 3 | 3 | 9 |
| 150429 | 2 | 3 | 6 | 2 | 4 | 8 |
| 150431 | 2 | 3 | 6 | 2 | 3 | 6 |
| 150432 | 2 | 3 | 6 | 2 | 3 | 6 |
| 150454 | 2 | 3 | 6 | 2 | 4 | 8 |
| 150455 | 2 | 2 | 4 | 2 | 4 | 8 |
| 150458 | 1 | 3 | 3 | 1 | 4 | 4 |
| 150463 | 1 | 1 | 1 | 1 | 2 | 2 |
| 150468 | 1 | 1 | 1 | 1 | 4 | 4 |
| 150471 | 1 | 1 | 1 | 2 | 3 | 6 |
| 150472 | 0 | 0 | 0 | 1 | 4 | 4 |
| 150476 | 1 | 2 | 2 | 1 | 3 | 3 |
| 150481 | 1 | 1 | 1 | 2 | 3 | 6 |
| 150502 | 1 | 2 | 2 | 3 | 3 | 9 |
| 150503 | 1 | 2 | 2 | 2 | 4 | 8 |
| 150506 | 1 | 1 | 1 | 2 | 4 | 8 |
| 150510 | 1 | 1 | 1 | 1 | 4 | 4 |
| 150513 | 1 | 3 | 3 | 3 | 4 | 12 |
| 150526 | 2 | 2 | 4 | 2 | 4 | 8 |
| 150527 | 2 | 2 | 4 | 2 | 4 | 8 |
| 150528 | 1 | 1 | 1 | 3 | 3 | 9 |
| 150531 | 1 | 1 | 1 | 1 | 4 | 4 |
| 150543 | 1 | 2 | 2 | 3 | 3 | 9 |
| 150544 | 1 | 1 | 1 | 3 | 3 | 9 |
| 150554 | 1 | 2 | 2 | 3 | 3 | 9 |
| 150555 | 2 | 2 | 4 | 2 | 3 | 6 |
| 150557 | 1 | 3 | 3 | 1 | 4 | 4 |
| 150560 | 1 | 3 | 3 | 1 | 4 | 4 |
| 150561 | 2 | 2 | 4 | 2 | 4 | 8 |
| 150573 | 1 | 3 | 3 | 2 | 3 | 6 |
| 150582 | 1 | 3 | 3 | 2 | 4 | 8 |
| 150601 | 1 | 3 | 3 | 1 | 4 | 4 |
| 150603 | 1 | 4 | 4 | 3 | 3 | 9 |
| 150606 | 2 | 2 | 4 | 1 | 4 | 4 |
| 150609 | 1 | 4 | 4 | 2 | 3 | 6 |
| 150613 | 1 | 2 | 2 | 2 | 3 | 6 |
| 150614 | 1 | 2 | 2 | 3 | 3 | 9 |
| 150617 | 2 | 2 | 4 | 2 | 4 | 8 |
| 150618 | 2 | 3 | 6 | 1 | 4 | 4 |
| 150620 | 2 | 2 | 4 | 2 | 4 | 8 |
| 150622 | 1 | 2 | 2 | 2 | 4 | 8 |
| 150623 | 1 | 1 | 1 | 3 | 3 | 9 |
| 150627 | 1 | 3 | 3 | 2 | 4 | 8 |
| 150631 | 1 | 1 | 1 | 2 | 3 | 6 |
| 150641 | 2 | 2 | 4 | 3 | 3 | 9 |
| 150645 | 1 | 3 | 3 | 2 | 3 | 6 |
| 150654 | 1 | 3 | 3 | 2 | 2 | 4 |
| 150660 | 1 | 3 | 3 | 3 | 2 | 6 |
| 150662 | 2 | 3 | 6 | 3 | 2 | 6 |
| 150664 | 2 | 3 | 6 | 2 | 3 | 6 |
| 150666 | 1 | 3 | 3 | 2 | 4 | 8 |
| 150667 | 2 | 2 | 4 | 3 | 3 | 9 |
| 150671 | 1 | 3 | 3 | 3 | 4 | 12 |
| 150703 | 2 | 3 | 6 | 2 | 3 | 6 |
| 150707 | 2 | 3 | 6 | 3 | 4 | 12 |
| 150710 | 2 | 3 | 6 | 2 | 3 | 6 |
| 150724 | 1 | 3 | 3 | 2 | 4 | 8 |
| 150726 | 2 | 2 | 4 | 2 | 4 | 8 |
| 150735 | 1 | 1 | 1 | 2 | 4 | 8 |
| 150736 | 1 | 2 | 2 | 2 | 3 | 6 |
| 150739 | 1 | 2 | 2 | 3 | 3 | 9 |
| 150741 | 1 | 4 | 4 | 2 | 4 | 8 |
| 150745 | 2 | 3 | 6 | 2 | 3 | 6 |
| 150747 | 1 | 3 | 3 | 1 | 3 | 3 |
| 150749 | 1 | 2 | 2 | 2 | 2 | 4 |
| 150762 | 3 | 2 | 6 | 3 | 3 | 9 |
| 150770 | 1 | 3 | 3 | 2 | 4 | 8 |
| 150805 | 1 | 2 | 2 | 2 | 3 | 6 |
| 150812 | 1 | 3 | 3 | 3 | 3 | 9 |
| 150813 | 2 | 2 | 4 | 3 | 3 | 9 |
| 150843 | 3 | 2 | 6 | 2 | 4 | 8 |
| 150845 | 2 | 3 | 6 | 2 | 3 | 6 |
| 150846 | 2 | 2 | 4 | 2 | 4 | 8 |
| 150848 | 0 | 0 | 0 | 1 | 4 | 4 |
| 150854 | 2 | 2 | 4 | 2 | 3 | 6 |
| 150855 | 2 | 3 | 6 | 2 | 4 | 8 |
| 150901 | 2 | 2 | 4 | 2 | 4 | 8 |
| 150903 | 2 | 2 | 4 | 2 | 3 | 6 |
| 150906 | 2 | 2 | 4 | 3 | 4 | 12 |
| 150909 | 2 | 3 | 6 | 2 | 4 | 8 |
| 150914 | 2 | 3 | 6 | 3 | 4 | 12 |
| 150918 | 1 | 3 | 3 | 2 | 3 | 6 |
| 150928 | 2 | 3 | 6 | 3 | 4 | 12 |
| 150933 | 2 | 2 | 4 | 3 | 3 | 9 |
| 150934 | 2 | 3 | 6 | 2 | 3 | 6 |
| 150936 | 1 | 3 | 3 | 2 | 4 | 8 |
| 150947 | 2 | 2 | 4 | 2 | 3 | 6 |
| 150953 | 3 | 2 | 6 | 2 | 4 | 8 |
| 150954 | 2 | 2 | 4 | 1 | 4 | 4 |
| 151002 | 2 | 2 | 4 | 3 | 4 | 12 |
| 151011 | 1 | 2 | 2 | 3 | 4 | 12 |
| 151012 | 1 | 2 | 2 | 2 | 2 | 4 |
| 151016 | 1 | 2 | 2 | 1 | 4 | 4 |
| 151017 | 1 | 2 | 2 | 1 | 4 | 4 |
| 151020 | 2 | 3 | 6 | 2 | 3 | 6 |
| 151021 | 1 | 3 | 3 | 2 | 4 | 8 |
| 151024 | 1 | 3 | 3 | 2 | 3 | 6 |
| 151033 | 1 | 3 | 3 | 2 | 2 | 4 |
| 151040 | 1 | 3 | 1 | 1 | 4 | 4 |
| 151044 | 2 | 3 | 6 | 3 | 3 | 9 |
| 151047 | 1 | 4 | 4 | 2 | 3 | 6 |
| 151049 | 1 | 3 | 3 | 1 | 4 | 4 |
| 151050 | 2 | 3 | 6 | 2 | 3 | 6 |
| 151052 | 1 | 4 | 4 | 2 | 4 | 8 |
| 151053 | 2 | 3 | 6 | 2 | 3 | 6 |
| 151107 | 1 | 3 | 3 | 2 | 3 | 6 |
| 160105 | 1 | 3 | 3 | 2 | 3 | 6 |
| 160106 | 1 | 2 | 2 | 2 | 3 | 6 |
| 160110 | 1 | 3 | 3 | 2 | 3 | 6 |
| 160111 | 2 | 3 | 6 | 2 | 4 | 8 |
| 160114 | 1 | 4 | 4 | 2 | 3 | 6 |
| 160117 | 1 | 1 | 1 | 2 | 3 | 6 |
| 160119 | 2 | 2 | 4 | 2 | 2 | 4 |
| 160139 | 2 | 2 | 4 | 2 | 3 | 6 |
| 160140 | 1 | 3 | 3 | 3 | 3 | 9 |
| 160143 | 1 | 3 | 3 | 2 | 3 | 6 |
| 160161 | 2 | 3 | 6 | 2 | 4 | 8 |
| 160204 | 2 | 3 | 6 | 2 | 3 | 6 |
| 160209 | 1 | 3 | 3 | 1 | 4 | 4 |
| 160211 | 2 | 3 | 6 | 2 | 4 | 8 |
| 160218 | 2 | 3 | 6 | 2 | 3 | 6 |
| 160219 | 2 | 2 | 4 | 2 | 3 | 6 |
| 160222 | 1 | 3 | 3 | 2 | 4 | 8 |
| 160224 | 3 | 2 | 6 | 2 | 2 | 4 |
| 160227 | 2 | 3 | 6 | 2 | 3 | 6 |
| 160228 | 2 | 3 | 6 | 2 | 3 | 6 |
| 160229 | 2 | 2 | 4 | 3 | 3 | 9 |
| 160236 | 1 | 4 | 4 | 3 | 3 | 9 |
| 160238 | 2 | 3 | 6 | 3 | 3 | 9 |
| 160246 | 2 | 3 | 6 | 1 | 4 | 4 |
| 160248 | 1 | 3 | 3 | 2 | 2 | 4 |
| 160301 | 1 | 3 | 3 | 3 | 3 | 9 |
| 160303 | 1 | 4 | 4 | 2 | 4 | 8 |
| 160309 | 1 | 1 | 1 | 1 | 4 | 4 |
| 160310 | 1 | 3 | 3 | 2 | 4 | 8 |
| 160311 | 2 | 3 | 6 | 2 | 4 | 8 |
| 160319 | 3 | 3 | 9 | 3 | 3 | 9 |
| 160320 | 2 | 3 | 6 | 1 | 4 | 4 |
| 160324 | 1 | 3 | 3 | 2 | 4 | 8 |
| 160326 | 1 | 2 | 2 | 1 | 4 | 1 |
| 160333 | 1 | 4 | 4 | 2 | 4 | 8 |
| 160334 | 2 | 3 | 6 | 2 | 3 | 6 |
| 160342 | 2 | 4 | 8 | 3 | 4 | 12 |
| 160343 | 2 | 3 | 6 | 2 | 4 | 6 |
| 160351 | 2 | 2 | 4 | 2 | 4 | 8 |
| 160362 | 1 | 3 | 3 | 2 | 4 | 8 |
| 160365 | 2 | 4 | 8 | 3 | 4 | 12 |
| 160372 | 1 | 1 | 1 | 2 | 3 | 6 |
| 160375 | 1 | 3 | 3 | 2 | 3 | 6 |
| 160411 | 1 | 3 | 3 | 2 | 4 | 8 |
| 160413 | 1 | 3 | 3 | 2 | 4 | 8 |
| 160414 | 2 | 3 | 6 | 2 | 3 | 6 |
| 160432 | 1 | 3 | 3 | 2 | 4 | 8 |
| 160439 | 1 | 3 | 3 | 2 | 4 | 8 |
| 160441 | 2 | 3 | 6 | 1 | 4 | 4 |
| 160443 | 2 | 3 | 6 | 2 | 4 | 8 |
| 160458 | 2 | 3 | 6 | 2 | 4 | 8 |
| 160460 | 1 | 3 | 3 | 2 | 3 | 6 |
| 160461 | 2 | 3 | 6 | 2 | 4 | 8 |
| 160466 | 3 | 2 | 6 | 2 | 3 | 6 |
| 160467 | 1 | 3 | 3 | 2 | 4 | 8 |
| 160473 | 1 | 3 | 3 | 2 | 3 | 6 |
| 160474 | 1 | 3 | 3 | 2 | 3 | 6 |
| 1503100 | 2 | 2 | 4 | 2 | 4 | 8 |
| 1503101 | 1 | 1 | 1 | 2 | 3 | 6 |

^*^IRS, immune reactive score. IRS was obtained by multiplying the score of positive and that of intensity. The labeling score of positive was defined as 1 (≤10%), 2 (11%⎯50%), 3 (51%⎯80%) and 4 (>80%); the labeling score of intensity was estimated as negative (0), weak (1), moderate (2) and strong (3).

**Supplementary Table 6. Characteristics of 140 patients with ESCC accepted radiochemotherapy before surgery in this study**

| Sample ID | Sex^a^ | Age (year) | TNM stage^b^ | Radiotherapy^c^ | Radiation dose^d^ | Chemotherapy^e^ | Response^f^ |
| --- | --- | --- | --- | --- | --- | --- | --- |
| 1241001 | M | 66 | III | IMRT | 49.91 Gy/23F | PT | PR |
| 1269077 | M | 66 | III | IMRT | 49.22 Gy/23F | PT | PR |
| 1283732 | M | 61 | III | IMRT | 49.22 Gy/23F | PT | PR |
| 1291743 | M | 59 | IV | IMRT | 46.00 Gy/23F | PT | PR |
| 1305488 | M | 75 | III | IMRT | 49.22 Gy/23F | F | PR |
| 1305619 | F | 72 | III | IMRT | 40.00 Gy/20F | PF | SD |
| 1326566 | M | 68 | III | IMRT | 42.00 Gy/20F | PT | PR |
| 1358932 | M | 71 | III | VMAT | 47.08 Gy/22F | PT | PR |
| 1396549 | M | 50 | III | VMAT | 49.22 Gy/23F | PT | PR |
| 1262553 | M | 50 | III | IMRT | 49.22 Gy/23F | PT | SD |
| 1270848 | M | 58 | III | IMRT | 49.91 Gy/23F | PT | PR |
| 1277902 | M | 75 | III | IMRT | 40.00 Gy/20F | PT | PR |
| 1287216 | M | 55 | III | IMRT | 42.00 Gy/21F | PT | PR |
| 1316077 | M | 63 | III | IMRT | 40.00 Gy/20F | PT | SD |
| 1392586 | M | 69 | III | VAMT | 47.08 Gy/22F | PT | SD |
| 1315047 | M | 71 | II | VAMT | 40.00 Gy/20F | PT | PR |
| 829339 | M | 49 | IV | IMRT | 60Gy/30F | PT | PD |
| 848787 | M | 50 | II | IMRT | 60Gy/30F | PF | PR |
| 851394 | M | 69 | IV | IMRT | 60Gy/30F |  | PR |
| 851959 | M | 51 | IV | IMRT | 40Gy/20F | PT | PR |
| 852034 | M | 63 | III | IMRT | 60Gy/30F | PT | PR |
| 861386 | F | 73 | II | IMRT | 60Gy/30F |  | PR |
| 861437 | M | 68 | III | IMRT | 50Gy/25F |  | PR |
| 890728 | M | 49 | IV | IMRT | 60Gy/30F | PT | PR |
| 904171 | M | 67 | III | IMRT | 60Gy/30F |  | PR |
| 905424 | M | 67 | III | IMRT | 60Gy/30F |  | PR |
| 908758 | F | 60 | III | IMRT | 58Gy/29F |  | PR |
| 909098 | M | 55 | III | IMRT | 60Gy/30F | PE | SD |
| 917797 | M | 64 | III | IMRT | 54Gy/30F | PT | PR |
| 917883 | M | 67 | III | IMRT | 60Gy/30F | PT | PR |
| 917962 | M | 66 | III | IMRT | 40Gy/20F | PT | PR |
| 919556 | M | 68 | IV | IMRT | 59.4Gy/33F | PT | PR |
| 922066 | F | 64 | III | IMRT | 40Gy/20F | PT | PR |
| 923653 | M | 58 | III | IMRT | 50Gy/25F | PT | PR |
| 925527 | M | 64 | III | IMRT | 40Gy/20F | PT | PR |
| 933186 | M | 50 | III | IMRT | 50Gy/25F | PT | PR |
| 968596 | M | 70 | III | IMRT | 60Gy/30F | PT | PR |
| 971165 | F | 70 | III | IMRT | 60Gy/30F | PT | PR |
| 971562 | F | 56 | III | IMRT | 50Gy/25F | PT | PR |
| 973311 | F | 79 | III | IMRT | 54Gy/30F |  | PR |
| 997449 | M | 69 | IV | IMRT | 60Gy/30F | PT | SD |
| 1004956 | M | 49 | IV | IMRT | 60Gy/30F |  | PR |
| 1005305 | M | 76 | III | IMRT | 56Gy/28F |  | PR |
| 1007322 | M | 50 | III | IMRT | 40Gy/20F | PT | SD |
| 1025680 | M | 56 | II | IMRT | 46Gy/23F | PT | PR |
| 1027641 | M | 53 | III | IMRT | 46Gy/23F | PT | PR |
| 1032016 | M | 70 | III | IMRT | 60Gy/30F | PT | PR |
| 1038380 | M | 72 | III | IMRT | 50.5Gy/28F | C | SD |
| 1042150 | M | 55 | III | IMRT | 45Gy/25F | PT | PR |
| 1043135 | M | 61 | IV | IMRT | 60Gy/30F | PT | PR |
| 1129962 | M | 59 | II | IMRT | 50.4Gy/28F | PT | SD |
| 1137777 | M | 80 | IV | IMRT | 50Gy/25F |  | SD |
| 1141860 | M | 45 | II | IMRT | 59.92Gy/28F | PT | PR |
| 1147714 | M | 80 | III | IMRT | 50.4Gy/28F |  | SD |
| 1148084 | M | 59 | III | IMRT | 50.4Gy/28F |  | PR |
| 1154780 | M | 74 | II | IMRT | 53.5Gy/25F |  | SD |
| 1156102 | M | 49 | III | IMRT | 46Gy/23F | PT | SD |
| 1157397 | M | 47 | III | IMRT | 49.22Gy/23F | PT | PR |
| 1157687 | M | 59 | IV | IMRT | 50.4Gy/28F | PT | PR |
| 1157916 | M | 51 | III | IMRT | 59.92Gy/28F | PT | SD |
| 1159681 | M | 69 | III | IMRT | 59.92Gy/28F |  | PR |
| 1164307 | M | 38 | III | IMRT | 60Gy/30F | PT | SD |
| 1166802 | M | 84 | IV | IMRT | 54Gy/30F |  | PR |
| 1167543 | M | 57 | IV | IMRT | 59.92Gy/28F | PT | PR |
| 1169968 | M | 66 | II | IMRT | 54Gy/7F | PT | PD |
| 1174356 | M | 77 | II | IMRT | 56Gy/28F |  | PD |
| 1177213 | M | 58 | IV | IMRT | 59.92Gy/28F | PT | PR |
| 1179533 | M | 69 | III | IMRT | 60Gy/30F |  | SD |
| 1179975 | M | 53 | IV | IMRT | 60Gy/10F |  | PD |
| 1180446 | F | 76 | IV | IMRT | 50Gy/25F |  | PR |
| 1180891 | M | 71 | III | IMRT | 49.22Gy/23F | PT | PR |
| 1182784 | M | 51 | III | IMRT | 59.92Gy/28F | PT | PR |
| 1183446 | F | 74 | III | IMRT | 59.92Gy/28F |  | PR |
| 1184990 | M | 44 | III | IMRT | 39.6Gy/22F | PT | PR |
| 1185686 | M | 64 | II | IMRT | 59.92Gy/28F | PT | PR |
| 1188271 | M | 76 | III | IMRT | 59.92Gy/28F |  | PR |
| 1189826 | F | 65 | III | IMRT | 60Gy/30F |  | SD |
| 1197122 | M | 59 | III | IMRT | 59.92Gy/28F | PT | SD |
| 1197174 | M | 63 | III | IMRT | 59.92Gy/28F | PT | PR |
| 1198329 | M | 52 | III | IMRT | 46Gy/23F | PT | PR |
| 1200101 | F | 65 | III | IMRT | 59.92Gy/28F |  | PR |
| 1211991 | M | 65 | IV | IMRT | 59.92Gy/28F | PT | SD |
| 1214369 | M | 54 | III | IMRT | 69.96Gy/33F |  | PR |
| 1224252 | M | 58 | III | IMRT | 60.76Gy/28F | PT | PR |
| 1226099 | M | 48 | III | IMRT | 50.4Gy/33F |  | SD |
| 1226818 | M | 60 | II | IMRT | 49.91Gy/23F | PT | PR |
| 1227815 | M | 56 | III | VMAT | 50.4Gy/28F | PT | PR |
| 1228303 | M | 67 | IV | IMRT | 60.76Gy/28F | PT | PR |
| 1229607 | M | 60 | III | IMRT | 61.88Gy/28F | PT | SD |
| 1230061 | F | 81 | III | IMRT | 60Gy/30F |  | PR |
| 1230667 | M | 50 | III | IMRT | 64.12Gy/28F | PT | SD |
| 1231090 | M | 47 | III | VMAT | 54Gy/27F | PT | SD |
| 1234543 | M | 57 | III | IMRT | 64.12Gy/28F | PT | PR |
| 1242723 | M | 64 | III | IMRT | 55.25Gy/25F | PT | PR |
| 1261323 | M | 59 | III | IMRT | 59.92Gy/28F |  | SD |
| 1263814 | M | 72 | II | IMRT | 59.92Gy/28F |  | PR |
| 1264836 | M | 60 | II | IMRT | 59.92Gy/28F | PT | PR |
| 1268115 | M | 66 | II | IMRT | 59.92Gy/28F | PT | PR |
| 1270219 | M | 83 | II | IMRT | 60Gy/30F |  | PR |
| 1302953 | M | 69 | IV | VMAT | 59.92Gy/28F |  | PR |
| 1307851 | M | 66 | III | IMRT | 30Gy/15F |  | PD |
| 1311348 | M | 59 | III | IMRT | 59.92Gy/28F | PT | SD |
| 1312118 | M | 65 | IV | IMRT | 59.92Gy/28F |  | SD |
| 1313026 | M | 48 | IV | IMRT | 59.92Gy/28F |  | SD |
| 1313835 | M | 50 | III | IMRT | 59.92Gy/28F | PT | SD |
| 1314524 | M | 56 | III | IMRT | 59.92Gy/28F | PT | PR |
| 1315065 | F | 61 | III | IMRT | 59.92Gy/28F |  | SD |
| 1317232 | F | 70 | IV | IMRT | 59.92Gy/28F | F | PR |
| 1318205 | M | 58 | III | IMRT | 49.22Gy/23F |  | PR |
| 1318592 | M | 63 | IV | IMRT | 59.92Gy/28F | PT | PR |
| 1319469 | M | 80 | II | IMRT | 59.92Gy/28F |  | PR |
| 1320441 | M | 47 | III | IMRT | 59.92Gy/28F | PT | PR |
| 1320890 | M | 46 | III | IMRT | 49.22Gy/23F |  | SD |
| 1324987 | M | 74 | IV | IMRT | 59.92Gy/28F | PE | PR |
| 1325148 | M | 70 | III | IMRT | 53.5Gy/25F | F | SD |
| 1325887 | M | 53 | III | IMRT | 59.92Gy/28F | PT | PR |
| 1326417 | M | 62 | III | IMRT | 59.92Gy/28F |  | SD |
| 1326666 | M | 78 | III | IMRT | 59.92Gy/28F | F | PR |
| 1329293 | M | 59 | III | IMRT | 59.92Gy/28F | PT | PR |
| 1331506 | M | 47 | III | IMRT | 59.92Gy/28F | F | PR |
| 1333293 | M | 49 | IV | IMRT | 53.5Gy/25F | F | PR |
| 1338023 | M | 51 | III | VMAT | 59.92Gy/28F |  | SD |
| 1338843 | M | 58 | III | IMRT | 49.22Gy/23F | PT | PR |
| 1339383 | M | 50 | III | IMRT | 49.22Gy/23F | PT | PR |
| 1347455 | M | 49 | III | IMRT | 49.22Gy/23F | PT | SD |
| 1349354 | F | 82 | III | IMRT | 28Gy/14F | F | SD |
| 1349781 | M | 56 | III | IMRT | 59.92Gy/28F | F | PR |
| 1352606 | M | 57 | IV | VMAT | 59.92Gy/28F | PT | PR |
| 1353013 | M | 77 | III | IMRT | 51.36Gy/24F | F | PR |
| 1353525 | M | 72 | III | VMAT | 55.64Gy/26F | F | PR |
| 1353584 | M | 58 | III | VAMT | 50.4Gy/28F | PT | PD |
| 1354535 | M | 60 | IV | VMAT | 59.4Gy/33F | PT | PR |
| 1356795 | M | 64 | III | VMAT | 59.92Gy/28F | F | PR |
| 1359797 | M | 59 | III | VMAT | 59.92Gy/28F | PT | PD |
| 1361706 | F | 53 | IV | IMRT | 53.5Gy/25F | PT | SD |
| 1362063 | M | 70 | II | VMAT | 59.92Gy/28F | F | PR |
| 1362820 | M | 73 | IV | VMAT | 53.5Gy/25F | F | PR |
| 1362847 | F | 59 | II | VMAT | 50.4Gy/28F | PT | SD |
| 1363038 | M | 46 | III | VMAT | 59.92Gy/28F | PT | PR |
| 1365332 | M | 54 | II | VMAT | 59.92Gy/28F | PT | SD |

^a^M, male; F, female.

^b^Tumor TNM staging components including tumor (T), lymph node (N) and metastasis (M) were reviewed by 3 pathologists and defined according to the American Joint Committee on Cancer (AJCC) 7th edition.

^c^IMRT, Intensity-modulated radiation therapy; VMAT, Volumetric modulated arc therapy.

^d^F, fraction.

^e^P, platinum drug; T, paclitaxel; F, 5'Fluorouracil; C, capecitabine. E, Etoposide.

^f^According to the RECIST guidelines for solid tumors, the response criteria were evaluated as partial response (PR), complete response (CR), progressive disease (PD) and stable disease (SD).

**Supplementary Table 7. The primers, sgRNAs and siRNAs used in this study**

| For qRT-PCR | Primer sequence (5' → 3') | |
| --- | --- | --- |
| GDPDH-F | GTCTCCTCTGACTTCAACAGCG | |
| GDPDH-R | ACCACCCTGTTGCTGTAGCCAA | |
| ACTB-F | CATTGCTGACAGGATGCAGAAGG | |
| ACTB-R | TGCTGGAAGGTGGACAGTGAGG | |
| NSUN2-F | GAACTTGCCTGGCACACAAAT | |
| NSUN2-R | TGCTAACAGCTTCTTGACGACTA | |
| TIGAR-F | GGGAAGAGTGCCCTGTGTTT | |
| TIGAR-R | AATCCTGGAATACCGCTGTC | |
| INO80B-F | CCTCTCCACTTCGGGACCTA | |
| INO80B-R | GCAGCATAGGGGAAGGTTGA | |
| EXO5-F | GCACTATACAGCCAAGGGGG | |
| EXO5-R | TGGATTAGGCTAGCAGGGGT | |
| NUDT1-F | CCTGGGCATGAAAAAGCGAG | |
| NUDT1-R | TCAAACACGATCTGGCCCAC | |
| TRIP13-F | GCAGCAAATCACTGGGTTCTACC | |
| TRIP13-R | GTTCCAGGTGATGAGGTTGCTG | |
| WRAP53-F | GGAGACACCGCTTGGAACTAC | |
| WRAP53-R | CCCACTTACAGCCTTTCAAGAA | |
| TFPT-F | CAGGAAATGCGCTGACTCCAGA | |
| TFPT-R | GTAGGGCAGCAGTTTGTCTGGA | |
| UHRF1-F | ATGGGGGTTTTTGCTGTCCC | |
| UHRF1-R | GTCCTCCATCTGTTTGCCCC | |
| FEN1-F | ACTAAGCGGCTGGTGAAGGTCA | |
| FEN1-R | GCAGCATAGACTTTGCCAGCCT | |
| UNG-F | CCCCACACCAAGTCTTCACC | |
| UNG-R | TTGAACACTAAAGCAGAGCCC | |
| LSM7-F | CGACAAGACGATCCGGGTAAA | |
| LSM7-R | GGTTGAGGAGTGGGTCGAAG | |
| MRM1-F | TCTGGGAACGGCCTACTCTC | |
| MRM1-R | TGGCTGCAAATGGAGTGAAGA | |
| SNRNP25-F | TCTGCCGATCCAGGTTACTC | |
| SNRNP25-R | GGCACTCTGCACTACAACCA | |
| SF3A2-F | GGGAAGAAGCACCAGACCAA | |
| SF3A2-R | GCTTGGTCACTTTGTAGCCC | |
| KHSRP-F | TTACAAAAAGATCGGCCAGCA | |
| KHSRP-R | CACTTGCGCTTGCTTCTTGT | |
| LSM10-F | GTTGGGGGCATCAGGTCAAG | |
| LSM10-R | CTGCTCAATGGTCGAGGTGA | |
| FUS-F | GCCAGAACACAGGCTATGGAA | |
| FUS-R | CTGCTGCCCGTAAGACGATT | |
| POP7-F | CACCGTGGAGCTTGTTGATG | |
| POP7-R | CCCTGAAGACTCGGATGTGG | |
| TRMT61A-F | GTGCTGCCACAGGTCTACAA | |
| TRMT61A-R | TTGGTGGCGAAGGTCAGGTA | |
| POLR2F-F | AGCATGCACCTATTCCAGTGGC | |
| POLR2F-R | ACAGGGAGATGGAGGGAAGGAT | |
| PPME1-F | AGGAGGTCATTCTGCCCTTTC | |
| PPME1-R | TGCCAACGTCTTTTGCCATTG | |
| CEP72-F | GGCGAGATTGTGGAACTGAAGC | |
| CEP72-R | GCAGGTGTTCATTGGTGCTGAC | |
| MYBL2-F | CACCAGAAACGAGCCTGCCTTA | |
| MYBL2-R | CTCAGGTCACACCAAGCATCAG | |
| CEP131-F | AACGAGCAGCTTTCTAGCGA | |
| CEP131-R | CTTCACCGCAGCCTCATGTT | |
| ORC1-F | CCCTATCAGTGGGGGACAGA | |
| ORC1-R | ATGGGGAGTAGAGGTCGCTT | |
| TK1-F | GCCAAAGACACTCGCTACAG | |
| TK1-R | CCCCTCGTCGATGCCTATG | |
| MCM10-F | TCAAGGAACTGATGGACCTGCC | |
| MCM10-R | CTCCAACATCCGCTGCTTCTGT | |
| qPCR-rs470-F | CCTCGGGGTAGACGTCTCTTT | |
| qPCR-rs470-R | GAGAGCGTCCATGAACTGGC | |
| For sgRNA vector construction | Primer sequence (5' → 3') | |
| sgRNA1-F | ccggAGATCGTGCCCGAGGGCGAG | |
| sgRNA1-R | aaacCTCGCCCTCGGGCACGATCT | |
| sgRNA2-F | ccggGCTGTTCGAGCACTACTACC | |
| sgRNA2-R | aaacGGTAGTAGTGCTCGAACAGC | |
| For reporter plasmid construction | Primer sequence (5' → 3') | |
| reporter-rs10076470G-F | CCGCTCGAGTAGGACAGCAGGTTTCCAGTTGCA | |
| reporter-rs10076470G-R | CCCAAGCTTGCCTGCAATACCTCCGTGACCATT | |
| reporter-rs10076470A-F | GTAGACGTCTCTTTCCTTTCTCAGGGCTGGGAAGGAG | |
| reporter-rs10076470A-R | CTCCTTCCCAGCCCTGAGAAAGGAAAGAGACGTCTAC | |
| For SNP identification | Primer sequence (5' → 3') | |
| SNP-rs10076470-F | CACGGTTCTCTGGCACTGTA | |
| SNP-rs10076470-R | TTGGGCTTATTCCTGGACGG | |
| For *Nsun2* knockout mice identification | | Primer sequence (5' → 3') |
| *Nsun2*-F1 | AGCCTGAGGGCGAGGAAGAC | |
| *Nsun2*-R1 | AAAGCGACAGGTGAGCAAGCT | |
| *Nsun2*-F2 | CCGAGACAGGATTTCTGGGTGTTCT | |
| *Nsun2*-R2 | GGGATCCAGTGCCCTCTTCTGAT | |
| *Nsun2*-Cre-F | ACCAGCCAGCTATCAACTCG | |
| *Nsun2*-Cre-R | TTACATTGGTCCAGCCACC | |
| For transfection | siRNA sequence (5' → 3') | |
| si*TIGAR* #1 | GUGAUCUCAUGAGGACAAA | |
| si*TIGAR* #2 | CCUACAGGAUCAUCUAAAU | |
| si*STAT1* #1 | GUGGCAAAGAGUGAUCAGA | |
| si*STAT1* #2 | GACCAUGCCUUUGGAAAGU | |

**Supplementary Table 8. The probe squences for EMSA**

| Probe | Sequence (5' → 3') |
| --- | --- |
| 374G-F-biotin | ACTCCATCTTAAGAAGAAGTCATAG |
| 374G-R-biotin | CTATGACTTCTTCTTAAGATGGAGT |
| 374A-F-biotin | ACTCCATCTTAAAAAGAAGTCATAG |
| 374A-R-biotin | CTATGACTTCTTTTTAAGATGGAGT |
| 375G-F-biotin | GTGGAAAGTTCCGGAGGTTAGTGGG |
| 375G-R-biotin | CCCACTAACCTCCGGAACTTTCCAC |
| 375A-F-biotin | GTGGAAAGTTCCAGAGGTTAGTGGG |
| 375A-R-biotin | CCCACTAACCTCTGGAACTTTCCAC |
| 470G-F-biotin | CCCAGCCCTGAGGAAGGAAAGAGAC |
| 470G-R-biotin | GTCTCTTTCCTTCCTCAGGGCTGGG |
| 470A-F-biotin | CCCAGCCCTGAGAAAGGAAAGAGAC |
| 470A-R-biotin | GTCTCTTTCCTTTCTCAGGGCTGGG |
| 086C-F-biotin | ATCCTCCGCGTCCTCCGGCCGCTGC |
| 086C-R-biotin | GCAGCGGCCGGAGGACGCGGAGGAT |
| 086T-F-biotin | ATCCTCCGCGTCTTCCGGCCGCTGC |
| 086T-R-biotin | GCAGCGGCCGGAAGACGCGGAGGAT |
| 374G-F | ACTCCATCTTAAGAAGAAGTCATAG |
| 374G-R | CTATGACTTCTTCTTAAGATGGAGT |
| 374A-F | ACTCCATCTTAAAAAGAAGTCATAG |
| 374A-R | CTATGACTTCTTTTTAAGATGGAGT |
| 375G-F | GTGGAAAGTTCCGGAGGTTAGTGGG |
| 375G-R | CCCACTAACCTCCGGAACTTTCCAC |
| 375A-F | GTGGAAAGTTCCAGAGGTTAGTGGG |
| 375A-R | CCCACTAACCTCTGGAACTTTCCAC |
| 470G-F | CCCAGCCCTGAGGAAGGAAAGAGAC |
| 470G-R | GTCTCTTTCCTTCCTCAGGGCTGGG |
| 470A-F | CCCAGCCCTGAGAAAGGAAAGAGAC |
| 470A-R | GTCTCTTTCCTTTCTCAGGGCTGGG |
| 086C-F | ATCCTCCGCGTCCTCCGGCCGCTGC |
| 086C-R | GCAGCGGCCGGAGGACGCGGAGGAT |
| 086T-F | ATCCTCCGCGTCTTCCGGCCGCTGC |
| 086T-R | GCAGCGGCCGGAAGACGCGGAGGAT |
